# Distinct p53 phosphorylation patterns in chronic lymphocytic leukemia patients are reflected in the activation of circumjacent pathways upon DNA damage

**DOI:** 10.1002/1878-0261.13337

**Published:** 2022-12-02

**Authors:** Veronika Mancikova, Michaela Pesova, Sarka Pavlova, Robert Helma, Kristyna Zavacka, Vaclav Hejret, Petr Taus, Jakub Hynst, Karla Plevova, Jitka Malcikova, Sarka Pospisilova

**Affiliations:** ^1^ Central European Institute of Technology (CEITEC) Masaryk University Brno Czech Republic; ^2^ Department of Internal Medicine – Hematology and Oncology, Faculty of Medicine Masaryk University and University Hospital Brno Czech Republic; ^3^ Institute of Medical Genetics and Genomics, Faculty of Medicine Masaryk University and University Hospital Brno Czech Republic

**Keywords:** CLL, p53, phosphorylation

## Abstract

*TP53* gene abnormalities represent the most important biomarker in chronic lymphocytic leukemia (CLL). Altered protein modifications could also influence p53 function, even in the wild‐type protein. We assessed the impact of p53 protein phosphorylations on p53 functions as an alternative inactivation mechanism. We studied p53 phospho‐profiles induced by DNA‐damaging agents (fludarabine, doxorubicin) in 71 *TP53*‐intact primary CLL samples. Doxorubicin induced two distinct phospho‐profiles: profile I (heavily phosphorylated) and profile II (hypophosphorylated). Profile II samples were less capable of activating p53 target genes upon doxorubicin exposure, resembling *TP53*‐mutant samples at the transcriptomic level, whereas standard p53 signaling was triggered in profile I. *ATM* locus defects were more common in profile II. The samples also differed in the basal activity of the hypoxia pathway: the highest level was detected in *TP53*‐mutant samples, followed by profile II and profile I. Our study suggests that wild‐type *TP53* CLL cells with less phosphorylated p53 show *TP53*‐mutant‐like behavior after DNA damage. p53 hypophosphorylation and the related lower ability to respond to DNA damage are linked to *ATM* locus defects and the higher basal activity of the hypoxia pathway.

## Graphical abstract

In chronic lymphocytic leukemia cells, increased activity of the hypoxic pathway and *ATM* locus defects can render wild‐type p53 hypophosphorylated. Such a phospho‐profile, termed profile II, cannot fully respond to DNA damage; accumulated p53 remains inactive and does not trigger transcription of its target genes upon stimulus, as compared to the standard phospho‐profile I. Thus, hypophosphorylated p53 resembles cases with *TP53* gene mutation.

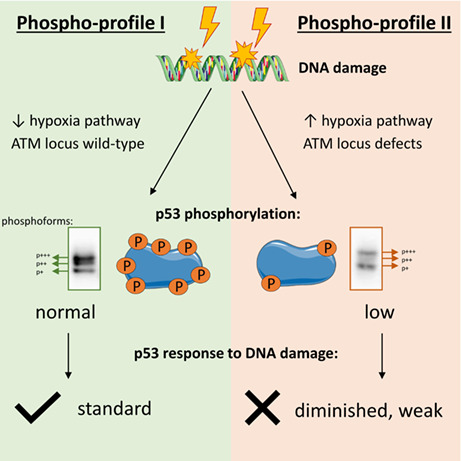

**Abbreviations**
CLL: chronic lymphocytic leukemia
FBS: fetal bovine serum
FCR: fludarabine + chlorambucil + rituximab
NGS: next‐generation sequencing
OS: overall survival
PFA: paraformaldehyde
progeny
: Pathway RespOnsive GENes
TTST: time to second treatment
VAF: variant allele frequency

## 1 Introduction

The p53 transcription factor exerts its central genome‐protecting role by coordinating a regulatory circuit that senses and reacts to a wide range of stimuli, including DNA damage, abnormal oncogenic signals, or hypoxia [1]. p53 protein's stability and activity are tightly regulated through a multitude of posttranslational modifications. To date, over 50 individual p53 posttranslational modifications produced by a wide range of stress‐sensing enzymes have been described. Significant differences exist in the modifications' spectra triggered by distinct stress‐inducing agents, creating a highly complex and flexible signaling network [2]. Diverse combinations of these modifications allow for fine‐tuning the cell response and eventually determine the final cell fate [3].

Phosphorylation belongs to the most essential p53 posttranslational modifications as it is crucial for protein stabilization and its consequent activity. Human p53 harbors an array of serine and threonine residues that can be phosphorylated by an extensive collection of kinases. Phosphorylation on the p53 N terminus shows a remarkable redundancy (multiple kinases can modify a single site, and a single kinase can phosphorylate multiple residues), highlighting the ‘fail‐proof’ layered regulation of the p53 pathway due to its central role in tumor suppression [3, 4]. Once activated, p53 triggers specific transcriptional programs that control cell cycle arrest, DNA damage response, cell metabolism, and apoptosis to prevent a potentially compromised cell from proliferation and, thus, propagation of mutations. Nevertheless, half of all human tumors escape this guardian mechanism by either direct mutations in the *TP53* gene or aberrations of other p53 pathway's components (e.g. MDM4 amplification [5]). However, the complete landscape of p53 pathway alterations operating in tumorigenesis is likely far from being fully portrayed.

Defects in the *TP53* gene represent the most important biomarker of chronic lymphocytic leukemia (CLL)—clinically and genetically highly heterogeneous and incurable disease. The *TP53* gene status (deletion of *TP53* locus 17p and/or *TP53* gene mutations) affects the prognosis of CLL patients and their response to therapy. Therefore, the *TP53* gene status testing has been introduced into routine clinical practice [6], and positive results provide grounds for applying targeted inhibitors of B‐cell receptor or Bcl2 pathways that have shown the ability to induce a response in these difficult‐to‐treat patients [7].

Apart from a direct genetic impairment, the p53 pathway can be dysregulated by other mechanisms. In this regard, it has been described that decreased p53 phosphorylation can lead to changes in protein conformation affecting interaction partners of p53 protein in breast tumors, resembling a cancer‐associated p53 mutated state [8]. However, whether alternative p53 phosphorylation plays a role in CLL pathogenesis remains to be explored.

Herein, we screened for the first time the p53 phosphorylation patterns of 71 *TP53*‐intact primary CLL samples treated by two DNA‐damaging agents (fludarabine, doxorubicin) and studied the impact of DNA damage on the CLL transcriptome. We describe that while fludarabine induces a relatively uniform phospho‐pattern, samples treated with doxorubicin show two different profiles. The transcriptomic analysis revealed that samples having one of these profiles fail to activate p53 signaling after DNA damage, resembling those with genetically impaired *TP53*.

## 2 Materials and methods

### 2.1 Human primary samples, cell lines, and culture conditions

The study has been approved by the Ethics committee of Masaryk University (number of ethics committee case EKV‐2018‐017). Eighty clinically characterized primary CLL samples were provided from the biobank of the Department of Internal Medicine—Hematology and Oncology, University Hospital Brno (CZ). In this biobank, all samples were collected after written informed patient's consent, approved by the hospital ethics committee, in accordance with the Declaration of Helsinki. All patients fulfilled the iwCLL/NCI diagnostic criteria for CLL [9]. Peripheral blood samples were processed by gradient centrifugation using Ficoll‐Paque PLUS (GE Healthcare, Chicago, IL, USA) combined with RosetteSep Kit (StemCell, Vancouver, Canada). Obtained high‐purity B lymphocytes (> 98%) were vitally frozen (viability after thawing > 80%). Seventy‐one samples with intact *TP53* were used to study phosphorylation patterns (Table 1). Nine samples with fully expanded biallelic defect of the *TP53* locus were used as controls in mRNA expression analyses (mutation variant allele frequency (VAF) range 88–100%; *TP53* mutation accompanied with either del(17p) or cn‐LOH 17p; Table S1).

**Table 1:** Clinico‐biological characteristics of the studied patients (*N* = 71) and association analyses between these characteristics and identified phospho‐profiles. Significant *P* values are in bold.

	Study cohort (*N* = 71)	Profile I (*N* = 33)	Profile II (*N* = 22)	*P*‐value
Gender				
Male (%)	48 (68)	26 (79)	9 (41)	**0.009**
Female (%)	23 (32)	7 (21)	13 (59)	
Age at diagnosis				
Median (range)	62.8 (43.2–85.4)	64.7 (43.5–82.8)	60.5 (43.2–85.4)	0.399
Status at sampling				
Never treated (%)	5 (7)	3 (9)	2 (9)	
Before treatment (%)	55 (77)	26 (79)	17 (77)	
After a therapy (%)	11 (16)	4 (12)	3 (14)	
RAI staging at diagnosis				
Low: 0 (%)	23 (32)	10 (35)	10 (53)	0.267^a^
Intermediate: I + II (%)	27 (38)	12 (41)	4 (21)	
High: III + IV (%)	12 (17)	5 (17)	2 (10)	
Unknown (%)	9 (13)	2 (7)	3 (16)	
Time to first treatment from diagnosis (days)				
Median (range)	1148 (34–8296)	728 (34–8170)	1589 (63–8296)	0.132^a^
IGHV status				
Unmutated (%)	48 (68)	22 (67)	15 (68)	1.0^b^
Mutated (%)	20 (28)	10 (30)	6 (27)	
Unknown (%)	3 (4)	1 (3)	1 (5)	
11q−^c^				
Yes (%)	24 (34)	5 (15)	12 (55)	**0.002** ^b^
No (%)	45 (63)	28 (85)	8 (36)	
Unknown (%)	2 (3)	0 (0)	2 (9)	
12+^c^				
Yes (%)	9 (13)	5 (15)	2 (9)	0.694^b^
No (%)	58 (82)	27 (82)	18 (82)	
Unknown (%)	4 (5)	1 (3)	2 (9)	
13q−^c^				
Yes (%)	39 (55)	17 (52)	14 (64)	0.253^b^
No (%)	30 (42)	16 (48)	6 (27)	
Unknown (%)	2 (3)	0 (0)	2 (9)	

^a^ Only calculated for the samples taken prior to starting any CLL‐related therapy.

^b^ Calculated only for those samples where data were available.

^c^ Assessed by FISH.

Once thawed, primary cells were kept in RPMI‐1640 medium (Biosera). Additionally, the HG3 cell line was used herein (a generous gift from Prof. R. Rosenquist, Sweden). HG3 is a cell line derived from a human CLL through EBV‐transformation with the wt‐*TP53* gene [10] and unmutated IGHV. HG3 was also maintained in the RPMI‐1640 medium. All media were supplemented with 10% (v/v) heat‐inactivated fetal bovine serum (FBS; Biosera, Nuaille, France) and 1% (v/v) penicillin/streptomycin (MP Biomedicals, Irvine, CA, USA). To induce DNA damage, cells were incubated with 1.5 μm doxorubicin or 15 μm fludarabine. HG3 cell line was treated for 1, 3, 6, 12, and 24 h; primary CLL cells were treated for 24 h.

### 2.2 Phos‐tag analysis and western blots

After treatment, cells were lysed in RIPA buffer (50 mm Tris pH 7.4, 150 mm NaCl, 0.1% SDS, 0.5% sodium deoxycholate, 1% NP‐40, 1 mm sodium vanadate, and 50 mm NaF). Half of the sample was directly heated with 2 × LDS loading buffer (ThermoFisher, Waltham, MA, USA), while the other half was treated with a 1 : 1 mixture of Alkaline phosphatase (ThermoFisher): λ protein phosphatase (New England Biolabs, Ipswich, MA, USA) for 30 min at 30 °C, and then heated with loading buffer. Lysates were then resolved by both SDS/PAGE and Phos‐tag PAGE. Phos‐tag (Wako Pure Chemical Industries, Richmond, VA, USA) analysis was performed according to the manufacturer's protocol using a neutral‐pH gel system and a Zinc(II) complex. The antibodies used in this study are listed in Table S2. Imaging and quantification of western blots were performed with a UVITEC imaging system and the imagej program.

### 2.3 Genetic characterization of the samples

Somatic hypermutations in the IGHV locus were routinely screened as described previously [11, 12]. Variants in the *TP53* gene were studied using in‐house amplicon‐based next‐generation sequencing (NGS) [13, 14]. Recurrent chromosomal aberrations (i.e., deletion of 17p13, 11q22.3, and 13q14.2, trisomy 12) were analyzed by FISH. To detect variants in 70 genes associated with lymphoid malignancies (Table S3) and additional chromosomal defects, targeted NGS was performed using a custom LYNX panel with the limit of detection of 5% VAF [15]. The somatic origin of all found variants in the *ATM* gene was verified by Sanger sequencing of germline DNA isolated from buccal swabs.

SNVs and indels in exons and adjacent splice sites were identified. Additionally, the 3'UTR region of *NOTCH1* and introns of *MYC* were covered and analyzed. Variants with a minimum coverage of 100×, ≥ 5 variant reads, and ≥ 5% VAF were called. Next, the functional impact of variants classified as missense, frameshift, in‐frame, splice donor/acceptor, start loss, and stop gain were analyzed further. Only variants with population frequency < 1% or unknown in the population databases gnomAD and 1000 genomes were considered. The information about detected variants in dbSNP, COSMIC, ClinVar, VarSome, and available literature was used during variant interpretation. Finally, frameshift variants were visually inspected in the IGV program to exclude potential artifacts.

CNVs were evaluated with the limit of detection of 20% and the resolution of 300 kB–1 Mb for recurrent deletions on 17p, 11q, and 13q loci and 6 Mb in the rest of the genome. For this study, we focused on relevant CLL‐related aberrations in chromosomes 11, 12, 13, and 17.

### 2.4 RNA isolation, library preparation, and NGS sequencing

RNA was isolated from CLL cells left intact in the culture medium for 24 h or maintained in 1.5 μm doxorubicin for 24 h. Total RNA was isolated using TRIzol (ThermoFisher) according to the manufacturer's instructions. The RNA integrity was assessed by the Fragment Analyzer system (Agilent, Santa Clara, CA, USA). Only RNAs with RIN > 7.0 were processed further. RNA‐Seq libraries were prepared using Lexogen QuantSeq 3' mRNA‐Seq Library Prep Kit FWD for Illumina with polyA selection and sequenced on Illumina NextSeq 500 sequencer (read length 1 × 75 nt). The adapters and quality trimming of raw fastq reads were performed using Trimmomatic v0.36 [16]. Trimmed RNA‐Seq reads were mapped against the human genome reference (hg38) annotations using star v2.7.3a [17]. UMIs were used for the deduplication of aligned reads [18]. Quality control after alignment concerning the number and percentage of uniquely and multi‐mapped reads, rRNA contamination, mapped regions, read coverage distribution, strand specificity, gene biotypes, and PCR duplication was performed using several tools, namely rseqc v2.6.2 [19], picard toolkit v2.18.27 and qualimap v.2.2.2 [20], and biobloom tools v 2.3.4‐6‐g433f [21].

#### 2.4.1 Differential expression analysis

The differential gene expression was calculated based on the gene counts produced using featurecounts tool v1.6.3 [22] and using bioconductor package deseq2 v1.20.0 [23]. Volcano plots were produced using the ggplot v3.3.3 package, and MA plots were generated using the ggpubr v0.4.0 package. Heatmap was generated from selected top differentially regulated genes using r package pheatmap v1.0.10. deseq2 normalized gene counts for all individual samples were visualized. Genes with baseMean coverage ≥ 25 and log2(fold‐change) ≥ 1 or ≤ −1 from comparisons of treated profile I versus control profile I, and treated profile II versus control profile II were considered. Ordering in such heatmap was determined by the biggest log2(fold‐change) differences between profile I and profile II in descending direction. Row scaling was applied to emphasize differences between conditions.

#### 2.4.2 progeny & dorothea

We used a footprint‐based method called progeny (Pathway RespOnsive GENes) [24, 25] to estimate signaling pathway activities based on consensus gene signatures obtained from perturbation experiments. progeny contains signatures for 14 signaling pathways (Androgen, EGFR, Estrogen, Hypoxia, JAK–STAT, MAPK, NFkB, p53, PI3K, TGFb, TNFa, Trail, VEGF, and WNT). The gene counts produced using featurecounts tool v1.6.3 were log2 transformed. Then, we inspected the log2(counts) distribution and removed transcripts with log2(counts) < 3, usually containing genes expressed under the RNAseq detection threshold. The cleaned data were normalized using vsn r package3 v3.60.0. Pathway activity score was calculated with the function *progeny* from the progeny r package v1.14.0 using the 100 most responsive genes per pathway. The unpaired two‐sided Student's *t*‐test was used to compare differences in the pathway activity between the conditions. Heatmaps were generated using the r package pheatmap v1.0.10.

Additionally, we used the dorothea r package v1.4.1 [26] to infer the HIF1A activity from the expression of its target genes. The dorothea is a curated, comprehensive resource built upon different types of evidence (literature‐curated resources, ChiP‐seq peaks, transcription factors' binding site motifs, and interactions inferred directly from gene expression).

### 2.5 Real‐time PCR analysis

The expression levels of p53 target genes *BAX*, *BBC3*, *CDKN1A*, and *GADD45A* were studied. First, 500 ng of total RNA isolated from treated and untreated cultivated cells was reverse‐transcribed using Superscript II (ThermoFisher) and oligo(dT)_14_ primer following the manufacturer's instructions. The level of target mRNA was quantified by real‐time PCR using taqman assays (ThermoFisher), taqman Gene Expression Master Mix (ThermoFisher), and the quantstudio 12 Flex Real‐Time PCR system (ThermoFisher). Assays were carried out in triplicates, and negative controls were included in all PCR series. The ΔΔ*C*_t_ method was used for the determination of mRNA content. The geometrical mean of house‐keeping genes *HPRT1A* and *TBP* cycle threshold (*C*_t_) was used as an internal standard.

For miRNA‐34a expression analysis, 4 ng of total RNA isolated from non‐cultivated untreated cells was reverse‐transcribed using taqman MicroRNA Assays (ThermoFisher) and specific primers for miRNA‐34a and RNU38B following the manufacturer's instructions. Quantification of miRNA was performed by real‐time PCR using taqman assays (ThermoFisher), ABsolute QPCR Mix, ROX (ThermoFisher), and 7500 Fast Real‐Time PCR System (ThermoFisher). All reactions were carried out in triplicates with respective negative controls. The obtained miRNA‐34a expression levels were normalized to RNU38B and interpreted as $2^{-\Delta C_{t}}*100\%$.

### 2.6 Whole‐exome sequencing

Sequencing libraries were prepared from 100 ng of DNA using TruSeq Exome Kit (Illumina, San Diego, CA, USA) according to the manufacturer's instructions and sequenced on NextSeq 500 machine (Illumina).

Raw sequencing data in fastq format were processed using the bcbio pipeline manager version 1.2.3. [27]. The pipeline consists of read trimming, performed by the atropos tool [28], read alignment to the human reference genome GRCh38, performed with bwa mem [29], samtools [30] and sambamba [31], and somatic variant calling performed by mutect2 [32], strelka2 [33], and vardict [34] variant callers. The resulting variants were annotated using the vep annotation software version 100.2 [35]. The resulting annotated VCF files were converted to a table format using an in‐house conversion script.

All detected somatic variants were manually filtered and inspected in the respective bam files using igv software [36].

### 2.7 Flow‐cytometric analysis

γ‐H2AX phosphorylation at Ser139 was assessed using flow cytometry. Representative samples from profile I (*N* = 4) and profile II (*N* = 8) were cultured for 30 min or 24 h *in vitro* with or without 1.5 μm doxorubicin. Afterward, cells were collected, fixed with 4% paraformaldehyde (PFA), permeabilized with 1× PBS, 5% FBS, and 0.5% Tween20, stained using anti‐phospho‐Histone H2AX (Ser139) primary antibody, clone JBW301 (Sigma, Burlington, MA, USA) and visualized with an AlexaFluor647‐conjugated secondary antibody (Invitrogen, Waltham, MA, USA). Samples were measured using FACS Verse flow cytometer (BD Biosciences, Franklin Lakes, NJ, USA). Data were analyzed in flowjo v.10 software.

### 2.8 Statistical analysis

All statistical analyses were performed using graphpad prism v5 (GraphPad Software, San Diego, CA, USA) and spss version 25 (Chicago, IL, USA). Specific statistical tests used for different study variables are described in the figure legends. All tests were two‐sided. The Gaussian distribution of data was assessed. The Kaplan–Meier survival analysis was used to assess the probability of time to second treatment (TTST) from the start of first‐line treatment to the initiation of second‐line therapy or death of any cause. Overall survival (OS) was estimated from the initiation of treatment to death of any cause. *P*‐values < 0.05 were considered statistically significant.

## 3 Results

### 3.1 Induction of p53 phosphorylation by DNA‐damaging agents in HG3 cells

**Fig. 1:**
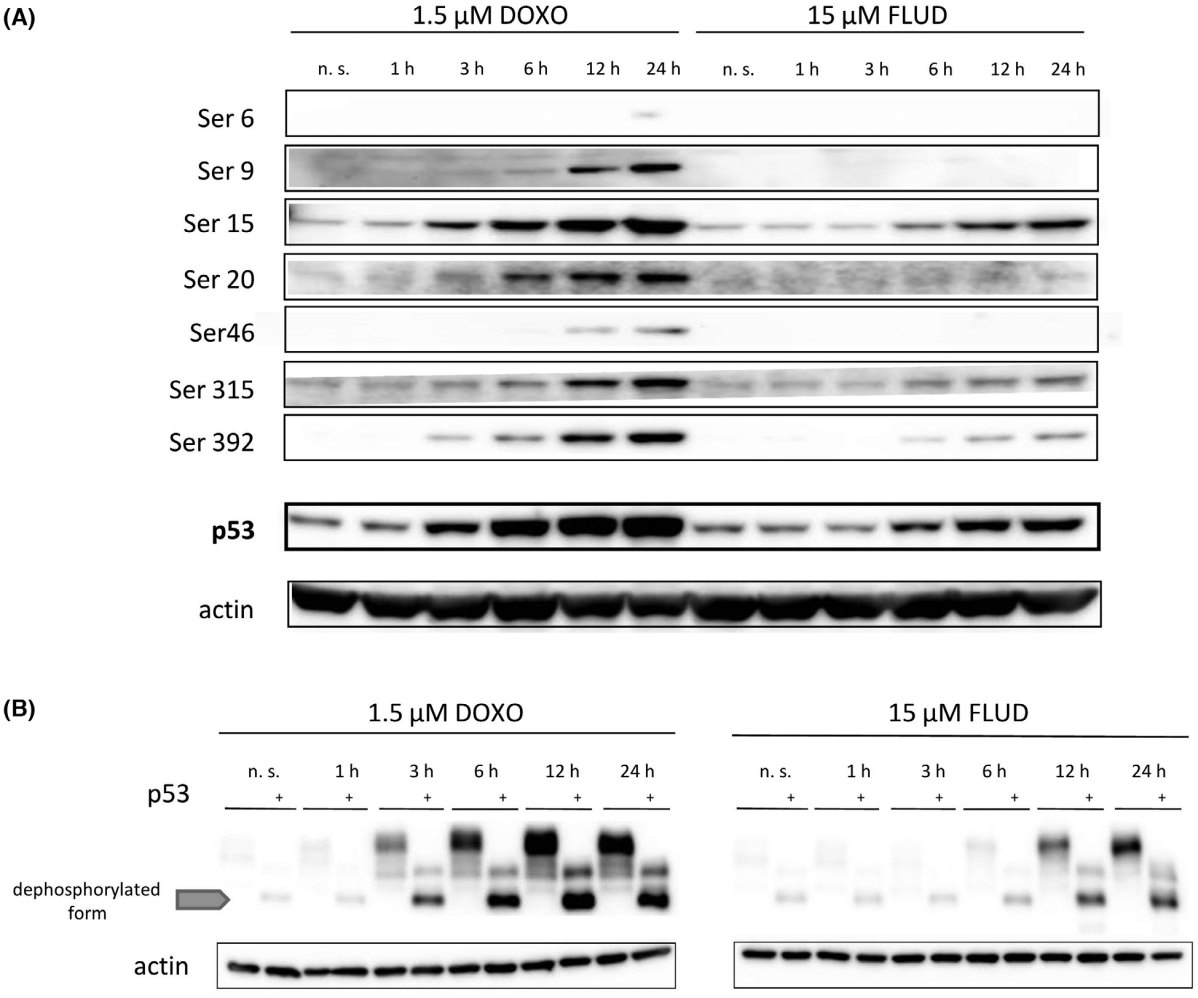
Effect of DNA damage‐inducing agents (doxorubicin, fludarabine) on p53 phosphorylation in the HG3 cell line. (A) HG3 cells were incubated with either 1.5 μm doxorubicin or 15 μm fludarabine for 1, 3, 6, 12, and 24 h, and subsequently lysed to extract proteins. Phosphorylation of serine 6, 9, 15, 20, 46, 315, and 392 was studied by the western blot analysis, which was also used to assess the protein level of total p53. Blots shown are representative of two technical replicates. The exposure times were as follows: Ser6–10 min, Ser9–5 min, Ser15–9 s, Ser20–5 min, Ser46–10 min, Ser315–25 s, Ser392–25 s, p53(total)–6 s. β‐actin was used as a loading control (exposure time 8 s). (B) Phos‐Tag analysis of protein lysates from (A). Each protein lysate was loaded untreated and treated with a mixture of phosphatases (marked with +). The phosphatase treatment serves as a control and reveals the dephosphorylated form of the studied protein. It is possible to appreciate that p53 in HG3 cells treated with the selected drugs is phosphorylated to such a high degree that only partial dephosphorylation was achieved. Residually phosphorylated isoforms are present above the unphosphorylated form, marked with a gray arrow. Images shown are representative of three technical replicates.

Under normal conditions, the level of p53 protein is kept low; however, it is readily stabilized and activated by phosphorylation upon stress [3]. Herein, we have applied two DNA‐damaging agents, doxorubicin and fludarabine, to induce stabilization of p53 *in vitro*. HG3 cells' exposure to these drugs led to a gradual increase in the p53 level accompanied by phosphorylation of different serine residues over 24 h (Fig. 1A). While we observed phosphorylation of all studied sites after doxorubicin, we only detected increased phosphorylation of serine 15, 315, and 392 after fludarabine treatment, which we attributed to the lower level of total p53 protein after the induction. Additionally, we have applied Zinc(II)‐Phos‐tag™ PAGE analysis to readily screen the complete phospho‐profile (Fig. 1B). This method provides characteristic separation patterns for phosphoforms according to the number and/or site of modifications [37]. A typical control in this electrophoretic method is treating the protein lysates with a mixture of phosphatases, which helps identify the dephosphorylated form of the studied protein. In our case, Zinc(II)‐Phos‐tag™ method revealed that both drugs caused abundant phosphorylation of the entire fraction of p53 protein, which was only partially eliminated by the phosphatases' treatment. For further *in vitro* experiments with primary CLL cells, we selected the most extended time point (24 h) when a significant p53 activation was observed for both drugs.

### 3.2 Primary CLL cells display two distinct p53 phospho‐profiles after doxorubicin treatment

**Fig. 2:**
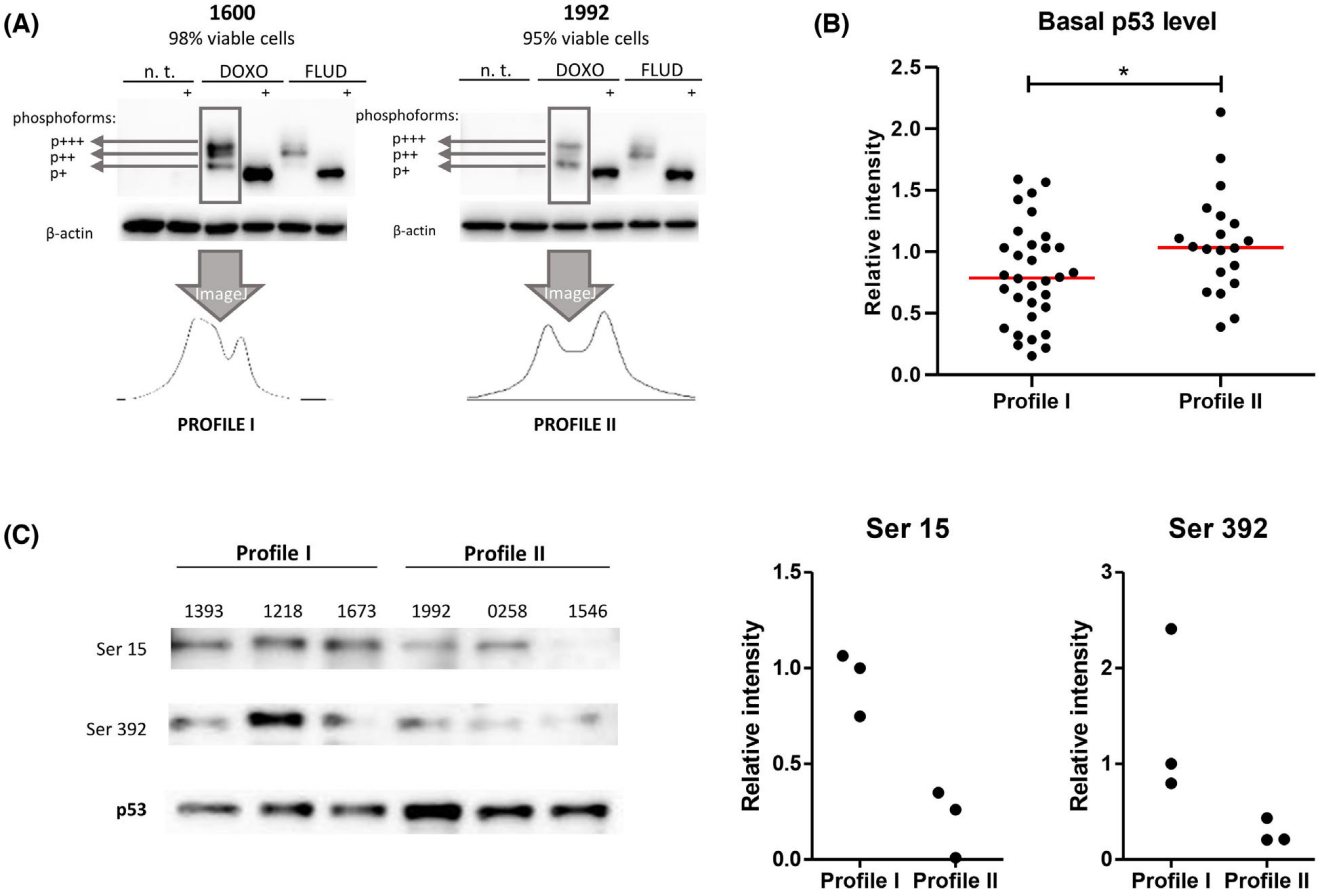
p53 Phospho‐profiling of primary CLL cells. (A) Doxorubicin and fludarabine induced distinct phospho‐profiles in the studied samples (*N* = 71), as assessed by Phos‐tag analysis and quantified using imagej. While there was a consistent pattern after fludarabine, doxorubicin induced two different phospho‐profiles, termed I and II. The most phosphorylated p53 phosphoform is marked as p+++, the least phosphorylated as p+. Phosphatase treatment (marked with +) was used to identify the unphosphorylated form of p53. Each sample was considered a biological replicate. (B) Western blot analysis was used to compare basal levels of p53 expression in protein lysates from unstimulated CLL cells cultured for 24 h (*N* = 52). Actin was used as a loading control to normalize the signal intensity. Profile II samples had significantly higher basal p53 expression (*P* = 0.039 [*], Mann–Whitney test). Each sample was considered a biological replicate. (C) Western blot analysis of six representative CLL samples was used to identify serine residues whose phosphorylation differs among the two phospho‐profiles. Only Ser15 and 392 of the panel of serine residues (Table S2) were evaluable. Total p53 was used as a loading control to normalize the signal intensity of phospho‐antibodies. All measurements were normalized to the signal detected in sample 1393. Images of serine 15 and 392 are quantified in the right panel of the figure. Mann–Whitney test was used to evaluate the statistical significance of the results (*P* = 0.100 for both sites, representative of three technical replicates).

In order to assess if alternative p53 phosphorylation plays a role in CLL pathogenesis, we used the Zinc(II)‐Phos‐tag™ PAGE to screen the p53 phospho‐profile of 71 clinically and biologically characterized CLL cases with the intact *TP53* gene (Table 1). All samples were treated separately with doxorubicin and fludarabine *in vitro*. Phos‐tag analysis revealed three major DNA damage‐induced phosphoforms of p53 in primary CLL cells (marked as phosphoform p+, p++, and p+++; Fig. 2A). Each of these is supposed to represent a p53 protein with different phosphorylation levels. We noticed marked differences in the phospho‐profiles caused by doxorubicin and fludarabine in primary CLL cells. In detail, the fludarabine‐induced pattern was relatively homogeneous among the screened samples, with phosphoform p++ being the most pronounced one in the majority of samples. Conversely, we identified two phospho‐profiles, termed I and II, after doxorubicin treatment. While phosphoforms p++ and p+++ were more abundant in profile I, the hypophosphorylated p + was the most prominent in profile II (Fig. 2A, more examples of phospho‐profiles are shown in Fig. S1). We also noticed that profile II samples had significantly higher basal p53 protein levels than profile I samples, where p53 was generally only detectable upon DNA damage (Fig. 2B and Fig. S2).

In representative samples, the profiles showed a trend to differ in the level of p53 phosphorylation at least at two sites: profile II was less phosphorylated at serine 15 and serine 392 (Fig. 2C). Out of the 71 screened CLL cases, we unequivocally assigned the doxorubicin‐induced profile in 55 samples (77%). No or minimal p53 stabilization was achieved for the remaining cases, which impeded profile assignment. Association analyses between the identified profiles and important clinico‐biological features are listed in Table 1. Profile II samples were enriched in those harboring deletions in 11q (*P* = 0.002). Additionally, profile II samples showed a trend toward lower basal miR‐34a expression when compared with profile I (Fig. S3).

### 3.3 CLL samples showing phospho‐profile II fail to activate the p53 signaling pathway under doxorubicin treatment

Next, we studied if the two distinct doxorubicin‐induced phospho‐profiles translate into transcriptomic differences in CLL cells exposed to doxorubicin. For the analysis by RNAseq, we have selected 11 representative samples with profile I, 10 samples with profile II, and 9 samples with biallelic defect of the *TP53* locus, the latter representing dysfunctional p53 (Table S1).

**Fig. 3:**
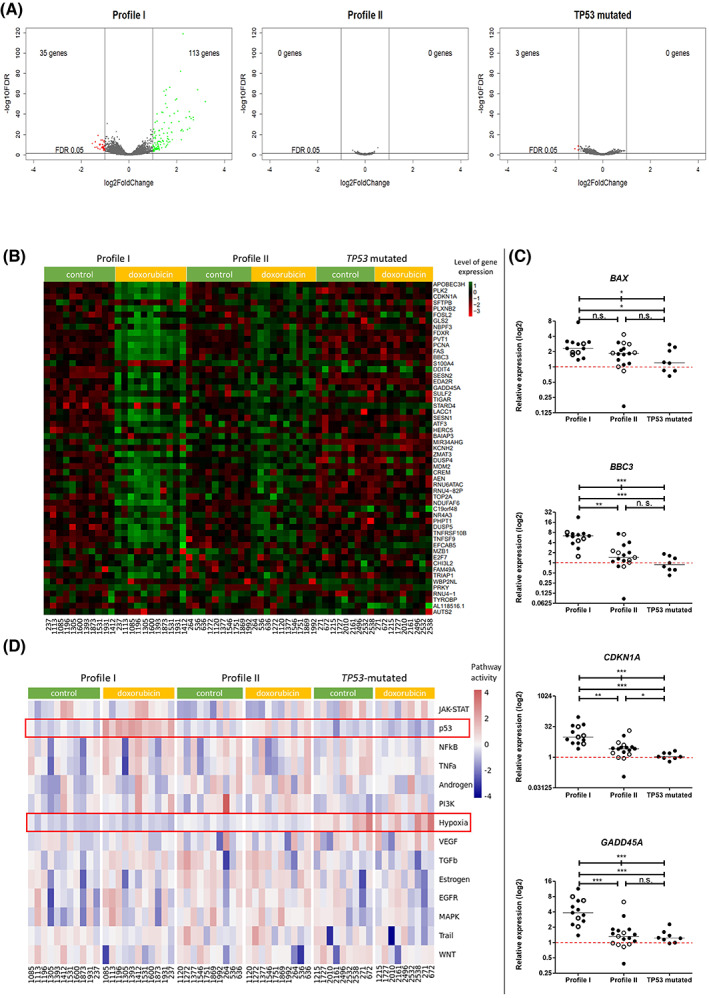
Transcriptomic analysis of doxorubicin‐induced phospho‐profiles. (A) Volcano plots representing results of differential expression analysis comparing untreated and treated paired samples within each experimental group by RNA sequencing (individual samples were considered biological replicates: Profile I *N* = 11 samples, profile II *N* = 10 samples, TP53 mutated *N* = 9 samples). Fold‐change is depicted on the *x*‐axis, while significance is on the *y*‐axis. Significantly downregulated genes after doxorubicin treatment (FDR ≤ 0.05 and fold change ≤ 0.5) are depicted in red, while upregulated (FDR ≤ 0.05 and fold‐change ≥ 2) are in green. Each dot in volcano plots represents the mean value for all samples in the designated group. Even though deseq2 analyzed over 32 000 data points in each of the three experimental groups, most of the data points had very similar or identical non‐significant FDR values and/or log change values in *TP53* mutant samples and profile II samples. Thus, the plots seem to depict fewer points. (B) Heat map of 55 genes (rows) showing the highest difference between differentially expressed genes in profile I and profile II samples by RNA sequencing analysis. Columns represent level of gene expression in individual patient samples in control or doxorubicin conditions. *TP53*‐mutated samples depict the state when p53 protein is not functional. (C) taqman qRT‐PCR validation of RNAseq findings. The validation included 27 samples included in the RNAseq (filled symbols) and 11 additional samples (4 profile I and 7 profile II samples, open symbols), run in triplicates for each gene assayed. The expression of *BAX*, *BBC3*, *CDKN1A*, and *GADD45A* was calculated relative to the mean of two housekeeping genes using the ΔΔ*C*_t_ method. Mann–Whitney test was used for comparing two out of three groups, while all three groups were compared by the Kruskal–Wallis test. *P*‐values < 0.05 are coded as *, those < 0.01 as ** and < 0.001 as ***. n.s., non‐significant. Horizontal red dashed lines at *y* = 1 depict no response to treatment. (D) progeny analysis of transcriptomic data. Activity of selected pathways (rows) in each patient sample (columns) is shown in basal and doxorubicin‐treated conditions. In the basal state, all three groups significantly differ in the activity of the hypoxia pathway (*P* = 0.009 for profile I vs. profile II comparison, *P* = 0.0009 for profile I vs. *TP53* mut, and *P* = 0.003 for profile II vs. *TP53* mut). After doxorubicin treatment, the activity of the hypoxia pathway does not change further in either of the studied groups. Moreover, the p53 pathway is most significantly activated by doxorubicin in profile I samples (*P* = 1.7 × 10^−8^), followed by profile II (*P* = 0.028) and *TP53 mut* (*P* = 0.51), where no significant activation of the pathway was observed.

First, we compared untreated and doxorubicin‐treated conditions in paired samples within each experimental group (profile I, profile II, and *TP53*‐mutated samples). This analysis revealed 113 significantly upregulated and 35 significantly downregulated genes in samples from profile I after doxorubicin treatment (FDR < 0.05; log2fc ≥ ¦1¦). The differentially expressed genes identified in profile I were enriched in the p53 signaling pathway (*P*_Adjusted_ = 1.6 × 10^−8^), as shown by the DAVID functional annotation analysis [38]. Surprisingly, no such genes and only three significantly downregulated genes were identified in profile II and *TP53*‐mutated samples, respectively (Fig. 3A, Table S4).

Next, we searched for the most differentially expressed genes after doxorubicin treatment between profiles I and II (Fig. 3B, top 55 genes). The heatmap showed an apparent change in mRNA levels of these genes upon treatment in profile I samples, while no such change was observed in *TP53*‐mutated samples. Profile II samples could be characterized by an intermediate pattern (Fig. 3B). Upon closer inspection, many of the top 55 genes belonged to the p53 pathway (*BBC3*, *CDKN1A*, *FDXR*, *GADD45A*, etc.).

These findings were validated by taqman qRT‐PCR assays in an extended cohort of 38 CLL RNA samples, composed of 27 samples initially included in the RNAseq and 11 additional samples (4 profile I and 7 profile II samples). We assessed the expression of *BAX*, *BBC3*, *CDKN1A*, and *GADD45A*, the four known downstream effectors of the p53 signaling pathway [39]. We observed a significantly lower induction of expression of all selected genes in *TP53*‐mutated and profile II samples upon doxorubicin treatment, as opposed to a higher induction in profile I samples (Fig. 3C; *P* = 0.04, < 0.0001; < 0.0001 and 0.0001 for *BAX*, *BBC3*, *CDKN1A*, and *GADD45A*, respectively; Kruskal–Wallis test). After fludarabine treatment, the differences were not so prominent; however, profile II samples again tend to show intermediate induction of expression (Fig. S4).

Besides the standard differential gene expression analysis, we additionally applied the progeny [24] package to assess the overall activity of selected cancer‐related pathways. Compared to conventional pathway analysis methods, this footprint‐based approach is well generalizable across experimental conditions and reflects the effects of posttranslational modifications such as phosphorylation. This approach confirmed the previous findings concerning different activation of the p53 pathway among the patient subgroups (Fig. 3D). Additionally, this method revealed that basal activity of the hypoxia pathway in untreated cells significantly differed among our experimental groups; the highest activity of the hypoxia pathway was found in *TP53*‐mutated cells, followed by intermediate levels in profile II samples, and the lowest activity of the hypoxia pathway was in profile I. This pattern was also maintained upon treatment; DNA damage did not have any additional effect on the activity of the hypoxia pathway (Fig. 3D, Table S5). Differentially affected genes of the hypoxia pathway, as calculated by progeny, are depicted in Fig. S5A, and listed in Table S6. Additionally, we have used another resource, dorothea [26], to confirm our conclusions regarding the hypoxia pathway. The latter approach allowed us to calculate the activity of individual transcription factors (e.g. HIF1A) by looking at the expression patterns of their targets. This analysis confirmed the dysregulation of HIF1A among our experimental groups – HIF1A was the most active in *TP53*‐mutated cells, followed by profile II samples and HIF1A activity was the lowest in profile I (Fig. S5B).

Finally, we explored whether the biological differences between the two phospho‐profiles affected the clinical outcome of the patients. To analyze the potential differences in treatment response, we assessed the remission duration as a function of time to second treatment. As different treatment regimens have different effectiveness and response rates, we analyzed a sub‐cohort of patients uniformly treated by the chemoimmunotherapy regimen fludarabine + chlorambucil + rituximab (FCR; *N* = 20) that represented a standard‐of‐care in this retrospective cohort. We were not able to show differences between patients assigned in the two phosho‐profiles (Fig. S6A). For overall survival (OS), patients were stratified as to whether they had received targeted inhibitor treatment at any time during the course of the disease. We confirmed that this treatment strategy improved the outcome of the patients regardless of the profile, but no difference between profile I and profile II was observed (Fig. S6B).

### 3.4 Profile II samples are enriched with *ATM* locus and *MED12* aberrations

**Fig. 4:**
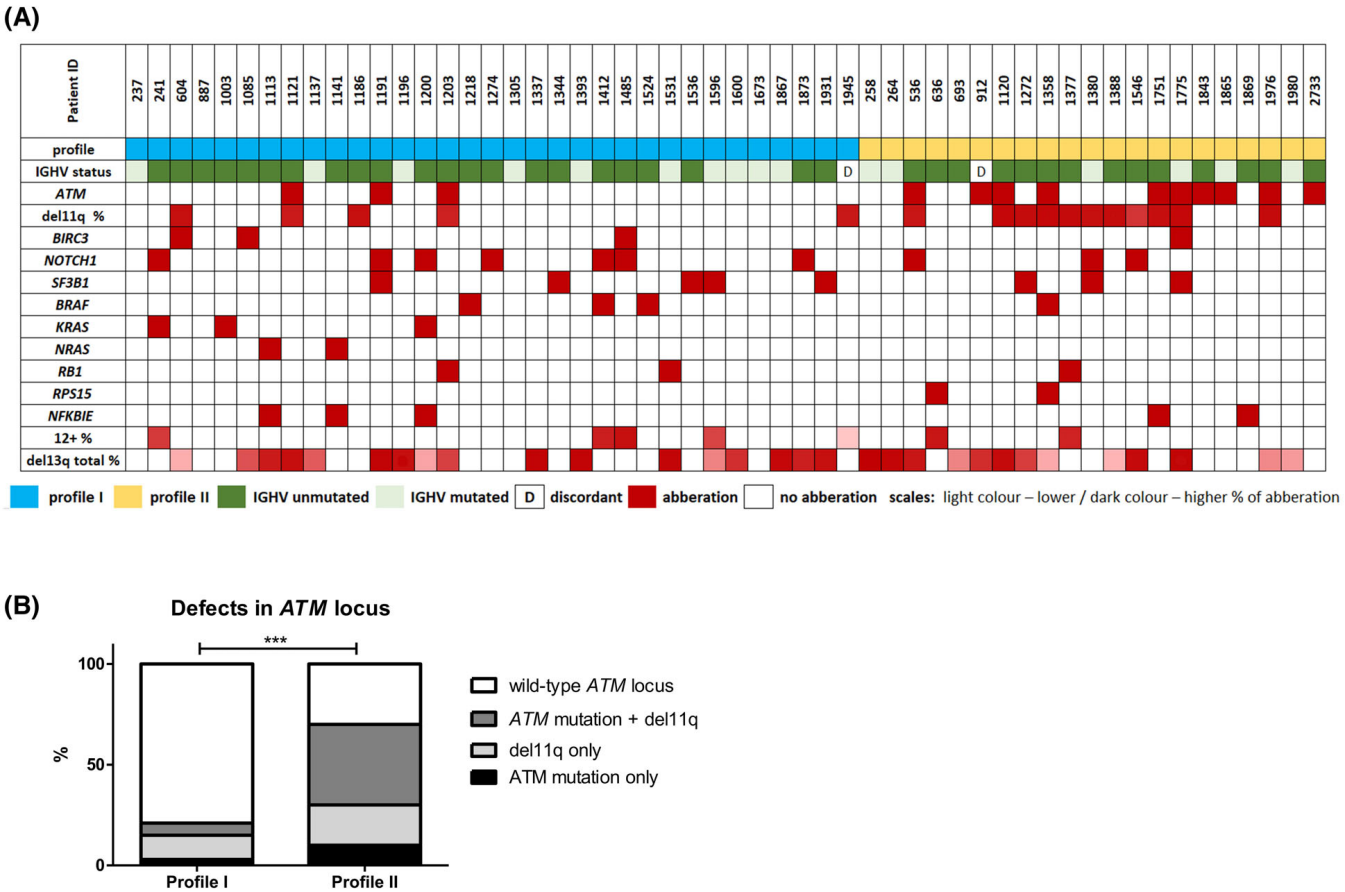
(A) Overview of results from NGS targeted gene panel focused on lymphoid malignancies. This panel covered 70 genes and was applied in all but one sample with a clearly determined profile (54 samples in total were sequenced). (B) Comparison of the presence of various *ATM* locus defects in profile I and profile II samples (*P* = 0.0001 [***], Fisher's exact test).

In order to gain more insight into the possible genetic drivers of the observed phospho‐patterns, targeted NGS of tumor DNA was applied. In total, 70 genes (Table S3) associated with lymphoid malignancies were studied in all but one sample with a clearly determined profile. Generated data allowed not only to identify SNVs and indels but also recurrent CLL‐related CNVs (Fig. 4A). The two studied profiles differed only in aberrations affecting *ATM* locus [*ATM* gene mutation and/or del(11q22.3)] – these were significantly more frequent in profile II (Fig. 4B, Table S7). Since ATM is a central kinase in sensing double‐strand breaks, we assessed the level of phosphorylation of γ‐H2AX (Ser139) as a read‐out of ATM activity and DNA damage. Profile II samples showed a mild, albeit non‐significant, dampening of overall DNA damage signaling (Fig. S7).

Furthermore, in five patients from profile II without identified *ATM* defects, we performed exome sequencing to analyze other aberrations that could potentially contribute to the profile II phenotype. Interestingly, in 2/5 patients, we detected somatic mutations in the *MED12* gene. These pathogenic variants in *MED12* were previously reported to be recurrent in CLL patients [40]. In addition, a somatic mutation in *MED12L*, a *MED12* paralog, was found in another patient (Table S8).

## 4 Discussion

The tumor suppressor protein p53, encoded by the *TP53* gene localized on chromosome 17, plays a key role in the pathology of chronic lymphocytic leukemia (CLL). As its genetic inactivation by either a locus deletion and/or gene mutations is directly associated with chemo‐refractoriness [41, 42], it is one of the few CLL biomarkers routinely analyzed in the clinical practice. Defective protein phosphorylation has also been shown to induce a mutant‐like p53 behavior [8]. Herein, we studied if alternative posttranslational phosphorylation can impair the function of wild‐type p53 protein in CLL. To confirm the hypothesis, we induced p53 phosphorylation by two DNA‐damaging drugs in a large set of *TP53* wild‐type primary CLL samples and screened the p53 phospho‐patterns. We were able to associate hypophosphorylated profile II with disrupted activation of p53 signaling as assessed by RNA sequencing and real‐time PCR analysis. Moreover, we linked this p53‐mutant‐like state with a higher activity of the hypoxia pathway and defects in the *ATM* gene locus.

Under stress‐free conditions, p53 protein's stability and activity are tightly regulated and kept low [43] through MDM2‐mediated timely degradation [44]. For this master regulatory loop, the N‐terminal end of p53 is essential. If it is unmodified, MDM2 readily binds it [45], triggering p53 ubiquitination and proteasomal degradation. N‐terminus is targeted by a plethora of stress‐sensing kinases, which phosphorylate it at multiple sites upon various forms of DNA damage, thus increasing the protein half‐life [46]. In this regard, chemotherapeutic drugs, including purine analogs (such as fludarabine) or topoisomerase inhibitors (such as doxorubicin), have been shown to increase p53 level in CLL cells effectively [47, 48]. We observed differences in the p53 phospho‐profiles induced by fludarabine or doxorubicin in CLL cells. Moreover, doxorubicin treatment led to two distinct phospho‐profiles. This confirms the different mechanisms of action of both used drugs, which is in line with previously published findings [49]. The altered signaling resulting in p53 hypophosphorylation in profile II after doxorubicin is likely not crucial for fludarabine response, as we observed a rather homogeneous pattern of p53 phosphorylation after fludarabine. Besides, reduced phosphorylation of p53, as we observe in profile II (after doxorubicin treatment), has been related to a state that resembles mutated p53 [8]. Having this in mind, CLL phospho‐profile II could encompass those samples that bear genetically wild‐type p53, but whose activity is impaired at the protein level by inadequate posttranslational modifications.

Indeed, we observed that, like the samples carrying an inactivated *TP53* gene, profile II samples failed to trigger p53 signaling upon DNA damage on the transcriptomic level. In this regard, it has already been described that wild‐type p53 can undergo conformational changes into a mutant form with an unavailable DNA‐binding domain and is thus incapable of induction of its target genes [50, 51]. In detail, phospho‐profile II could represent an intermediate state between wild‐type and mutant p53 since the induction of the studied downstream effector genes after doxorubicin treatment was much lower in profile II than in profile I samples, but still higher than in *TP53*‐mutated samples. In line with this, the expression of miR‐34a, whose downregulation is a well‐known indicator of deleted and/or mutated *TP53* gene [52], also showed this intermediate expression pattern in profile II samples.

In order to uncover the underlying mechanisms plausibly giving rise to the different p53 phospho‐profiles, we next used progeny to assess the basal activity of selected cancer‐related pathways in untreated cells. This analysis pointed to the importance of the hypoxia pathway, an established inducer of p53 accumulation [53]. We detected its highest activity in *TP53*‐mutated samples, which is in line with the recent findings [54]. Correspondingly with the above‐mentioned, the hypoxia pathway's activity in profile II samples was between the high levels found in *TP53* mutants and low activity in profile I. Under hypoxic conditions, p53 is not appropriately degraded through the MDM2‐mediated process [53], leading to its elevated protein levels that accumulate in the cell. In this scenario, p53 is known to be hypophosphorylated and transcriptionally inactive [51]. Our data suggest that elevated basal activity of the hypoxia pathway in profile II samples could contribute to the presence of clearly detectable levels of hypophosphorylated p53 protein in these cells and also to the p53 inability to respond to DNA damage caused by doxorubicin. Thus, increased hypoxia could contribute to the emergence of phospho‐profile II and its p53‐mutant‐like character. Given the increasing evidence of the importance of the hypoxia pathway in CLL pathogenesis and its potential druggability [54, 55], our results point to the possibility of hypoxia pathway targeting even in wt‐*TP53* patients.

Besides, we noticed that profile II samples were enriched in those harboring *ATM* defects. ATM is a kinase that senses and reacts to DNA double‐strand brakes and stabilizes p53 through phosphorylation, especially at Ser15 [56]. Although the ATM‐p53 axis is disrupted in most profile II samples, double‐strand breaks' sensing was only mildly affected, and we were still able to detect p53 protein in primary CLL cells after doxorubicin treatment. It suggests that p53 must be stabilized via an alternative pathway. Apart from ATM, DNA‐PK is involved in response to DNA double‐strand brakes [56, 57, 58]. DNA‐PK was reported to be overexpressed in CLL cells with del11q (encompassing *ATM* gene) [57], and DNA‐PK activity was described to be crucial for the survival of primary CLL cells with *ATM* defects [58]. In detail, after exposing cells to DNA damage, DNA‐PK might act via DNA‐PK/AKT/GSK3/MDM2 axis resulting in MDM2 hypophosphorylation and, consequently, p53 accumulation [56]. p53 stabilized this way (in the absence of a fully functional ATM) was reported to be hypophosphorylated on Ser15 [56], which is in line with our results. Thus, the activity of an alternative DNA damage response pathway after using doxorubicin could contribute to p53 accumulation in *ATM* defective samples. Moreover, *ATM* loss results in chronic oxidative stress, which might be responsible for the increased biogenesis of the HIF1 protein, a key component of the hypoxia pathway [59]. This finding can thus partially explain the observed increased activity of the hypoxia pathway in profile II *ATM*‐defective samples.

Mutations in *MED12/MED12L* genes could also be of importance since they were found in 3 of 5 profile II patients not having *ATM* defects. This high proportion is noteworthy, considering that mutations in the *MED12* gene were previously described in 5–8% of CLL cases [40, 60, 61]. *MED12* is a part of the mediator kinase module complex involved in p53 signal transduction, more specifically, it is a stimulus‐specific positive coregulator of p53 target genes [62]. Moreover, it has been shown that mutation in *MED12* lead to downregulation of p53 signaling [63].

Although we clearly demonstrated biological differences between the two identified phospho‐profiles, the impaired function of the p53 pathway in profile II was likely overcome by other mechanisms *in vivo*. The differences between profiles did not translate into the clinical outcome; neither the time to second treatment nor overall survival differed between the respective patient subgroups. The lack of difference in remission duration can be explained by the small sample size as only sub‐cohort of patients treated with the same regimen (FCR) could be compared. Moreover, the FCR regimen has a different mechanism of action compared to doxorubicin alone and other mechanisms might compensate for the insufficient p53 pathway activity. This is even more valid for overall survival because patients receive multiple treatment lines and different treatment regimens during the course of the disease.

## 5 Conclusions

Our study highlights the importance of correct p53 phosphorylation to perform its tumor suppressor roles in primary CLL cells properly. We describe a complex regulatory circuit in which higher hypoxic activity and impaired DNA double‐strand breaks' sensing lead to hypophosphorylation of p53 and accumulation of this dysfunctional form in CLL cells, rendering them less responsive to acute DNA damage.

## Conflict of interest

The authors declare no conflict of interest.

## Author contributions

SPo, SPa, JM, and KP designed the study. VM, MP, and RH performed western blot analyses, real‐time analyses, prepared libraries for RNAseq and LYNX panel sequencing and performed data analyses. KZ performed whole‐exome sequencing. VH analyzed the RNAseq data. PT provided progeny analysis. JH and MP analyzed LYNX panel NGS data. All authors contributed to the drafting of the manuscript.

### Peer review

The peer review history for this article is available at https://publons.com/publon/10.1002/1878‐0261.13337.

## Supporting information


**Fig. S1.** Phosphorylation patterns detected by Zn(II) Phos‐Tag technique.

**Fig. S2.** Western blot analysis of basal p53 protein levels.

**Fig. S3.** Relative miR34‐a expression levels in uncultured primary CLL cells.

**Fig. S4.** qRT‐PCR of p53 targets after fludarabine treatment.

**Fig. S5.** Activity of hypoxia pathway and HIF1A transcription factor.

**Fig. S6.** Patients' clinical outcome in relation to phospho‐profiles.

**Fig. S7.** H2AX phosphorylation.

**Table S1.** Overview of samples carrying *TP53* aberrations.

**Table S2.** Antibodies used in the study.

**Table S3.** List of NGS panel target genes focused on lymphoid malignancies.

**Table S4.** List of differentially expressed genes identified when untreated and doxorubicin‐treated conditions in paired samples within each experimental group were compared.

**Table S5.** List of progeny *P* values.

**Table S6.** List of hypoxia‐related genes used in progeny analysis.

**Table S7.** List of variants detected by targeted NGS panel (LYNX).

**Table S8.** List of validated somatic variants detected by whole‐exome sequencing.

**Data S1.** Supplementary material and legends.

## Data Availability

Further data are available upon request.
