# Supplementary material for: Distinct p53 phosphorylation patterns in chronic lymphocytic leukemia patients are reflected in the activation of circumjacent pathways upon DNA damage
Source: Mol Oncol. 2022 Dec 2;17(1):82–97. doi: 10.1002/1878-0261.13337 (PMC9812841; doi:10.1002/1878-0261.13337)

Supplementary Figure S1. Phosphorylation patterns detected by Zn(II) Phos-Tag technique (line p53 phospho highlighted in red). A.1-2. profile I after doxorubicine treatment. B.1-2. profile II after doxorubicine treatment. no tr = no treatment, doxo = doxorubicin, flud = fludarabine. -,+ with or without phosphatase treatment, respectively. X marks conditions that were not run.

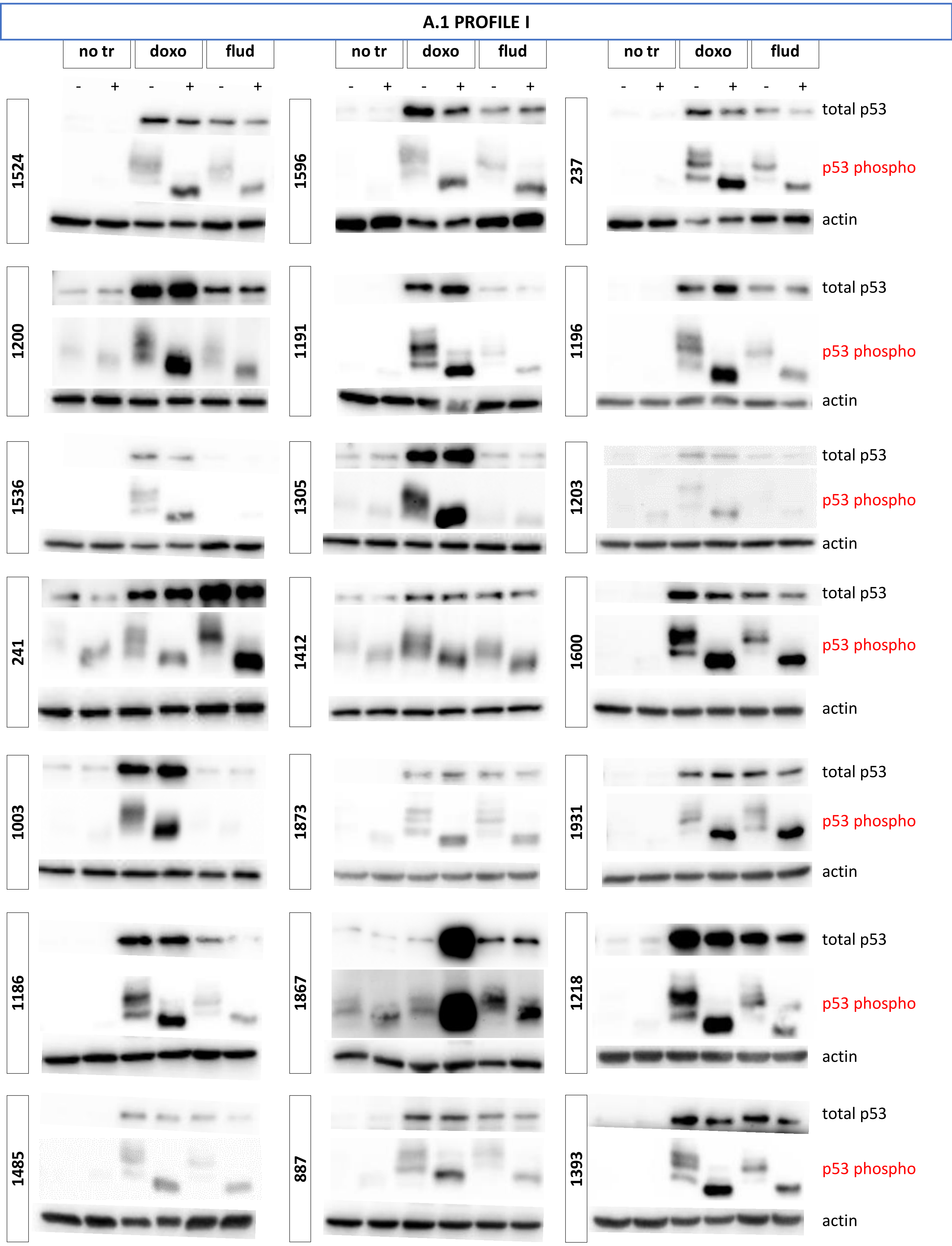

A.2 PROFILE I

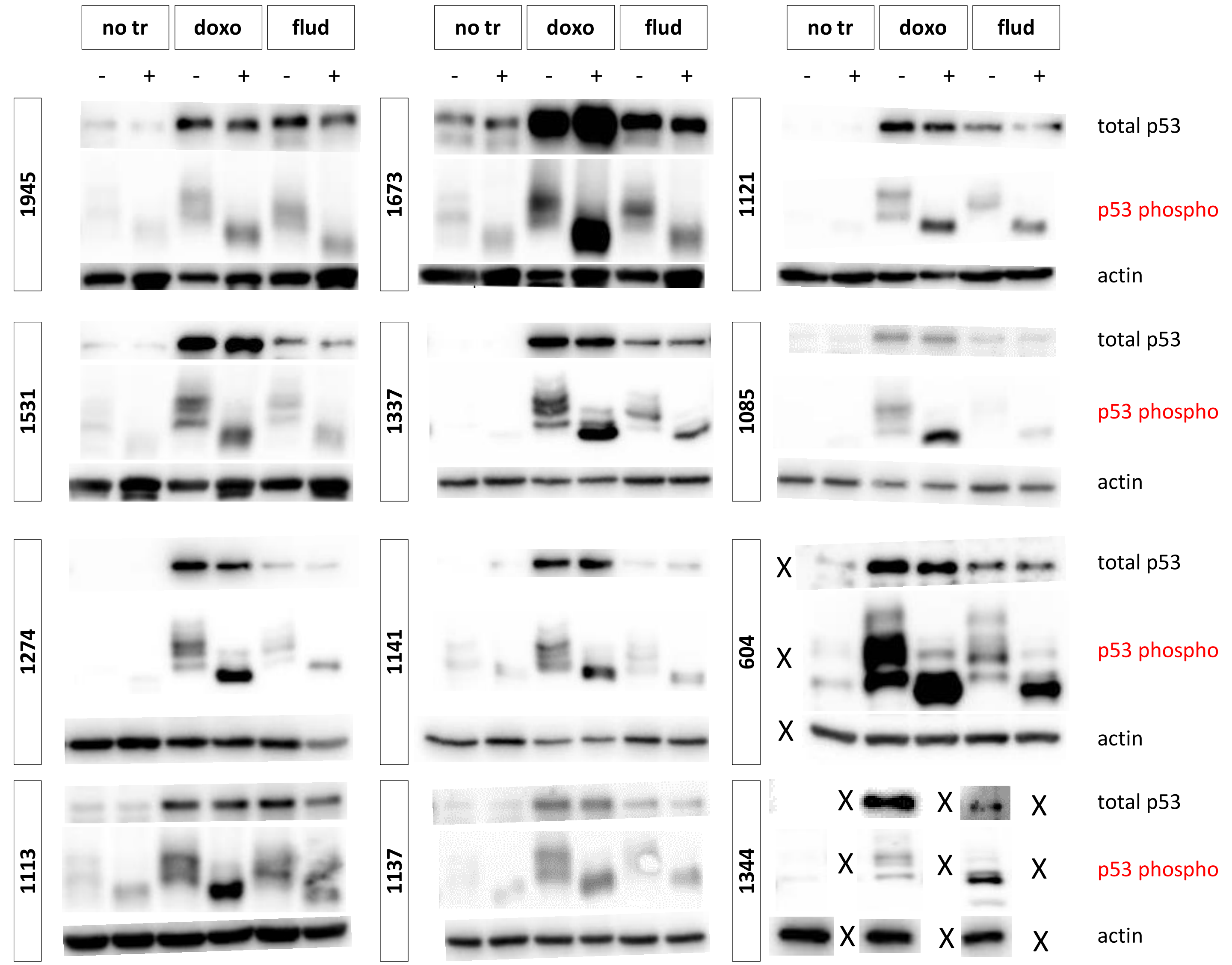

B.1 PROFILE II

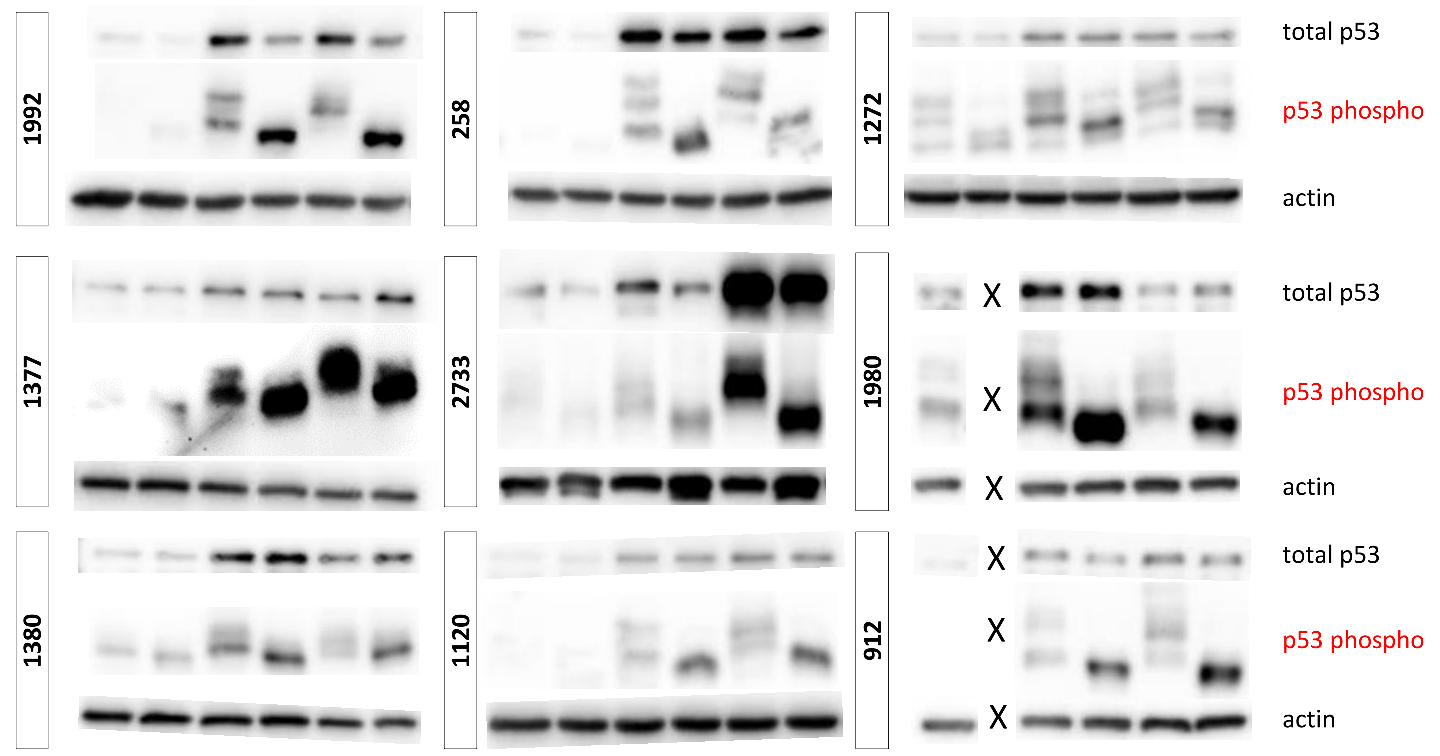

B.2 PROFILE II

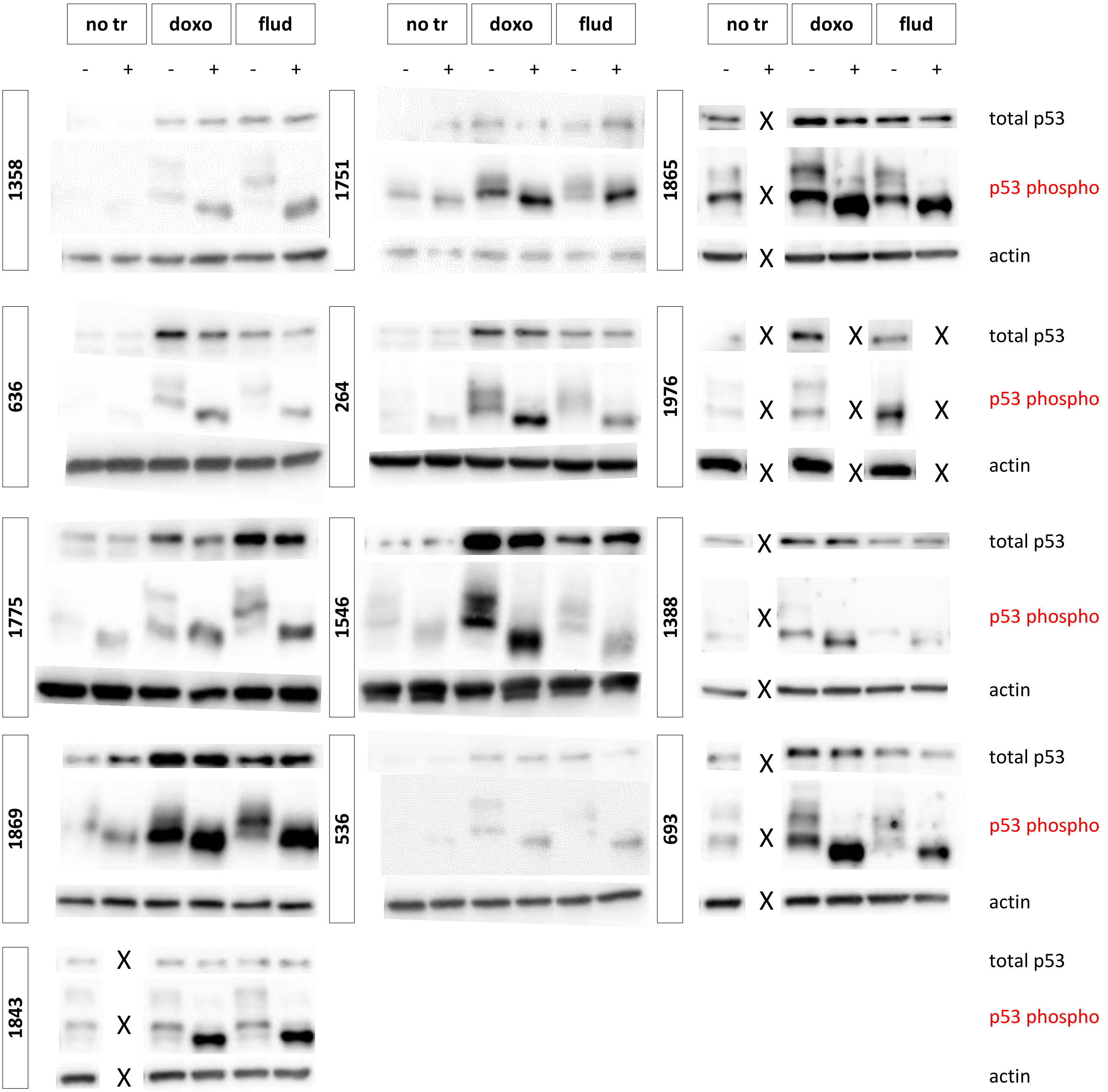

Supplement: Supplementary file 1 — Fig. S1. Phosphorylation patterns detected by Zn(II) Phos‐Tag technique. [file MOL2-17-82-s016.pdf]
