# Supplementary material for: Distinct p53 phosphorylation patterns in chronic lymphocytic leukemia patients are reflected in the activation of circumjacent pathways upon DNA damage
Source: Mol Oncol. 2022 Dec 2;17(1):82–97. doi: 10.1002/1878-0261.13337 (PMC9812841; doi:10.1002/1878-0261.13337)

Supplementary Figure S2. Western blot analysis of basal p53 protein levels. BRNO1215 *TP53* mutated sample was used as a positive contol with a strong p53 induction without any treatment. P1 and P2 refers to profile I and profile II sample groups, respectively. Actin was used as a loading control.

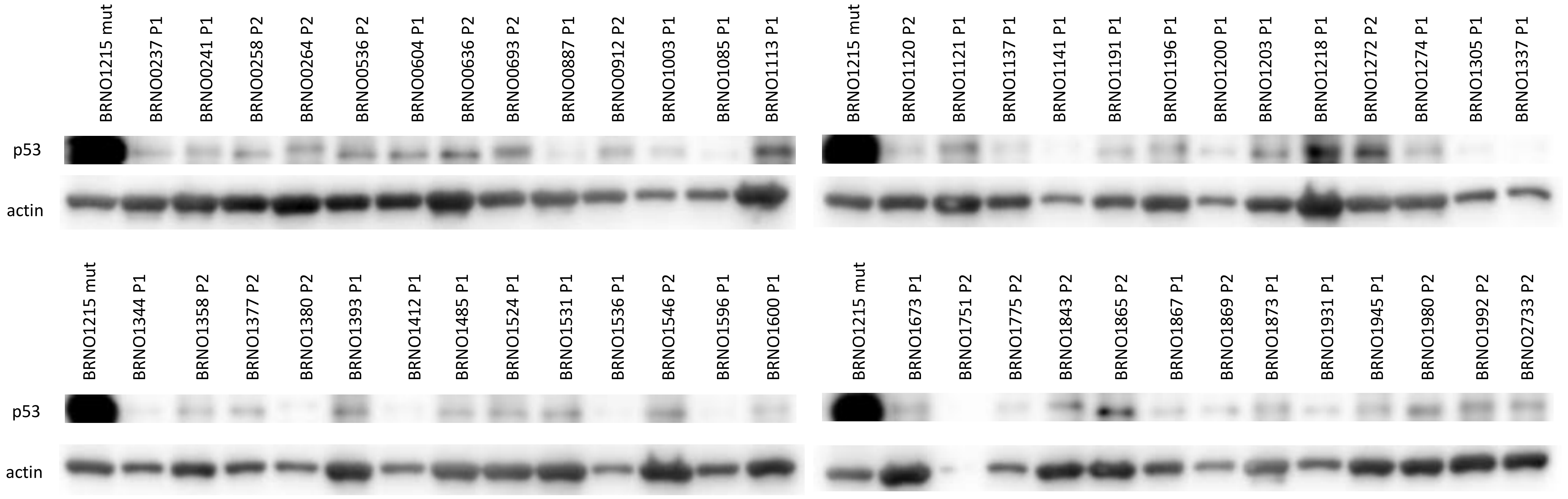

Supplement: Supplementary file 2 — Fig. S2. Western blot analysis of basal p53 protein levels. [file MOL2-17-82-s012.pdf]
