# Supplementary material for: Distinct p53 phosphorylation patterns in chronic lymphocytic leukemia patients are reflected in the activation of circumjacent pathways upon DNA damage
Source: Mol Oncol. 2022 Dec 2;17(1):82–97. doi: 10.1002/1878-0261.13337 (PMC9812841; doi:10.1002/1878-0261.13337)
Supplement: Supplementary file 3 — Fig. S3. Relative miR34‐a expression levels in uncultured primary CLL cells. [file MOL2-17-82-s014.pptx]

## Slide 1
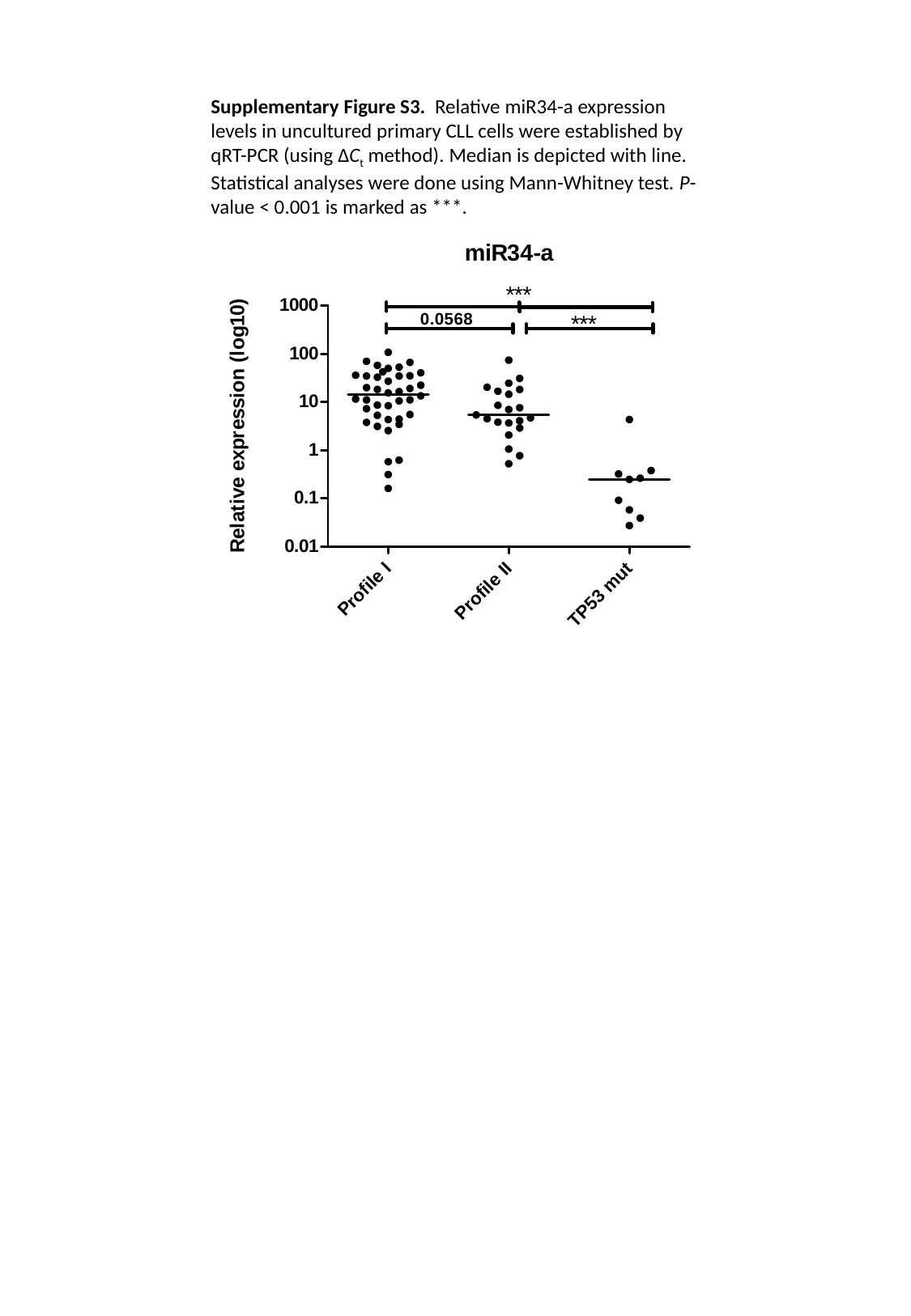

Supplementary Figure S3. Relative miR34-a expression levels in uncultured primary CLL cells were established by qRT-PCR (using ΔCt method). Median is depicted with line. Statistical analyses were done using Mann-Whitney test. P-value < 0.001 is marked as ***.
