# Supplementary material for: Distinct p53 phosphorylation patterns in chronic lymphocytic leukemia patients are reflected in the activation of circumjacent pathways upon DNA damage
Source: Mol Oncol. 2022 Dec 2;17(1):82–97. doi: 10.1002/1878-0261.13337 (PMC9812841; doi:10.1002/1878-0261.13337)
Supplement: Supplementary file 4 — Fig. S4. qRT‐PCR of p53 targets after fludarabine treatment. [file MOL2-17-82-s004.pptx]

## Slide 1
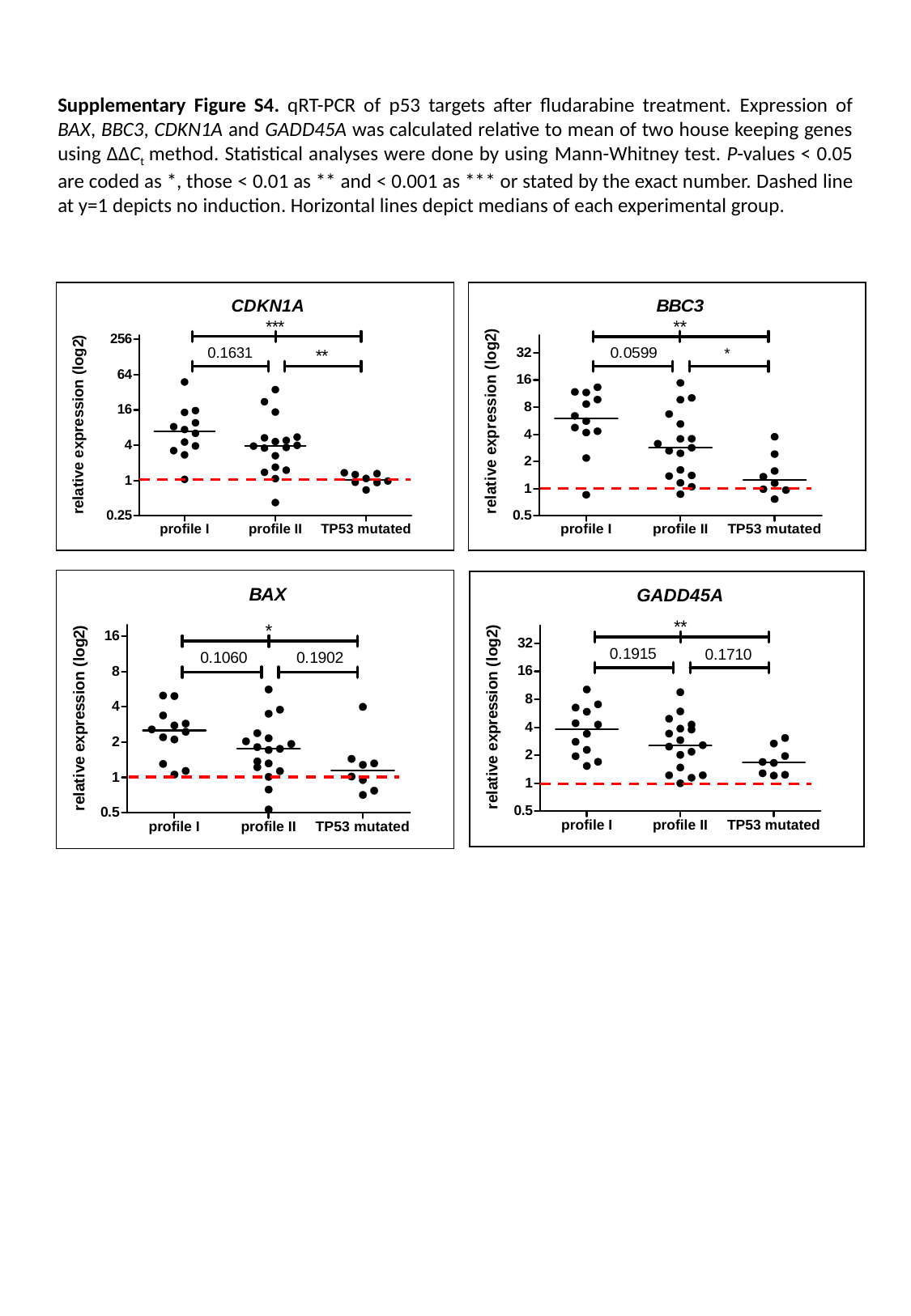

Supplementary Figure S4. qRT-PCR of p53 targets after fludarabine treatment. Expression of BAX, BBC3, CDKN1A and GADD45A was calculated relative to mean of two house keeping genes using ΔΔCt method. Statistical analyses were done by using Mann-Whitney test. P-values < 0.05 are coded as *, those < 0.01 as ** and < 0.001 as *** or stated by the exact number. Dashed line at y=1 depicts no induction. Horizontal lines depict medians of each experimental group.
