# Supplementary material for: Distinct p53 phosphorylation patterns in chronic lymphocytic leukemia patients are reflected in the activation of circumjacent pathways upon DNA damage
Source: Mol Oncol. 2022 Dec 2;17(1):82–97. doi: 10.1002/1878-0261.13337 (PMC9812841; doi:10.1002/1878-0261.13337)
Supplement: Supplementary file 5 — Fig. S5. Activity of hypoxia pathway and HIF1A transcription factor. [file MOL2-17-82-s015.pptx]

## Slide 1
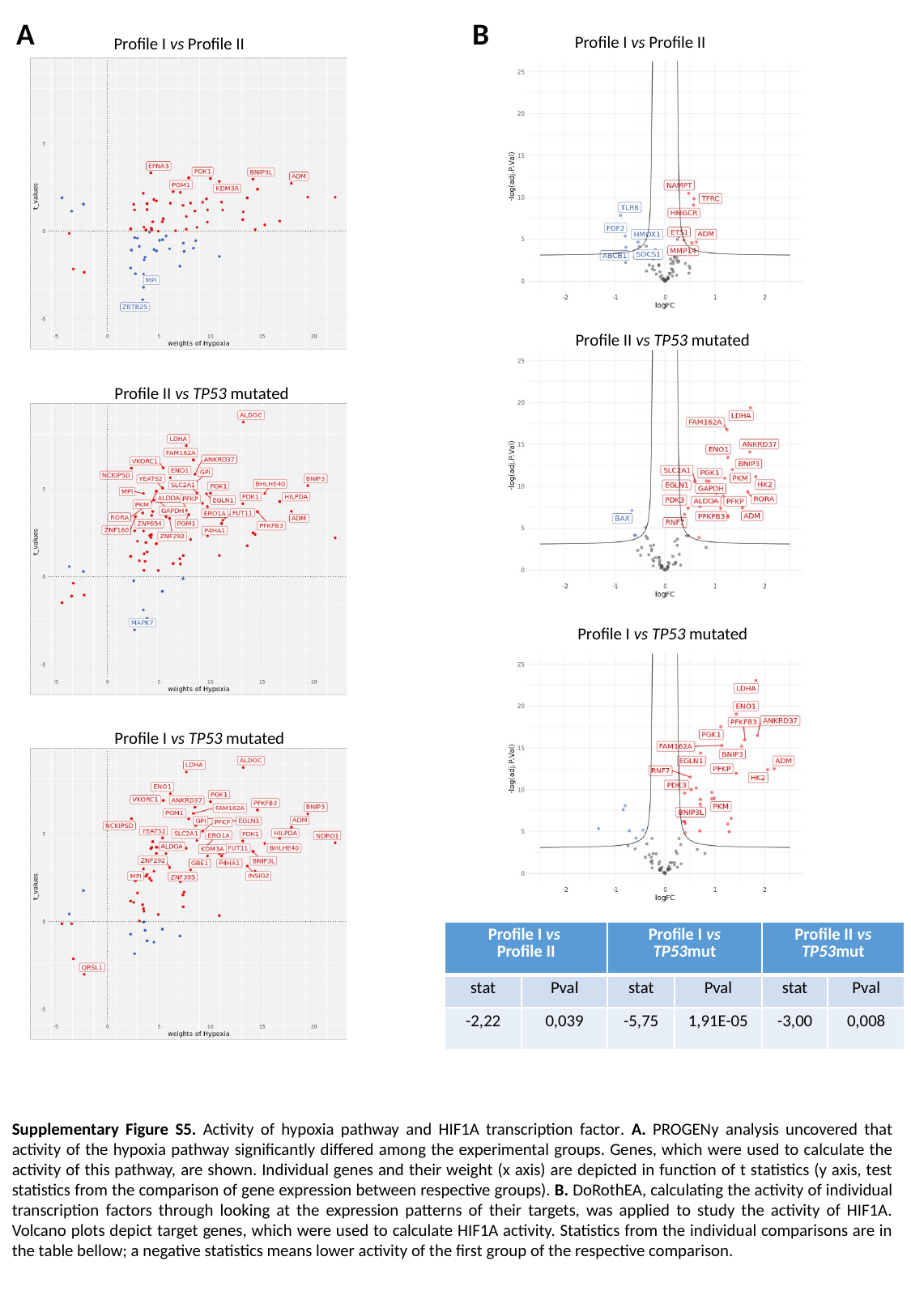

A
B
Profile I vs Profile II
Profile I vs Profile II
Profile II vs TP53 mutated
Profile II vs TP53 mutated
Profile I vs TP53 mutated
Profile I vs TP53 mutated
| Profile I vs Profile II | | Profile I vs TP53mut | | Profile II vs TP53mut | |
| --- | --- | --- | --- | --- | --- |
| stat | Pval | stat | Pval | stat | Pval |
| -2,22 | 0,039 | -5,75 | 1,91E-05 | -3,00 | 0,008 |
Supplementary Figure S5. Activity of hypoxia pathway and HIF1A transcription factor. A. PROGENy analysis uncovered that activity of the hypoxia pathway significantly differed among the experimental groups. Genes, which were used to calculate the activity of this pathway, are shown. Individual genes and their weight (x axis) are depicted in function of t statistics (y axis, test statistics from the comparison of gene expression between respective groups). B. DoRothEA, calculating the activity of individual transcription factors through looking at the expression patterns of their targets, was applied to study the activity of HIF1A. Volcano plots depict target genes, which were used to calculate HIF1A activity. Statistics from the individual comparisons are in the table bellow; a negative statistics means lower activity of the first group of the respective comparison.
