# Supplementary material for: Distinct p53 phosphorylation patterns in chronic lymphocytic leukemia patients are reflected in the activation of circumjacent pathways upon DNA damage
Source: Mol Oncol. 2022 Dec 2;17(1):82–97. doi: 10.1002/1878-0261.13337 (PMC9812841; doi:10.1002/1878-0261.13337)
Supplement: Supplementary file 6 — Fig. S6. Patients' clinical outcome in relation to phospho‐profiles. [file MOL2-17-82-s013.pptx]

## Slide 1
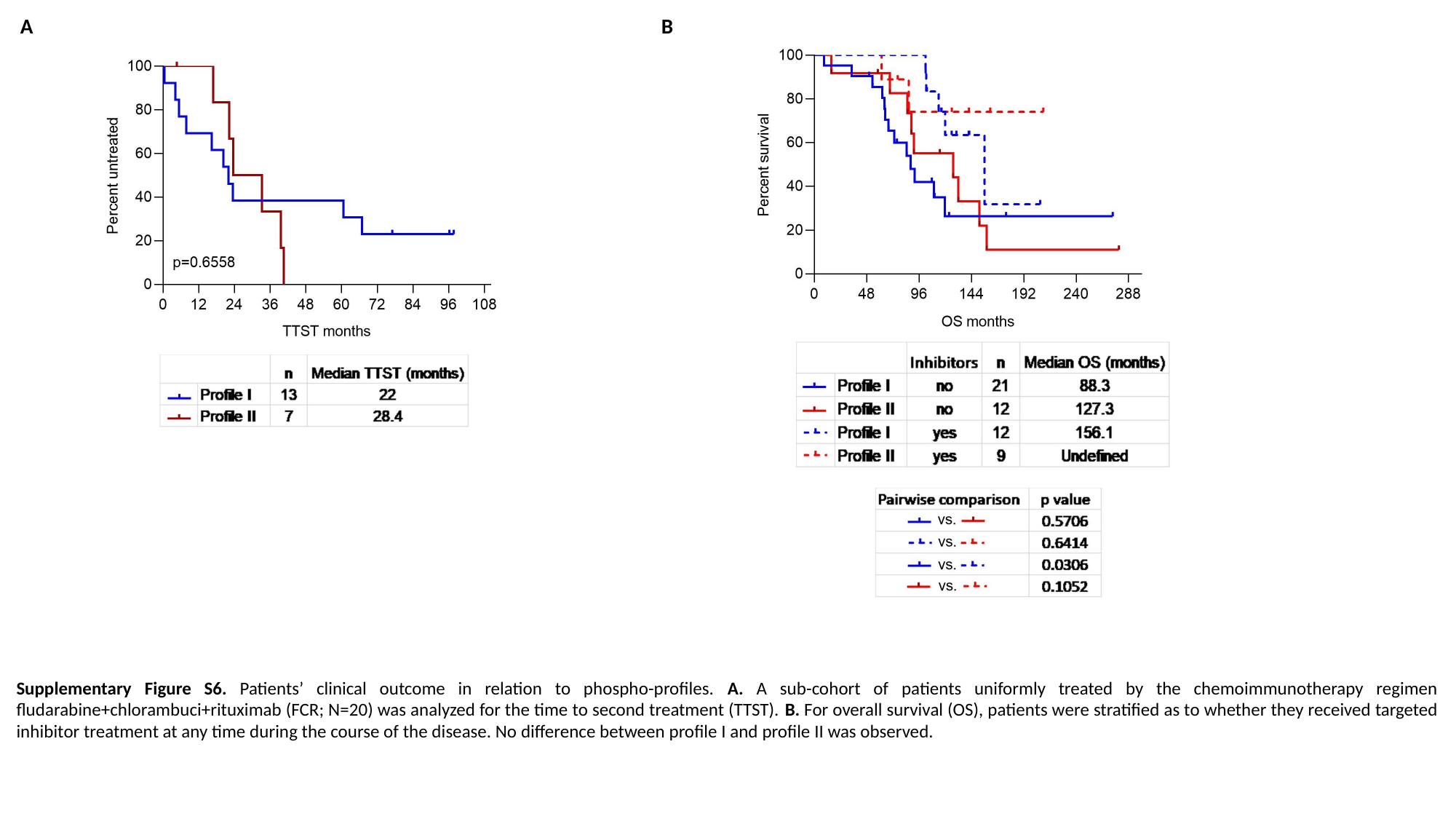

A
B
Supplementary Figure S6. Patients’ clinical outcome in relation to phospho-profiles. A. A sub-cohort of patients uniformly treated by the chemoimmunotherapy regimen fludarabine+chlorambuci+rituximab (FCR; N=20) was analyzed for the time to second treatment (TTST). B. For overall survival (OS), patients were stratified as to whether they received targeted inhibitor treatment at any time during the course of the disease. No difference between profile I and profile II was observed.
