# Supplementary material for: Distinct p53 phosphorylation patterns in chronic lymphocytic leukemia patients are reflected in the activation of circumjacent pathways upon DNA damage
Source: Mol Oncol. 2022 Dec 2;17(1):82–97. doi: 10.1002/1878-0261.13337 (PMC9812841; doi:10.1002/1878-0261.13337)
Supplement: Supplementary file 7 — Fig. S7. H2AX phosphorylation. [file MOL2-17-82-s010.pptx]

## Slide 1
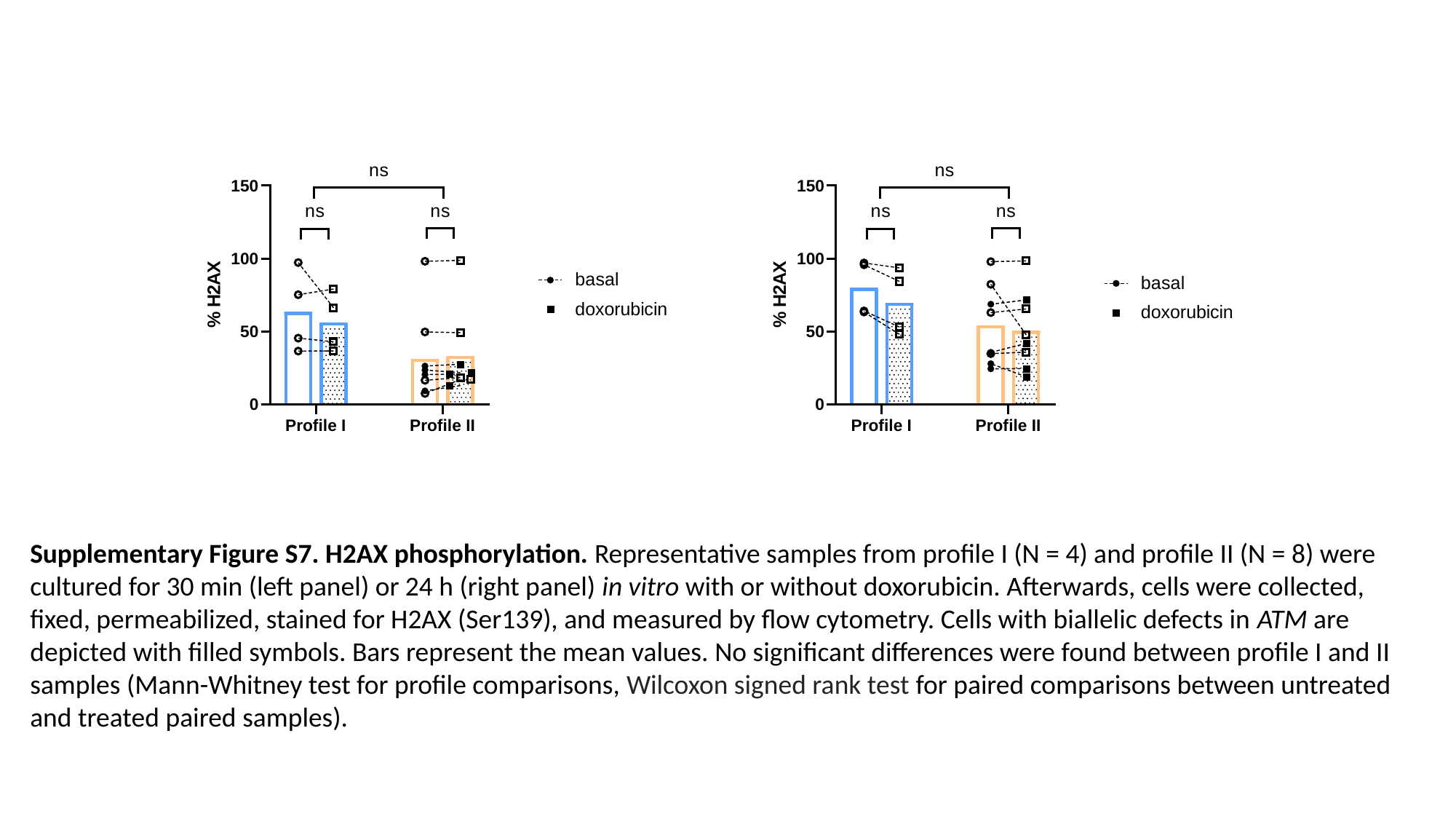

Supplementary Figure S7. H2AX phosphorylation. Representative samples from profile I (N = 4) and profile II (N = 8) were cultured for 30 min (left panel) or 24 h (right panel) in vitro with or without doxorubicin. Afterwards, cells were collected, fixed, permeabilized, stained for H2AX (Ser139), and measured by flow cytometry. Cells with biallelic defects in ATM are depicted with filled symbols. Bars represent the mean values. No significant differences were found between profile I and II samples (Mann-Whitney test for profile comparisons, Wilcoxon signed rank test for paired comparisons between untreated and treated paired samples).
