# Supplementary material for: Distinct p53 phosphorylation patterns in chronic lymphocytic leukemia patients are reflected in the activation of circumjacent pathways upon DNA damage
Source: Mol Oncol. 2022 Dec 2;17(1):82–97. doi: 10.1002/1878-0261.13337 (PMC9812841; doi:10.1002/1878-0261.13337)
Supplement: Supplementary file 8 — Table S1. Overview of samples carrying TP53 aberrations. [file MOL2-17-82-s009.docx]

| **ID** | ***TP53* mutation** | | | **del 17p (FISH)** | |
| --- | --- | --- | --- | --- | --- |
|  | **variant in c.DNA (LRG_321t1)** | **variant in protein (LRG_321p1)** | **cumulative VAF (%)** | **yes/no** | **frequency (%)** |
| 1215 | c.830G>T | p.Cys277Phe | 98.9 | No* | 0 |
| 2161 | c.919+1G>T; c.673-2A>G | p.?; p.? | 99.0 | Yes | 93 |
| 271 | c.797G>A | p.Gly266Glu | 99.5 | No* | 0 |
| 2496 | c.783-1G>T | p.? | 99.7 | No* | 0 |
| 672 | c.321C>A | p.Tyr107Ter | 99.4 | Yes | 86 |
| 1727 | c.741_742delinsTT | p.Arg248Trp | 93.8 | Yes | 87 |
| 2010 | c.569C>T; c.672+1G>T | p.Pro190Leu; p.? | 97.3 | Yes | 77 |
| 2532 | c.559+5G>A; c.626_627del | p.?; p.Arg209LysfsTer | 87.5 | Yes | 85 |
| 2538 | c.742C>T; c.287del | p.Arg248Trp; p.Ser96LeufsTer | 94.9 | Yes | 87 |

**Supplementary Table S1:** Overview of samples carrying *TP53* aberrations. VAF variant allele frequency. * Biallelic inactivation of *TP53* gene by the mechanism of copy-neutral loss-of-heterozygosity.
