# Supplementary material for: Distinct p53 phosphorylation patterns in chronic lymphocytic leukemia patients are reflected in the activation of circumjacent pathways upon DNA damage
Source: Mol Oncol. 2022 Dec 2;17(1):82–97. doi: 10.1002/1878-0261.13337 (PMC9812841; doi:10.1002/1878-0261.13337)
Supplement: Supplementary file 9 — Table S2. Antibodies used in the study. [file MOL2-17-82-s006.pdf]

Supplementary Table S2: Antibodies used in the study.

Target	Distributor	Distributor's catalogue number
p53 [DO-1]	abcam	#ab204452
β-actin	Sigma-Aldrich	#A5441
Phospho-p53 (Ser6)	Cell Signaling	#9285
Phospho-p53 (Ser9)	Cell Signaling	#9288
Phospho-p53 (Ser15)	Cell Signaling	#9284
Phospho-p53 (Ser20)	Cell Signaling	#9287
Phospho-p53 (Ser20)	Cell Signaling	#9287
Phospho-p53 (Ser46)	Cell Signaling	#2521
Phospho-p53 (Thr81)	Cell Signaling	#2676
Phospho-p53 (Ser315)	Cell Signaling	#2528
Phospho-p53 (Ser392) 9281	Cell Signaling	#9281
Goat Anti-Rabbit IgG (H + L)-HRP Conjugate	Bio-Rad	#1706515
Goat Anti-Mouse IgG (H + L)-HRP Conjugate	Bio-Rad	#1706516
