# Supplementary material for: Distinct p53 phosphorylation patterns in chronic lymphocytic leukemia patients are reflected in the activation of circumjacent pathways upon DNA damage
Source: Mol Oncol. 2022 Dec 2;17(1):82–97. doi: 10.1002/1878-0261.13337 (PMC9812841; doi:10.1002/1878-0261.13337)
Supplement: Supplementary file 10 — Table S3. List of NGS panel target genes focused on lymphoid malignancies. [file MOL2-17-82-s007.docx]

| *ARID1A* | *EP300* | *KRAS* | *POT1* |
| --- | --- | --- | --- |
| *ASXL1* | *EPOR* | *MEF2B* | *RB1* |
| *ATM* | *ETV6* | *MGA* | *RPS15* |
| *BIRC3* | *EZH2* | *MLL (KMT2A)* | *RUNX1* |
| *BRAF* | *FBXW7* | *MLL2 (KMT2D)* | *SAMHD1* |
| *BTG1* | *FIGNL1* | *MYC* | *SETD2* |
| *CARD11* | *FLT3* | *MYD88* | *SF3B1* |
| *CCND1* | *FOXO1* | *NF1* | *SH2B3* |
| *CD79A* | *HIST1H1E* | *NFKBIE* | *SHOX* |
| *CD79B* | *IKZF 1* | *NOTCH1* | *TNFRSF14* |
| *CDKN2A* | *IKZF 2* | *NOTCH2* | *TP53* |
| *CDKN2B* | *IKZF 3* | *NRAS* | *TYK2* |
| *CHD2* | *IL2RB* | *P2RY8* | *UBR5* |
| *CREBBP* | *IL3RA* | *PAG1* | *WHSC1 (NSD2)* |
| *CRLF2* | *IL7R* | *PAX5* | *XPO1* |
| *CSF2RA* | *JAK 1* | *PIM1* | *ZMYM3* |
| *EBF1* | *JAK 2* | *PTEN* |  |
| *EGR2* | *JAK 3* | *PTPN11* |  |

**Supplementary Table S3:** List of NGS panel target genes focused on lymphoid malignancies.
