# Supplementary material for: Distinct p53 phosphorylation patterns in chronic lymphocytic leukemia patients are reflected in the activation of circumjacent pathways upon DNA damage
Source: Mol Oncol. 2022 Dec 2;17(1):82–97. doi: 10.1002/1878-0261.13337 (PMC9812841; doi:10.1002/1878-0261.13337)
Supplement: Supplementary file 11 — Table S4. List of differentially expressed genes identified when untreated and doxorubicin‐treated conditions in paired samples within each experimental group were compared. [file MOL2-17-82-s001.docx]

**Supplementary Table S4:** List of differentially expressed genes identified when untreated and doxorubicin-treated conditions in paired samples within each experimental group were compared. Only those genes with adjusted *P*-value ≤ 0.05 and log2 fold change ≤ -1 or ≥ 1 are listed. Genes are listed based on significance. Upregulated genes are in green, while downregulated genes are in red.

| **Profile I (I/IV)** | | | |
| --- | --- | --- | --- |
| **log2FoldChange** | **adjusted p value** | **gene name** | **gene biotype** |
| -1,30 | 1,88E-19 | SUSD3 | protein_coding |
| -1,10 | 3,83E-15 | RHOBTB2 | protein_coding |
| -1,16 | 6,66E-15 | AC013394.1 | protein_coding |
| -1,04 | 1,57E-14 | OAS1 | protein_coding |
| -1,05 | 6,25E-14 | MEI1 | protein_coding |
| -1,41 | 2,22E-13 | S100A4 | protein_coding |
| -1,04 | 3,85E-13 | TYROBP | protein_coding |
| -1,53 | 1,63E-11 | LINC01252 | lincRNA |
| -1,24 | 4,36E-11 | AC027279.1 | sense_intronic |
| -1,19 | 5,30E-11 | CACFD1 | protein_coding |
| -1,10 | 1,05E-10 | MPEG1 | protein_coding |
| -1,23 | 2,64E-10 | KCNH2 | protein_coding |
| -1,02 | 1,42E-09 | GDF11 | protein_coding |
| -1,05 | 3,24E-09 | LILRA4 | protein_coding |
| -1,02 | 3,36E-09 | CD79A | protein_coding |
| -1,12 | 1,59E-08 | TSPOAP1 | protein_coding |
| -1,09 | 3,32E-08 | BAIAP3 | protein_coding |
| -1,34 | 4,54E-08 | TBXA2R | protein_coding |
| -1,06 | 5,07E-08 | H1FX | protein_coding |
| -1,42 | 5,75E-08 | CRIP2 | protein_coding |
| -1,09 | 9,48E-08 | KCNQ1 | protein_coding |
| -1,15 | 1,28E-07 | LEPR | protein_coding |
| -1,05 | 2,60E-07 | AC108718.1 | lincRNA |
| -1,29 | 7,00E-07 | RPL34P22 | processed_pseudogene |
| -1,08 | 1,19E-06 | AC011379.2 | processed_transcript |
| -1,06 | 1,28E-06 | TNFRSF17 | protein_coding |
| -1,03 | 2,19E-06 | AL034397.3 | antisense_RNA |
| -1,02 | 3,41E-06 | SFTPB | protein_coding |
| -1,21 | 5,10E-06 | RHPN1 | protein_coding |
| -1,02 | 5,24E-06 | VSIR | protein_coding |
| -1,01 | 7,56E-06 | ZNF853 | protein_coding |
| -1,04 | 8,10E-06 | AC027279.4 | TEC |
| -1,02 | 1,38E-05 | FPGT-TNNI3K | protein_coding |
| -1,06 | 3,44E-05 | HSPG2 | protein_coding |
| -1,02 | 5,63E-05 | FHIT | protein_coding |
| 2,27 | 1,08E-119 | MDM2 | protein_coding |
| 2,16 | 7,00E-83 | TNFRSF10B | protein_coding |
| 1,72 | 6,64E-67 | ZMAT3 | protein_coding |
| 2,89 | 1,06E-64 | FDXR | protein_coding |
| 1,57 | 1,67E-64 | RPS27L | protein_coding |
| 1,47 | 2,30E-63 | IER5 | protein_coding |
| **Profile I (II/IV)** | | | |
| **log2FoldChange** | **adjusted p value** | **gene name** | **gene biotype** |
| 1,54 | 2,98E-59 | TRIM22 | protein_coding |
| 2,00 | 1,37E-55 | AEN | protein_coding |
| 1,96 | 1,55E-54 | PCNA | protein_coding |
| 3,20 | 7,13E-53 | APOBEC3H | protein_coding |
| 1,54 | 8,94E-51 | NDUFAF6 | protein_coding |
| 1,60 | 3,15E-47 | DDB2 | protein_coding |
| 2,05 | 1,12E-43 | BBC3 | protein_coding |
| 2,53 | 6,37E-43 | PVT1 | lincRNA |
| 1,38 | 1,00E-42 | TMEM30A | protein_coding |
| 1,43 | 2,30E-42 | ASCC3 | protein_coding |
| 1,90 | 2,96E-41 | TNFSF9 | protein_coding |
| 1,19 | 4,72E-41 | NEAT1 | lincRNA |
| 1,72 | 8,26E-39 | SESN1 | protein_coding |
| 2,55 | 5,62E-38 | OXER1 | protein_coding |
| 2,69 | 2,02E-37 | EDA2R | protein_coding |
| 2,40 | 5,88E-37 | PLK2 | protein_coding |
| 2,61 | 1,96E-36 | ACTA2 | protein_coding |
| 2,71 | 5,42E-35 | HAAO | protein_coding |
| 1,18 | 1,16E-33 | RPS19 | protein_coding |
| 2,09 | 2,61E-33 | PHPT1 | protein_coding |
| 1,10 | 1,40E-32 | XPC | protein_coding |
| 1,48 | 2,26E-32 | PTP4A1 | protein_coding |
| 1,79 | 2,46E-32 | GADD45A | protein_coding |
| 1,68 | 6,29E-27 | LACC1 | protein_coding |
| 2,55 | 7,97E-27 | RNU1-106P | snRNA |
| 1,39 | 5,20E-26 | TRIAP1 | protein_coding |
| 1,04 | 1,30E-25 | DCP1B | protein_coding |
| 2,22 | 2,87E-25 | MIR34AHG | lincRNA |
| 2,45 | 3,11E-25 | LINC01759 | lincRNA |
| 1,27 | 1,04E-24 | HERC5 | protein_coding |
| 1,27 | 1,85E-24 | CREM | protein_coding |
| 2,19 | 2,38E-24 | CDKN1A | protein_coding |
| 2,27 | 5,54E-24 | PLXNB2 | protein_coding |
| 1,09 | 2,51E-23 | CEP57L1 | protein_coding |
| 1,79 | 5,20E-23 | TIGAR | protein_coding |
| 1,81 | 1,25E-22 | GLS2 | protein_coding |
| 1,61 | 6,55E-22 | FAS | protein_coding |
| 1,07 | 6,55E-22 | EI24 | protein_coding |
| 1,46 | 1,46E-21 | DRAM1 | protein_coding |
| 1,00 | 1,68E-20 | METTL7A | protein_coding |
| 1,28 | 5,11E-20 | AL158206.1 | sense_overlapping |
| 1,14 | 2,08E-19 | FBXO22 | protein_coding |
| 1,63 | 1,37E-18 | SESN2 | protein_coding |
| 1,10 | 6,54E-18 | TP53INP1 | protein_coding |
| 1,05 | 3,79E-17 | NDUFAF8 | protein_coding |
| **Profile I (III/IV)** | | | |
| **log2FoldChange** | **adjusted p value** | **gene name** | **gene biotype** |
| 1,34 | 1,14E-16 | NR4A3 | protein_coding |
| 1,12 | 1,14E-16 | TNFRSF10D | protein_coding |
| 1,63 | 1,92E-16 | SULF2 | protein_coding |
| 1,66 | 6,42E-16 | DDIT4 | protein_coding |
| 1,82 | 1,96E-15 | AL138781.1 | lincRNA |
| 2,20 | 2,57E-15 | PHLDA3 | protein_coding |
| 1,42 | 1,36E-14 | PRDM1 | protein_coding |
| 1,59 | 6,03E-14 | FOSL2 | protein_coding |
| 1,43 | 8,39E-14 | ATF3 | protein_coding |
| 1,26 | 1,51E-13 | BAX | protein_coding |
| 1,30 | 1,54E-13 | AC008105.1 | antisense_RNA |
| 1,22 | 1,56E-13 | TOP2A | protein_coding |
| 1,71 | 2,33E-13 | SNORA26 | snoRNA |
| 1,64 | 4,42E-13 | MGAT3 | protein_coding |
| 1,35 | 5,93E-13 | NBPF3 | protein_coding |
| 1,10 | 3,23E-12 | PRKY | transcribed_unprocessed_pseudogene |
| 1,19 | 3,46E-12 | DUSP4 | protein_coding |
| 1,48 | 3,60E-11 | DUSP5 | protein_coding |
| 1,62 | 5,30E-11 | KCNN3 | protein_coding |
| 1,22 | 8,72E-11 | SNHG3 | sense_intronic |
| 1,32 | 1,46E-10 | NUDT8 | protein_coding |
| 1,14 | 2,26E-10 | EFCAB5 | protein_coding |
| 1,02 | 7,47E-10 | CITED2 | protein_coding |
| 1,58 | 1,07E-09 | NALT1 | antisense_RNA |
| 1,23 | 1,15E-09 | AL139393.2 | antisense_RNA |
| 1,01 | 4,44E-09 | INAFM2 | protein_coding |
| 1,03 | 5,63E-09 | SGK1 | protein_coding |
| 1,03 | 5,95E-09 | HIST1H2AG | protein_coding |
| 1,21 | 1,21E-08 | CHI3L2 | protein_coding |
| 1,20 | 1,92E-08 | HIST1H4B | protein_coding |
| 1,54 | 2,91E-08 | JSRP1 | protein_coding |
| 1,26 | 4,18E-08 | RN7SKP118 | misc_RNA |
| 1,08 | 5,13E-08 | CD70 | protein_coding |
| 1,07 | 5,14E-08 | RPS19P7 | processed_pseudogene |
| 1,16 | 1,26E-07 | FAM212B | protein_coding |
| 1,38 | 1,72E-07 | AL109976.1 | lincRNA |
| 1,15 | 1,99E-07 | TP53I3 | protein_coding |
| 1,13 | 3,93E-07 | AL513523.10 | protein_coding |
| 1,20 | 6,66E-07 | HIST1H2BJ | protein_coding |
| 1,26 | 8,20E-07 | ASTN2 | protein_coding |
| 1,08 | 8,49E-07 | AL117336.2 | sense_intronic |
| 1,23 | 1,64E-06 | AC025423.1 | antisense_RNA |
| 1,20 | 1,85E-06 | LINC01619 | processed_transcript |
| 1,10 | 2,16E-06 | HIST1H2AH | protein_coding |
| 1,18 | 2,84E-06 | AL031666.1 | antisense_RNA |
| **Profile I (III/IV)** | | | |
| **log2FoldChange** | **adjusted p value** | **gene name** | **gene biotype** |
| 1,21 | 5,32E-06 | AL021807.1 | lincRNA |
| 1,03 | 6,52E-06 | LRRC32 | protein_coding |
| 1,05 | 8,94E-06 | AC007996.1 | sense_intronic |
| 1,15 | 9,76E-06 | AL157394.1 | sense_overlapping |
| 1,12 | 9,77E-06 | TYMS | protein_coding |
| 1,00 | 1,35E-05 | HIST1H4I | protein_coding |
| 1,28 | 1,80E-05 | PHLDA2 | protein_coding |
| 1,00 | 4,80E-05 | HIST1H2BH | protein_coding |
| 1,02 | 0,000105762 | HIST1H3C | protein_coding |
| 1,04 | 0,00010693 | VWCE | protein_coding |
| 1,07 | 0,000118334 | HIST2H3D | protein_coding |
| 1,03 | 0,000132238 | MELTF-AS1 | antisense_RNA |
| 1,02 | 0,000223051 | AL135905.2 | antisense_RNA |
| 1,06 | 0,000268136 | INSM1 | protein_coding |
| 1,07 | 0,000313183 | KLK4 | protein_coding |
| 1,02 | 0,000365454 | ADAMTS7 | protein_coding |
| 1,05 | 0,000588594 | AC104695.3 | sense_intronic |
|  | | | |
| **Profile II** | | | |
| No significantly differentially expressed genes were detected. | | | |
|  | | | |
| ***TP53* mutants** | | | |
| **log2FoldChange** | **adjusted p value** | **gene name** | **gene biotype** |
| -1,16 | 1,83E-06 | CCL4 | protein_coding |
| -1,02 | 3,50E-09 | PIGR | protein_coding |
| -1,02 | 1,36E-05 | SIGLEC14 | protein_coding |
