# Supplementary material for: Distinct p53 phosphorylation patterns in chronic lymphocytic leukemia patients are reflected in the activation of circumjacent pathways upon DNA damage
Source: Mol Oncol. 2022 Dec 2;17(1):82–97. doi: 10.1002/1878-0261.13337 (PMC9812841; doi:10.1002/1878-0261.13337)
Supplement: Supplementary file 12 — Table S5. List of progeny P values. [file MOL2-17-82-s011.docx]

**Supplementary Table S5:** List of Progeny *P* values. Significant *P* values (< 0.05) are in bold. If a pathway is significantly more active in the first group from the group comparison, the cell is filled with green, if it is less active, the cell is in red.

| **Controls** | | | |
| --- | --- | --- | --- |
| **Pathway** | **Profile I vs. II** | **Profile I vs. *TP53* mut** | **Profile II vs. *TP53* mut** |
| Androgen | 0.14 | 0.22 | 0.71 |
| EGFR | 0.24 | 0.75 | 0.1 |
| Estrogen | **0.013** | 0.74 | **0.069** |
| Hypoxia | **0.009** | **0.00087** | **0.0033** |
| JAK-STAT | 0.77 | 0.31 | 0.21 |
| MAPK | 0.14 | 0.75 | **0.034** |
| NFkB | 0.27 | **0.047** | 0.63 |
| p53 | 0.95 | 0.18 | 0.21 |
| PI3K | 0.59 | 0.21 | **0.031** |
| TGFb | **0.0031** | 0.24 | 0.05 |
| TNFa | 0.14 | **0.0038** | 0.23 |
| Trail | 0.6 | 0.32 | 0.24 |
| VEGF | 0.99 | 0.18 | 0.19 |
| WNT | 0.28 | 0.34 | 0.76 |
| **After doxorubicin** | | | |
| **Pathway** | **Profile I vs. II** | **Profile I vs. *TP53* mut** | **Profile II vs. *TP53* mut** |
| Androgen | 0.51 | 0.51 | 0.25 |
| EGFR | 0.88 | 0.18 | 0.14 |
| Estrogen | 0.071 | 0.95 | 0.13 |
| Hypoxia | **0.0053** | **0.003** | **0.0097** |
| JAK-STAT | 0.58 | 0.56 | 0.29 |
| MAPK | 0.65 | 0.13 | **0.036** |
| NFkB | 0.99 | 0.82 | 0.82 |
| p53 | **5.9e-05** | **2.1e-09** | **6.7e-06** |
| PI3K | 0.43 | 0.76 | 0.28 |
| TGFb | 0.43 | **0.023** | **0.028** |
| TNFa | 0.83 | 0.33 | 0.46 |
| Trail | 0.93 | 0.88 | 0.84 |
| VEGF | 0.17 | **0.0015** | **0.037** |
| WNT | 0.097 | 0.14 | 0.75 |
| **Control vs. doxorubicin** | | | |
| **Pathway** | **Profile I** | **Profile II** | ***TP53* mut** |
| Androgen | 0.33 | 0.8 | 0.79 |
| EGFR | 0.22 | 0.36 | 0.82 |
| Estrogen | 0.7 | 0.97 | 0.76 |
| Hypoxia | 0.91 | 0.98 | 0.7 |
| JAK-STAT | 0.57 | 0.83 | 0.99 |
| MAPK | 0.24 | 0.51 | 0.97 |
| NFkB | 0.19 | 0.82 | 0.3 |
| p53 | **1.7e-08** | **0.028** | 0.51 |
| PI3K | 0.28 | 0.93 | 0.084 |
| TGFb | **0.0055** | 0.93 | 0.39 |
| TNFa | 0.32 | 0.78 | 0.29 |
| Trail | 0.28 | 0.99 | 0.61 |
| VEGF | 0.84 | 0.99 | 0.91 |
| WNT | **0.028** | 0.32 | 0.33 |
