# Supplementary material for: Distinct p53 phosphorylation patterns in chronic lymphocytic leukemia patients are reflected in the activation of circumjacent pathways upon DNA damage
Source: Mol Oncol. 2022 Dec 2;17(1):82–97. doi: 10.1002/1878-0261.13337 (PMC9812841; doi:10.1002/1878-0261.13337)
Supplement: Supplementary file 14 — Table S7. List of variants detected by targeted NGS panel (LYNX). [file MOL2-17-82-s002.docx]

**Supplementary Table S7: List of variants detected by targeted NGS panel (LYNX).**

| **patient ID** | **gene_symbol** | **Chromosome** | **Start_Position** | **Variant_Classification** | **Reference_Allele** | **Variant Allele** | **HGVSc** | **HGVSp** | **Transcript_ID** | **VAF** |
| --- | --- | --- | --- | --- | --- | --- | --- | --- | --- | --- |
| 241 | KRAS | 12 | 25227348 | missense_variant | G | C | c.176C>G | p.Ala59Gly | ENST00000256078 | 0,053 |
| 241 | NOTCH1 | 9 | 136496196 | frameshift_variant | CAG | C | c.7541_7542del | p.Pro2514ArgfsTer4 | ENST00000277541 | 0,251 |
| 536 | ATM | 11 | 108295038 | missense_variant | G | A | c.4888G>A | p.Asp1630Asn | ENST00000278616 | 0,975 |
| 536 | NOTCH1 | 9 | 136495700 | 3_prime_UTR_variant | T | C | c.*371A>G | . | ENST00000277541 | 0,414 |
| 604 | BIRC3 | 11 | 102331153 | frameshift_variant | A | AT | c.1236_1237insT | p.Val413CysfsTer3 | ENST00000263464 | 0,185 |
| 636 | RPS15 | 19 | 1440424 | missense_variant | G | A | c.421G>A | p.Gly141Arg | ENST00000593052 | 0,073 |
| 912 | ATM | 11 | 108256267 | missense_variant | T | G | c.2177T>G | p.Leu726Arg | ENST00000278616 | 0,389 |
| 912 | ATM | 11 | 108329211 | missense_variant | T | G | c.7280T>G | p.Leu2427Arg | ENST00000278616 | 0,486 |
| 1003 | KRAS | 12 | 25245347 | missense_variant | C | T | c.38G>A | p.Gly13Asp | ENST00000256078 | 0,193 |
| 1085 | BIRC3 | 11 | 102331211 | frameshift_variant | A | AG | c.1295dup | p.Glu433ArgfsTer5 | ENST00000263464 | 0,107 |
| 1085 | BIRC3 | 11 | 102336925 | frameshift_variant | AC | A | c.1639del | p.Gln547AsnfsTer21 | ENST00000263464 | 0,184 |
| 1113 | NFKBIE | 6 | 44265001 | frameshift_variant | TGTAA | T | c.759_762del | p.Tyr254SerfsTer13 | ENST00000275015 | 0,444 |
| 1113 | NRAS | 1 | 114713909 | missense_variant | G | T | c.181C>A | p.Gln61Lys | ENST00000369535 | 0,39 |
| 1120 | ATM | 11 | 108317413 | missense_variant | A | G | c.6239A>G | p.Tyr2080Cys | ENST00000278616 | 0,983 |
| 1121 | ATM | 11 | 108343321 | stop_gained | A | T | c.8368A>T | p.Arg2790Ter | ENST00000278616 | 0,155 |
| 1141 | NFKBIE | 6 | 44265001 | frameshift_variant | TGTAA | T | c.759_762del | p.Tyr254SerfsTer13 | ENST00000275015 | 0,088 |
| 1141 | NRAS | 1 | 114713909 | missense_variant | G | T | c.181C>A | p.Gln61Lys | ENST00000369535 | 0,102 |
| 1191 | ATM | 11 | 108330247 | missense_variant | G | C | c.7341G>C | p.Leu2447Phe | ENST00000278616 | 0,349 |
| 1191 | NOTCH1 | 9 | 136496196 | frameshift_variant | CAG | C | c.7541_7542del | p.Pro2514ArgfsTer4 | ENST00000277541 | 0,422 |
| 1191 | SF3B1 | 2 | 197402110 | missense_variant | T | C | c.2098A>G | p.Lys700Glu | ENST00000335508 | 0,47 |
| 1200 | KRAS | 12 | 25225623 | missense_variant | C | G | c.441G>C | p.Lys147Asn | ENST00000256078 | 0,129 |
| 1200 | NFKBIE | 6 | 44265021 | frameshift_variant | TGCTGAGG | T | c.736_742del | p.Pro246SerfsTer20 | ENST00000275015 | 0,414 |
| 1200 | NOTCH1 | 9 | 136496196 | frameshift_variant | CAG | C | c.7541_7542del | p.Pro2514ArgfsTer4 | ENST00000277541 | 0,486 |
| 1203 | ATM | 11 | 108229218 | stop_gained | A | T | c.226A>T | p.Arg76Ter | ENST00000278616 | 0,98 |
| 1203 | RB1 | 13 | 48342598 | splice_acceptor_variant | G | A | c.265-1G>A | p.? | ENST00000267163 | 0,905 |
| 1218 | BRAF | 7 | 140781602 | missense_variant | C | G | c.1406G>C | p.Gly469Ala | ENST00000646891 | 0,459 |
| 1272 | SF3B1 | 2 | 197400753 | missense_variant | C | T | c.2680G>A | p.Asp894Asn | ENST00000335508 | 0,397 |
| 1274 | NOTCH1 | 9 | 136495693 | 3_prime_UTR_variant | T | C | c.*378A>G | . | ENST00000277541 | 0,074 |
| 1274 | NOTCH1 | 9 | 136496196 | frameshift_variant | CAG | C | c.7541_7542del | p.Pro2514ArgfsTer4 | ENST00000277541 | 0,452 |
| 1344 | SF3B1 | 2 | 197402110 | missense_variant | T | C | c.2098A>G | p.Lys700Glu | ENST00000335508 | 0,462 |
| 1358 | ATM | 11 | 108247128 | splice_donor_variant | G | C | c.1065+1G>C | p.? | ENST00000278616 | 0,947 |
| 1358 | BRAF | 7 | 140753393 | missense_variant&splice_region_variant | T | A | c.1742A>T | p.Asn581Ile | ENST00000646891 | 0,203 |
| 1358 | RPS15 | 19 | 1440459 | missense_variant | G | T | c.456G>T | p.Lys152Asn | ENST00000593052 | 0,459 |
| 1377 | RB1 | 13 | 48362859 | stop_gained | C | T | c.763C>T | p.Arg255Ter | ENST00000267163 | 0,442 |
| 1380 | NOTCH1 | 9 | 136496838 | frameshift_variant | A | AC | c.6900dup | p.Ser2301ValfsTer53 | ENST00000277541 | 0,989 |
| 1380 | SF3B1 | 2 | 197402636 | missense_variant | T | G | c.1997A>C | p.Lys666Thr | ENST00000335508 | 0,05 |
| 1412 | BRAF | 7 | 140753355 | missense_variant | C | T | c.1780G>A | p.Asp594Asn | ENST00000646891 | 0,478 |
| 1412 | NOTCH1 | 9 | 136496196 | frameshift_variant | CAG | C | c.7541_7542del | p.Pro2514ArgfsTer4 | ENST00000277541 | 0,5 |
| 1485 | BIRC3 | 11 | 102331193 | frameshift_variant | G | GA | c.1279dup | p.Ile427AsnfsTer11 | ENST00000263464 | 0,911 |
| 1485 | NOTCH1 | 9 | 136496196 | frameshift_variant | CAG | C | c.7541_7542del | p.Pro2514ArgfsTer4 | ENST00000277541 | 0,532 |
| 1524 | BRAF | 7 | 140753334 | missense_variant | T | C | c.1801A>G | p.Lys601Glu | ENST00000646891 | 0,318 |
| 1531 | RB1 | 13 | 48476758 | stop_gained | A | T | c.2578A>T | p.Lys860Ter | ENST00000267163 | 0,103 |
| 1531 | RB1 | 13 | 48476761 | missense_variant | A | G | c.2581A>G | p.Arg861Gly | ENST00000267163 | 0,103 |
| 1536 | SF3B1 | 2 | 197401887 | missense_variant&splice_region_variant | C | T | c.2225G>A | p.Gly742Asp | ENST00000335508 | 0,465 |
| 1546 | NOTCH1 | 9 | 136495700 | 3_prime_UTR_variant | T | C | c.*371A>G | . | ENST00000277541 | 0,45 |
| 1596 | SF3B1 | 2 | 197402097 | missense_variant | A | C | c.2111T>G | p.Ile704Ser | ENST00000335508 | 0,356 |
| 1751 | ATM | 11 | 108330303 | missense_variant | C | T | c.7397C>T | p.Ala2466Val | ENST00000278616 | 0,975 |
| 1751 | NFKBIE | 6 | 44260518 | frameshift_variant | G | GC | c.1129dup | p.Ala377GlyfsTer19 | ENST00000275015 | 0,055 |
| 1775 | ATM | 11 | 108335942 | missense_variant | T | C | c.8249T>C | p.Leu2750Ser | ENST00000278616 | 0,996 |
| 1775 | BIRC3 | 11 | 102331211 | frameshift_variant | A | AG | c.1295dup | p.Glu433ArgfsTer5 | ENST00000263464 | 0,29 |
| 1775 | SF3B1 | 2 | 197402098 | missense_variant | T | A | c.2110A>T | p.Ile704Phe | ENST00000335508 | 0,077 |
| 1843 | ATM | 11 | 108229193 | stop_gained | T | G | c.201T>G | p.Tyr67Ter | ENST00000278616 | 0,485 |
| 1843 | ATM | 11 | 108365383 | missense_variant | A | G | c.9046A>G | p.Lys3016Glu | ENST00000278616 | 0,493 |
| 1865 | ATM | 11 | 108316103 | missense_variant | G | A | c.6188G>A | p.Gly2063Glu | ENST00000278616 | 0,995 |
| 1869 | NFKBIE | 6 | 44265001 | frameshift_variant | TGTAA | T | c.759_762del | p.Tyr254SerfsTer13 | ENST00000275015 | 0,419 |
| 1869 | NFKBIE | 6 | 44265002 | stop_gained&frameshift_variant | G | GT | c.761dup | p.Tyr254Ter | ENST00000275015 | 0,357 |
| 1869 | NFKBIE | 6 | 44265129 | frameshift_variant | TAGGTGGA | T | c.628_634del | p.Ser210MetfsTer33 | ENST00000275015 | 0,173 |
| 1873 | NOTCH1 | 9 | 136496526 | stop_gained | G | A | c.7213C>T | p.Gln2405Ter | ENST00000277541 | 0,508 |
| 1931 | SF3B1 | 2 | 197402110 | missense_variant | T | C | c.2098A>G | p.Lys700Glu | ENST00000335508 | 0,509 |
| 1976 | ATM | 11 | 108297365 | missense_variant | G | T | c.4988G>T | p.Gly1663Val | ENST00000278616 | 0,99 |
| 2733 | ATM | 11 | 108249054 | missense_variant | T | A | c.1187T>A | p.Ile396Lys | ENST00000278616 | 0,985 |
