# Supplementary material for: Distinct p53 phosphorylation patterns in chronic lymphocytic leukemia patients are reflected in the activation of circumjacent pathways upon DNA damage
Source: Mol Oncol. 2022 Dec 2;17(1):82–97. doi: 10.1002/1878-0261.13337 (PMC9812841; doi:10.1002/1878-0261.13337)
Supplement: Supplementary file 15 — Table S8. List of validated somatic variants detected by whole‐exome sequencing. [file MOL2-17-82-s008.docx]

**Supplementary table S8: List of validated somatic variants detected by WES.**

Raw sequencing data were aligned to the human reference genome GRCh38. After variant calling and annotation, variants meeting the following criteria were filtered out:

i. classified as non-coding, ii. classified as synonymous without impact on splicing, iii. allele frequency in tumor less than 15%, iv. depth of coverage less than 20 in tumor and less than 10 in the germline, v. alternate allele depth less than 5, and vi. population frequency according to the GnomAD database more than 1%.

All remaining variants were inspected in the IGV viewer and only those validated as true positive were considered for the final analysis.

| **Patient ID** | **Gene_symbol** | **Chromosome** | **Start_Position** | **Variant_Classification** | **Reference_Allele** | **Variant Allele** | **HGVSc** | **HGVSp** | **Transcript_ID** | **VAF** |
| --- | --- | --- | --- | --- | --- | --- | --- | --- | --- | --- |
| 1980 | AAK1 | 2 | 69509361 | missense_variant | T | C | c.1876A>G | p.Lys626Glu | ENST00000409085 | 0,20 |
| 1980 | AL590132.1 | 1 | 210861169 | missense_variant | T | G | c.1278A>C | p.Glu426Asp | ENST00000639602 | 0,35 |
| 264 | ALK | 2 | 29328461 | missense_variant | T | A | c.1303A>T | p.Met435Leu | ENST00000389048 | 0,33 |
| 693 | ALKBH2 | 12 | 109088350 | stop_gained | G | C | c.443C>G | p.Ser148Ter | ENST00000440112 | 0,21 |
| 264 | ANKAR | 2 | 189743365 | missense_variant | A | G | c.3901A>G | p.Asn1301Asp | ENST00000520309 | 0,45 |
| 264 | ARHGEF40 | 14 | 21081770 | missense_variant | C | T | c.2902C>T | p.Arg968Trp | ENST00000298694 | 0,58 |
| 264 | ATP10B | 5 | 160632298 | missense_variant | G | A | c.1451C>T | p.Ser484Phe | ENST00000327245 | 0,44 |
| 1980 | C1orf56 | 1 | 151048582 | missense_variant | C | A | c.735C>A | p.His245Gln | ENST00000368926 | 0,46 |
| 636 | CABP1 | 12 | 120666895 | missense_variant | C | T | c.1108C>T | p.Arg370Cys | ENST00000316803 | 0,65 |
| 1980 | CHD2 | 15 | 92978331 | missense_variant | A | T | c.2675A>T | p.Gln892Leu | ENST00000394196 | 0,16 |
| 693 | CHKB | 22 | 50582256 | missense_variant | A | G | c.326T>C | p.Ile109Thr | ENST00000406938 | 0,16 |
| 1980 | CLCA2 | 1 | 86438922 | missense_variant | T | C | c.1019T>C | p.Met340Thr | ENST00000370565 | 0,18 |
| 264 | CNN2 | 19 | 1037641 | missense_variant | C | T | c.734C>T | p.Pro245Leu | ENST00000562958 | 0,20 |
| 1869 | CRP | 1 | 159713596 | missense_variant | C | T | c.604G>A | p.Val202Ile | ENST00000255030 | 0,18 |
| 1869 | DCAF1 | 3 | 51403329 | missense_variant | G | A | c.4279C>T | p.Leu1427Phe | ENST00000423656 | 0,34 |
| 1869 | DCN | 12 | 91153183 | missense_variant | G | T | c.659C>A | p.Pro220His | ENST00000052754 | 0,16 |
| 1980 | DICER1 | 14 | 95091302 | missense_variant | C | T | c.5428G>A | p.Asp1810Asn | ENST00000526495 | 0,20 |
| 264 | DNAH2 | 17 | 7740452 | missense_variant | T | A | c.1409T>A | p.Met470Lys | ENST00000572933 | 0,35 |
| 693 | EPYC | 12 | 90978160 | missense_variant | G | A | c.268C>T | p.Pro90Ser | ENST00000261172 | 0,18 |
| 264 | ETV3 | 1 | 157124947 | missense_variant | C | T | c.1433G>A | p.Arg478Gln | ENST00000368192 | 0,32 |
| 264 | FCHSD1 | 5 | 141651041 | missense_variant | G | A | c.98C>T | p.Ala33Val | ENST00000435817 | 0,26 |
| 636 | FRYL | 4 | 48582502 | missense_variant | T | C | c.1981A>G | p.Thr661Ala | ENST00000358350 | 0,44 |
| 264 | HGF | 7 | 81756067 | splice_region_variant&intron_variant | C | T | c.483-4G>A | p.? | ENST00000354224 | 0,44 |
| 264 | HMGN5 | X | 81114736 | missense_variant | T | A | c.762A>T | p.Glu254Asp | ENST00000358130 | 0,61 |
| 264 | HMMR | 5 | 163490419 | frameshift_variant | T | TA | c.2001dup | p.Gln668ThrfsTer3 | ENST00000393915 | 0,16 |
| 1980 | HOXA3 | 7 | 27110570 | missense_variant | A | C | c.71T>G | p.Phe24Cys | ENST00000612286 | 0,16 |
| 1869 | IFT140 | 16 | 1511060 | missense_variant | G | A | c.4273C>T | p.Arg1425Trp | ENST00000426508 | 0,46 |
| 264 | IGLL5 | 22 | 22888262 | splice_region_variant&intron_variant | A | T | c.206+3A>T | p.? | ENST00000526893 | 0,47 |
| 264 | IGLL5 | 22 | 22888260 | splice_donor_variant | G | A | c.206+1G>A | p.? | ENST00000526893 | 0,54 |
| 636 | INSYN2A | 10 | 127175301 | missense_variant | C | T | c.1095G>A | p.Met365Ile | ENST00000522781 | 0,56 |
| 1980 | KANK3 | 19 | 8335667 | missense_variant | C | G | c.160G>C | p.Glu54Gln | ENST00000330915 | 0,50 |
| 1869 | KDM7A | 7 | 140091924 | missense_variant | T | C | c.2611A>G | p.Ile871Val | ENST00000397560 | 0,45 |
| 693 | KIR3DL3 | 19 | 54725248 | inframe_deletion&splice_region_variant | GTTC | G | c.42_44del | p.Phe14del | ENST00000291860 | 0,40 |
| 636 | KLHL25 | 15 | 85768276 | missense_variant | T | A | c.1535A>T | p.Asp512Val | ENST00000337975 | 0,36 |
| 1869 | KRT18 | 12 | 52949184 | missense_variant | C | T | c.11C>T | p.Thr4Ile | ENST00000388837 | 0,25 |
| 1869 | KRT18 | 12 | 52949223 | missense_variant | G | A | c.50G>A | p.Gly17Asp | ENST00000388837 | 0,27 |
| 264 | LGR6 | 1 | 202318599 | missense_variant | ; | A | c.2296G>A | p.Val766Met | ENST00000367278 | 0,27 |
| 1869 | LOC105374299 | 3 | 195817428 | missense_variant | G | A | c.1714G>A | p.Gly572Ser | XM_011513362.2 | 0,30 |
| 1980 | LOC107984590 | 13 | 113011787 | missense_variant | T | C | c.1325A>G | p.Gln442Arg | XM_017020903.1 | 0,42 |
| 264 | LOC107985433 | 20 | 30287394 | missense_variant | C | A | c.314G>T | p.Cys105Phe | XM_017028192.1 | 0,33 |
| 264 | LOXL2 | 8 | 23298922 | missense_variant | A | T | c.2159T>A | p.Val720Asp | ENST00000389131 | 0,22 |
| 264 | LRFN5 | 14 | 41891515 | missense_variant | C | T | c.1651C>T | p.Arg551Trp | ENST00000298119 | 0,51 |
| 264 | LRP1B | 2 | 141247344 | missense_variant | T | A | c.474A>T | p.Glu158Asp | ENST00000389484 | 0,48 |
| 1980 | LRRC8D | 1 | 89933740 | missense_variant | C | G | c.672C>G | p.Asn224Lys | ENST00000337338 | 0,60 |
| 636 | MED12 | X | 71119403 | missense_variant | G | A | c.130G>A | p.Gly44Ser | ENST00000374080 | 0,24 |
| 636 | MED12 | X | 71119357 | splice_acceptor_variant | CGCTTTCCTGCCTCAGGATGAACTGACGGC | TT | c.100-16_113delinsTT | p.? | ENST00000374080 | 0,77 |
| 1869 | MED12 | X | 71119380 | missense_variant | T | G | c.107T>G | p.Leu36Arg | ENST00000374080 | 0,50 |
| 264 | MED12L | 3 | 151430325 | inframe_deletion | CCAG | C | c.6343_6345del | p.Gln2115del | ENST00000474524 | 0,15 |
| 636 | MMS22L | 6 | 97229201 | missense_variant | G | A | c.1732C>T | p.Leu578Phe | ENST00000275053 | 0,49 |
| 636 | MRPS5 | 2 | 95108249 | missense_variant | T | C | c.563A>G | p.Lys188Arg | ENST00000272418 | 0,48 |
| 636 | MT-CO3 | M | 9247 | missense_variant | G | A | c.41G>A | p.Ser14Asn | ENST00000362079 | 0,17 |
| 1869 | MUC5AC | 11 | 1190453 | missense_variant | C | T | c.12308C>T | p.Thr4103Ile | ENST00000621226 | 0,15 |
| 1869 | MUC5AC | 11 | 1190429 | missense_variant | C | G | c.12284C>G | p.Thr4095Ser | ENST00000621226 | 0,19 |
| 264 | MYD88 | 3 | 38141150 | stop_lost | T | C | c.574T>C | p.Ter192ArgextTer8 | ENST00000443433 | 0,50 |
| 636 | MYH2 | 17 | 10525019 | missense_variant | T | C | c.4709A>G | p.Glu1570Gly | ENST00000397183 | 0,19 |
| 1869 | MYO9B | 19 | 17200777 | missense_variant | A | G | c.4511A>G | p.Gln1504Arg | ENST00000595618 | 0,53 |
| 1980 | MYT1L | 2 | 1892152 | missense_variant | G | A | c.2168C>T | p.Thr723Met | ENST00000647738 | 0,48 |
| 1869 | NFKBIE | 6 | 44265002 | stop_gained&frameshift_variant | G | GT | c.761dup | p.Tyr254Ter | ENST00000275015 | 0,24 |
| 1869 | NFKBIE | 6 | 44265129 | frameshift_variant | TAGGTGGA | T | c.628_634del | p.Ser210MetfsTer33 | ENST00000275015 | 0,26 |
| 1869 | NFKBIE | 6 | 44265001 | frameshift_variant | TGTAA | T | c.759_762del | p.Tyr254SerfsTer13 | ENST00000275015 | 0,38 |
| 636 | NYNRIN | 14 | 24416709 | frameshift_variant | T | TAC | c.4969_4970dup | p.Ala1658ArgfsTer19 | ENST00000382554 | 0,50 |
| 1869 | OR10K2 | 1 | 158420226 | missense_variant | A | T | c.641T>A | p.Ile214Asn | ENST00000641042 | 0,21 |
| 636 | OR2A12 | 7 | 144095843 | missense_variant | G | A | c.736G>A | p.Val246Met | ENST00000641592 | 0,45 |
| 636 | OR8U1 | 11 | 56375745 | missense_variant | G | T | c.122G>T | p.Gly41Val | ENST00000302270 | 0,48 |
| 693 | PAX4 | 7 | 127610907 | missense_variant | G | A | c.992C>T | p.Ala331Val | ENST00000378740 | 0,43 |
| 264 | PCDHA6 | 5 | 140828192 | missense_variant | C | A | c.101C>A | p.Ser34Tyr | ENST00000529310 | 0,46 |
| 264 | PCLO | 7 | 83154752 | missense_variant | G | A | c.1889C>T | p.Thr630Met | ENST00000333891 | 0,33 |
| 1869 | PDE9A | 21 | 42760889 | missense_variant | A | G | c.1067A>G | p.His356Arg | ENST00000291539 | 0,47 |
| 1869 | PGM2L1 | 11 | 74343347 | missense_variant | G | T | c.1288C>A | p.Leu430Ile | ENST00000298198 | 0,43 |
| 693 | PID1 | 2 | 229262884 | missense_variant | C | G | c.93G>C | p.Leu31Phe | XM_017004404.1 | 0,47 |
| 1980 | PID1 | 2 | 229025699 | missense_variant | T | G | c.686A>C | p.Asp229Ala | ENST00000354069 | 0,50 |
| 264 | PKHD1L1 | 8 | 109490020 | missense_variant | C | T | c.9949C>T | p.Pro3317Ser | ENST00000378402 | 0,32 |
| 693 | POLR2A | 17 | 7508417 | missense_variant | C | T | c.3407C>T | p.Thr1136Ile | NM_000937.5 | 0,18 |
| 1869 | PRSS2 | 7 | 142772607 | splice_region_variant&intron_variant | A | G | c.242+4A>G | p.? | ENST00000633969 | 0,68 |
| 1869 | PTPN13 | 4 | 86750770 | missense_variant | A | G | c.2951A>G | p.Asn984Ser | ENST00000436978 | 0,16 |
| 636 | RBM39 | 20 | 35732104 | inframe_deletion | GACT | G | c.130_132del | p.Ser44del | ENST00000253363 | 0,34 |
| 264 | RNF34 | 12 | 121430237 | missense_variant | A | C | c.1267A>C | p.Lys423Gln | ENST00000392464 | 0,24 |
| 1980 | RP1L1 | 8 | 10609282 | missense_variant | G | A | c.4816C>T | p.Arg1606Cys | ENST00000382483 | 0,45 |
| 1869 | SALL1 | 16 | 51137521 | missense_variant | G | T | c.3566C>A | p.Thr1189Asn | ENST00000251020 | 0,21 |
| 1869 | SCARA3 | 8 | 27659222 | missense_variant | C | T | c.1052C>T | p.Ala351Val | ENST00000301904 | 0,52 |
| 264 | SEC24A | 5 | 134649149 | missense_variant | G | A | c.73G>A | p.Ala25Thr | ENST00000398844 | 0,19 |
| 1869 | SLC23A2 | 20 | 4869922 | frameshift_variant | T | TG | c.1233dup | p.Ile412HisfsTer18 | ENST00000379333 | 0,61 |
| 693 | SLC23A3 | 2 | 219167981 | stop_gained | G | A | c.886C>T | p.Gln296Ter | ENST00000455516 | 0,19 |
| 693 | SSTR1 | 14 | 38209970 | missense_variant | T | A | c.581T>A | p.Val194Asp | ENST00000267377 | 0,25 |
| 1980 | TAS2R19 | 12 | 11021703 | missense_variant | A | G | c.869T>C | p.Phe290Ser | ENST00000390673 | 0,17 |
| 1869 | TBX21 | 17 | 47744831 | frameshift_variant | TC | T | c.1075del | p.Leu359TyrfsTer46 | ENST00000177694 | 0,18 |
| 264 | TECPR1 | 7 | 98229137 | missense_variant | C | T | c.2312G>A | p.Arg771Gln | ENST00000447648 | 0,50 |
| 693 | TMC6 | 17 | 78120677 | missense_variant | G | A | c.1691C>T | p.Thr564Met | ENST00000590602 | 0,17 |
| 1869 | TNK2 | 3 | 195868364 | missense_variant | G | A | c.2123C>T | p.Pro708Leu | ENST00000381916 | 0,50 |
| 1980 | TRIM64B | 11 | 89870749 | missense_variant | C | T | c.1222G>A | p.Val408Met | ENST00000329862 | 0,30 |
| 693 | TRIM7 | 5 | 181205095 | missense_variant | G | A | c.16C>T | p.Pro6Ser | ENST00000274773 | 0,27 |
| 264 | TTYH3 | 7 | 2656134 | missense_variant | G | T | c.1063G>T | p.Val355Leu | ENST00000258796 | 0,49 |
| 264 | ULK4 | 3 | 41754466 | missense_variant | C | T | c.2216G>A | p.Arg739His | ENST00000301831 | 0,36 |
| 264 | WASF3 | 13 | 26681319 | missense_variant&splice_region_variant | G | A | c.982G>A | p.Gly328Arg | ENST00000335327 | 0,50 |
| 1980 | WDR27 | 6 | 169602218 | splice_donor_variant | C | T | c.2424+1G>A | p.? | ENST00000448612 | 0,37 |
| 693 | XPO1 | 2 | 61492192 | missense_variant | T | C | c.1730A>G | p.His577Arg | ENST00000401558 | 0,29 |
| 1869 | XPO4 | 13 | 20822283 | stop_gained | G | A | c.847C>T | p.Arg283Ter | ENST00000255305 | 0,44 |
| 1869 | YIPF6 | X | 68531943 | missense_variant | G | A | c.655G>A | p.Val219Ile | ENST00000462683 | 0,41 |
