# Supplementary material for: Distinct p53 phosphorylation patterns in chronic lymphocytic leukemia patients are reflected in the activation of circumjacent pathways upon DNA damage
Source: Mol Oncol. 2022 Dec 2;17(1):82–97. doi: 10.1002/1878-0261.13337 (PMC9812841; doi:10.1002/1878-0261.13337)
Supplement: Supplementary file 16 — Data S1. Supplementary material and legends. [file MOL2-17-82-s003.pdf]

Supplementary Table S1: Overview of samples carrying *TP53* aberrations. VAF variant allele frequency. * Biallelic inactivation of *TP53* gene by the mechanism of copy-neutral loss-of-heterozygosity.

Supplementary Table S2: Antibodies used in the study.

Supplementary Table S3: List of NGS panel target genes focused on lymphoid malignancies.

Supplementary Table S4: List of differentially expressed genes identified when untreated and doxorubicin-treated conditions in paired samples within each experimental group were compared. Only those genes with adjusted *P*-value ≤ 0.05 and log2 fold change ≤ -1 or ≥ 1 are listed. Genes are listed based on significance. Upregulated genes are in green, while downregulated genes are in red.

Supplementary Table S5: List of Progeny *P* values. Significant *P* values (< 0.05) are in bold. If a pathway is significantly more active in the first group from the group comparison, the cell is filled with green, if it is less active, the cell is in red.

Supplementary Table S6: List of hypoxia-related genes used in PROGENy analysis.

Supplementary Table S7: List of variants detected by targeted NGS panel (LYNX).

Supplementary Table S8: List of validated somatic variants detected by WES. Raw sequencing data were aligned to the human reference genome GRCh38. After variant calling and annotation, variants meeting the following criteria were filtered out:

i. classified as non-coding,
ii. classified as synonymous without impact on splicing,
iii. allele frequency in tumor less than 15%,
iv. depth of coverage less than 20 in tumor and less than 10 in the germline,
v. alternate allele depth less than 5,
vi. population frequency according to the GnomAD database more than 1%.

All remaining variants were inspected in the IGV viewer and only those validated as true positive were considered for the final analysis.

Supplementary Figure S1: Phosphorylation patterns detected by Zn(II) Phos-Tag technique (line p53 phospho highlighted in red). A.1-2. profile I after doxorubicine treatment. B.1-2. profile II after doxorubicine treatment. no tr =no treatment, doxo = doxorubicin, flud = fludarabine. -,+ with or without phosphatase treatment, respectively. X marks conditions that were not run.

Supplementary Figure S2: Western blot analysis of basal p53 protein levels. BRNO1215 *TP53* mutated sample was used as a positive contol with a strong p53 induction without any treatment. P1 and P2 refers to profile I and profile II sample groups, respectively. Actin was used as a loading control.

Supplementary Figure S3: Relative miR34-a expression levels in uncultured primary CLL cells were established by qRT-PCR (using ΔC_t method). Median is depicted with line. Statistical analyses were done using Mann-Whitney test. *P*-value < 0.001 is marked as ***.

Supplementary Figure S4: qRT-PCR of p53 targets after fludarabine treatment. Expression of *BAX*, *BBC3*, *CDKN1A* and *GADD45A* was calculated relative to mean of two house keeping genes using $\Delta\Delta C_t$ method. Statistical analyses were done by using Mann-Whitney test. *P*-values < 0.05 are coded as *, those < 0.01 as ** and < 0.001 as *** or stated by the exact number. Dashed line at y=1 depicts no induction. Horizontal lines depict medians of each experimental group.

Supplementary Figure S5: Activity of hypoxia pathway and HIF1A transcription factor. **A.** PROGENy analysis uncovered that activity of the hypoxia pathway significantly differed among the experimental groups. Genes, which were used to calculate the activity of this pathway, are shown. Individual genes and their weight (x axis) are depicted in function of t statistics (y axis, test statistics from the comparison of gene expression between respective groups). **B.** DoRothEA, calculating the activity of individual transcription factors through looking at the expression patterns of their targets, was applied to study the activity of HIF1A. Volcano plots depict target genes, which were used to calculate HIF1A activity. Statistics from the individual comparisons are in the table bellow; a negative statistics means lower activity of the first group of the respective comparison.

Supplementary Figure S6: Patients' clinical outcome in relation to phospho-profiles. **A.** A sub-cohort of patients uniformly treated by the chemoimmunotherapy regimen fludarabine+chlorambuci+rituximab (FCR; N=20) was analyzed for the time to second treatment (TTST). **B.** For overall survival (OS), patients were stratified as to whether they received targeted inhibitor treatment at any time during the course of the disease. No difference between profile I and profile II was observed.

Supplementary Figure S7: H2AX phosphorylation. Representative samples from profile I (N = 4) and profile II (N = 8) were cultured for 30 min (left panel) or 24 h (right panel) in vitro with or without doxorubicin. Afterwards, cells were collected, fixed, permeabilized, stained for H2AX (Ser139), and measured by flow cytometry. Cells with biallelic defects in ATM are depicted with filled symbols. Bars represent the mean values. No significant differences were found between profile I and II samples (Mann-Whitney test for profile comparisons, Wilcoxon signed rank test for paired comparisons between untreated and treated paired samples).

Supplementary Table S1.

ID	*TP53* mutation			del 17p (FISH)	
	**variant in c.DNA (LRG_321t1)**	**variant in protein (LRG_321p1)**	**cumulative VAF (%)**	**yes/no**	**frequency (%)**
1215	c.830G>T	p.Cys277Phe	98.9	No*	0
2161	c.919+1G>T; c.673-2A>G	p.?; p.?	99.0	Yes	93
271	c.797G>A	p.Gly266Glu	99.5	No*	0
2496	c.783-1G>T	p.?	99.7	No*	0
672	c.321C>A	p.Tyr107Ter	99.4	Yes	86
1727	c.741_742delinsTT	p.Arg248Trp	93.8	Yes	87
2010	c.569C>T; c.672+1G>T	p.Pro190Leu; p.?	97.3	Yes	77
2532	c.559+5G>A; c.626_627del	p.?; p.Arg209LysfsTer	87.5	Yes	85
2538	c.742C>T; c.287del	p.Arg248Trp; p.Ser96LeufsTer	94.9	Yes	87

Supplementary Table S2.

**Target**	**Distributor**	**Distributor’s catalogue number**
p53 [DO-1]	abcam	#ab204452
β-actin	Sigma-Aldrich	#A5441
Phospho-p53 (Ser6)	Cell Signaling	#9285
Phospho-p53 (Ser9)	Cell Signaling	#9288
Phospho-p53 (Ser15)	Cell Signaling	#9284
Phospho-p53 (Ser20)	Cell Signaling	#9287
Phospho-p53 (Ser20)	Cell Signaling	#9287
Phospho-p53 (Ser46)	Cell Signaling	#2521
Phospho-p53 (Thr81)	Cell Signaling	#2676
Phospho-p53 (Ser315)	Cell Signaling	#2528
Phospho-p53 (Ser392) 9281	Cell Signaling	#9281
Goat Anti-Rabbit IgG (H + L)-HRP Conjugate	Bio-Rad	#1706515
Goat Anti-Mouse IgG (H + L)-HRP Conjugate	Bio-Rad	#1706516

Supplementary Table S3.

ARID1A	*EP300*	*KRAS*	*POT1*
ASXL1	*EPOR*	*MEF2B*	*RB1*
ATM	*ETV6*	*MGA*	*RPS15*
BIRC3	*EZH2*	*MLL (KMT2A)*	*RUNX1*
BRAF	*FBXW7*	*MLL2 (KMT2D)*	*SAMHD1*
BTG1	*FIGNL1*	*MYC*	*SETD2*
CARD11	*FLT3*	*MYD88*	*SF3B1*
CCND1	*FOXO1*	*NF1*	*SH2B3*
CD79A	*HIST1H1E*	*NFKBIE*	*SHOX*
CD79B	*IKZF 1*	*NOTCH1*	*TNFRSF14*
CDKN2A	*IKZF 2*	*NOTCH2*	*TP53*
CDKN2B	*IKZF 3*	*NRAS*	*TYK2*
CHD2	*IL2RB*	*P2RY8*	*UBR5*
CREBBP	*IL3RA*	*PAG1*	*WHSC1 (NSD2)*
CRLF2	*IL7R*	*PAX5*	*XPO1*
CSF2RA	*JAK 1*	*PIM1*	*ZMYM3*
EBF1	*JAK 2*	*PTEN*	
EGR2	*JAK 3*	*PTPN11*	

Supplementary Table S4.

Profile I (I/IV)			
**log2FoldChange**	**adjusted p value**	**gene name**	**gene biotype**
-1,30	1,88E-19	SUSD3	protein_coding
-1,10	3,83E-15	RHOBTB2	protein_coding
-1,16	6,66E-15	AC013394.1	protein_coding
-1,04	1,57E-14	OAS1	protein_coding
-1,05	6,25E-14	MEI1	protein_coding
-1,41	2,22E-13	S100A4	protein_coding
-1,04	3,85E-13	TYROBP	protein_coding
-1,53	1,63E-11	LINC01252	lincRNA
-1,24	4,36E-11	AC027279.1	sense_intronic
-1,19	5,30E-11	CACFD1	protein_coding
-1,10	1,05E-10	MPEG1	protein_coding
-1,23	2,64E-10	KCNH2	protein_coding
-1,02	1,42E-09	GDF11	protein_coding
-1,05	3,24E-09	LILRA4	protein_coding
-1,02	3,36E-09	CD79A	protein_coding
-1,12	1,59E-08	TSPOAP1	protein_coding
-1,09	3,32E-08	BAIAP3	protein_coding
-1,34	4,54E-08	TBXA2R	protein_coding
-1,06	5,07E-08	H1FX	protein_coding
-1,42	5,75E-08	CRIP2	protein_coding
-1,09	9,48E-08	KCNQ1	protein_coding
-1,15	1,28E-07	LEPR	protein_coding
-1,05	2,60E-07	AC108718.1	lincRNA
-1,29	7,00E-07	RPL34P22	processed_pseudogene
-1,08	1,19E-06	AC011379.2	processed_transcript
-1,06	1,28E-06	TNFRSF17	protein_coding
-1,03	2,19E-06	AL034397.3	antisense_RNA
-1,02	3,41E-06	SFTPB	protein_coding
-1,21	5,10E-06	RHPN1	protein_coding
-1,02	5,24E-06	VSIR	protein_coding
-1,01	7,56E-06	ZNF853	protein_coding
-1,04	8,10E-06	AC027279.4	TEC
-1,02	1,38E-05	FPGT-TNNI3K	protein_coding
-1,06	3,44E-05	HSPG2	protein_coding
-1,02	5,63E-05	FHIT	protein_coding
2,27	1,08E-119	MDM2	protein_coding
2,16	7,00E-83	TNFRSF10B	protein_coding
1,72	6,64E-67	ZMAT3	protein_coding
2,89	1,06E-64	FDXR	protein_coding
1,57	1,67E-64	RPS27L	protein_coding
1,47	2,30E-63	IER5	protein_coding
**Profile I (II/IV)**			
**log2FoldChange**	**adjusted p value**	**gene name**	**gene biotype**
1,54	2,98E-59	TRIM22	protein_coding

2,00	1,37E-55	AEN	protein_coding
1,96	1,55E-54	PCNA	protein_coding
3,20	7,13E-53	APOBEC3H	protein_coding
1,54	8,94E-51	NDUFAF6	protein_coding
1,60	3,15E-47	DDB2	protein_coding
2,05	1,12E-43	BBC3	protein_coding
2,53	6,37E-43	PVT1	lincRNA
1,38	1,00E-42	TMEM30A	protein_coding
1,43	2,30E-42	ASCC3	protein_coding
1,90	2,96E-41	TNFSF9	protein_coding
1,19	4,72E-41	NEAT1	lincRNA
1,72	8,26E-39	SESN1	protein_coding
2,55	5,62E-38	OXER1	protein_coding
2,69	2,02E-37	EDA2R	protein_coding
2,40	5,88E-37	PLK2	protein_coding
2,61	1,96E-36	ACTA2	protein_coding
2,71	5,42E-35	HAAO	protein_coding
1,18	1,16E-33	RPS19	protein_coding
2,09	2,61E-33	PHPT1	protein_coding
1,10	1,40E-32	XPC	protein_coding
1,48	2,26E-32	PTP4A1	protein_coding
1,79	2,46E-32	GADD45A	protein_coding
1,68	6,29E-27	LACC1	protein_coding
2,55	7,97E-27	RNU1-106P	snRNA
1,39	5,20E-26	TRIAP1	protein_coding
1,04	1,30E-25	DCP1B	protein_coding
2,22	2,87E-25	MIR34AHG	lincRNA
2,45	3,11E-25	LINC01759	lincRNA
1,27	1,04E-24	HERC5	protein_coding
1,27	1,85E-24	CREM	protein_coding
2,19	2,38E-24	CDKN1A	protein_coding
2,27	5,54E-24	PLXNB2	protein_coding
1,09	2,51E-23	CEP57L1	protein_coding
1,79	5,20E-23	TIGAR	protein_coding
1,81	1,25E-22	GLS2	protein_coding
1,61	6,55E-22	FAS	protein_coding
1,07	6,55E-22	EI24	protein_coding
1,46	1,46E-21	DRAM1	protein_coding
1,00	1,68E-20	METTL7A	protein_coding
1,28	5,11E-20	AL158206.1	sense_overlapping
1,14	2,08E-19	FBXO22	protein_coding
1,63	1,37E-18	SESN2	protein_coding
1,10	6,54E-18	TP53INP1	protein_coding
1,05	3,79E-17	NDUFAF8	protein_coding
**Profile I (III/IV)**			
**log2FoldChange**	**adjusted p value**	**gene name**	**gene biotype**
1,34	1,14E-16	NR4A3	protein_coding

1,12	1,14E-16	TNFRSF10D	protein_coding
1,63	1,92E-16	SULF2	protein_coding
1,66	6,42E-16	DDIT4	protein_coding
1,82	1,96E-15	AL138781.1	lincRNA
2,20	2,57E-15	PHLDA3	protein_coding
1,42	1,36E-14	PRDM1	protein_coding
1,59	6,03E-14	FOSL2	protein_coding
1,43	8,39E-14	ATF3	protein_coding
1,26	1,51E-13	BAX	protein_coding
1,30	1,54E-13	AC008105.1	antisense_RNA
1,22	1,56E-13	TOP2A	protein_coding
1,71	2,33E-13	SNORA26	snoRNA
1,64	4,42E-13	MGAT3	protein_coding
1,35	5,93E-13	NBPF3	protein_coding
1,10	3,23E-12	PRKY	transcribed_unprocessed_pseudogene
1,19	3,46E-12	DUSP4	protein_coding
1,48	3,60E-11	DUSP5	protein_coding
1,62	5,30E-11	KCNN3	protein_coding
1,22	8,72E-11	SNHG3	sense_intronic
1,32	1,46E-10	NUDT8	protein_coding
1,14	2,26E-10	EFCAB5	protein_coding
1,02	7,47E-10	CITED2	protein_coding
1,58	1,07E-09	NALT1	antisense_RNA
1,23	1,15E-09	AL139393.2	antisense_RNA
1,01	4,44E-09	INAFM2	protein_coding
1,03	5,63E-09	SGK1	protein_coding
1,03	5,95E-09	HIST1H2AG	protein_coding
1,21	1,21E-08	CHI3L2	protein_coding
1,20	1,92E-08	HIST1H4B	protein_coding
1,54	2,91E-08	JSRP1	protein_coding
1,26	4,18E-08	RN7SKP118	misc_RNA
1,08	5,13E-08	CD70	protein_coding
1,07	5,14E-08	RPS19P7	processed_pseudogene
1,16	1,26E-07	FAM212B	protein_coding
1,38	1,72E-07	AL109976.1	lincRNA
1,15	1,99E-07	TP53I3	protein_coding
1,13	3,93E-07	AL513523.10	protein_coding
1,20	6,66E-07	HIST1H2BJ	protein_coding
1,26	8,20E-07	ASTN2	protein_coding
1,08	8,49E-07	AL117336.2	sense_intronic
1,23	1,64E-06	AC025423.1	antisense_RNA
1,20	1,85E-06	LINC01619	processed_transcript
1,10	2,16E-06	HIST1H2AH	protein_coding
1,18	2,84E-06	AL031666.1	antisense_RNA
**Profile I (III/IV)**			
**log2FoldChange**	**adjusted p value**	**gene name**	**gene biotype**
1,21	5,32E-06	AL021807.1	lincRNA

1,03	6,52E-06	LRRC32	protein_coding
1,05	8,94E-06	AC007996.1	sense_intronic
1,15	9,76E-06	AL157394.1	sense_overlapping
1,12	9,77E-06	TYMS	protein_coding
1,00	1,35E-05	HIST1H4I	protein_coding
1,28	1,80E-05	PHLDA2	protein_coding
1,00	4,80E-05	HIST1H2BH	protein_coding
1,02	0,000105762	HIST1H3C	protein_coding
1,04	0,00010693	VWCE	protein_coding
1,07	0,000118334	HIST2H3D	protein_coding
1,03	0,000132238	MELTF-AS1	antisense_RNA
1,02	0,000223051	AL135905.2	antisense_RNA
1,06	0,000268136	INSM1	protein_coding
1,07	0,000313183	KLK4	protein_coding
1,02	0,000365454	ADAMTS7	protein_coding
1,05	0,000588594	AC104695.3	sense_intronic
**Profile II**			
No significantly differentially expressed genes were detected.			
***TP53* mutants**			
**log2FoldChange**	**adjusted p value**	**gene name**	**gene biotype**
-1,16	1,83E-06	CCL4	protein_coding
-1,02	3,50E-09	PIGR	protein_coding
-1,02	1,36E-05	SIGLEC14	protein_coding

Supplementary Table S5.

**Controls**			
**Pathway**	**Profile I vs. II**	**Profile I vs. *TP53* mut**	**Profile II vs. *TP53* mut**
Androgen	0.14	0.22	0.71
EGFR	0.24	0.75	0.1
Estrogen	**0.013**	0.74	**0.069**
Hypoxia	**0.009**	**0.00087**	**0.0033**
JAK-STAT	0.77	0.31	0.21
MAPK	0.14	0.75	**0.034**
NFkB	0.27	**0.047**	0.63
p53	0.95	0.18	0.21
PI3K	0.59	0.21	**0.031**
TGFb	**0.0031**	0.24	0.05
TNFa	0.14	**0.0038**	0.23
Trail	0.6	0.32	0.24
VEGF	0.99	0.18	0.19
WNT	0.28	0.34	0.76
**After doxorubicin**			
**Pathway**	**Profile I vs. II**	**Profile I vs. *TP53* mut**	**Profile II vs. *TP53* mut**
Androgen	0.51	0.51	0.25
EGFR	0.88	0.18	0.14
Estrogen	0.071	0.95	0.13
Hypoxia	**0.0053**	**0.003**	**0.0097**
JAK-STAT	0.58	0.56	0.29
MAPK	0.65	0.13	**0.036**
NFkB	0.99	0.82	0.82
p53	**5.9e-05**	**2.1e-09**	**6.7e-06**
PI3K	0.43	0.76	0.28
TGFb	0.43	**0.023**	**0.028**
TNFa	0.83	0.33	0.46
Trail	0.93	0.88	0.84
VEGF	0.17	**0.0015**	**0.037**
WNT	0.097	0.14	0.75
**Control vs. doxorubicin**			
**Pathway**	**Profile I**	**Profile II**	***TP53* mut**
Androgen	0.33	0.8	0.79
EGFR	0.22	0.36	0.82
Estrogen	0.7	0.97	0.76
Hypoxia	0.91	0.98	0.7
JAK-STAT	0.57	0.83	0.99
MAPK	0.24	0.51	0.97
NFkB	0.19	0.82	0.3
p53	**1.7e-08**	**0.028**	0.51
PI3K	0.28	0.93	0.084
TGFb	**0.0055**	0.93	0.39
TNFa	0.32	0.78	0.29
Trail	0.28	0.99	0.61
VEGF	0.84	0.99	0.91
WNT	**0.028**	0.32	0.33

Supplementary table S6.

	weight (Hypoxia)	t statistics_p1_vs_mut	t statistics_p2_vs_mut	t statistics_p1_vs_p2
ADM	17,83	5,38	3,72	2,73
ALDOA	7,65	4,18	3,79	0,11
ALDOC	13,16	8,78	8,81	1,10
ANKRD37	8,48	6,51	5,83	1,14
ANKZF1	7,36	0,84	1,21	-0,68
BCKDK	2,65	2,57	2,62	-0,37
BHLHE40	15,24	4,45	4,75	0,36
BNIP3	19,42	6,13	5,19	1,93
BNIP3L	14,12	4,00	2,50	2,96
C4orf3	7,44	1,67	2,41	-1,15
C8orf58	3,11	0,03	0,91	-0,87
CCNG2	7,33	1,52	-0,11	1,47
CLK3	3,56	-0,01	1,31	-1,63
CRKL	2,55	1,09	-0,23	1,52
DOLK	-3,46	-0,13	-1,10	1,13
EFNA3	4,21	4,15	2,24	3,32
EGLN1	9,59	5,89	4,73	1,84
ENO1	6,10	7,28	5,64	1,59
ENO2	14,33	2,88	2,41	0,09
ERO1A	9,70	4,34	4,00	0,52
FAM162A	8,34	6,16	6,66	-0,97
FBXO42	2,26	-0,73	1,15	-2,10
FLAD1	-3,29	-2,12	-0,37	-2,16
FUT11	11,17	3,89	3,22	1,21
GAPDH	5,05	4,21	4,30	-0,52
GBE1	9,71	3,73	2,33	1,21
GPI	8,55	5,48	5,33	-0,55
GYS1	4,92	0,40	0,35	0,01
HILPDA	16,71	4,75	4,29	0,57
INSIG2	13,56	3,16	1,76	1,90
KDM3A	10,85	3,87	2,50	2,83
KDM4B	3,65	-0,51	0,88	-1,48
KDM4C	2,26	1,17	1,17	0,13
LDHA	7,65	8,52	7,46	0,84
MAPK7	2,63	-1,83	-3,03	1,22
MPI	3,50	3,00	4,75	-3,20
MXI1	10,86	0,33	1,21	-1,44
NARF	4,10	2,47	2,35	-0,06
NCKIPSD	2,34	5,86	6,19	-1,08
NDRG1	22,09	4,49	2,22	1,93
NGLY1	3,50	0,73	2,60	-2,45
P4HA1	11,07	3,73	3,03	1,65
PDK1	13,12	4,59	4,16	0,66
PDK3	4,40	4,55	3,71	0,54
PFKFB3	14,55	6,35	3,71	2,38
PFKL	3,54	0,59	0,36	0,23

PFKP	9,23	5,15	4,18	1,64
PGAM1	6,38	2,57	0,76	2,25
PGK1	9,99	6,83	4,76	3,00
PGM1	7,89	5,85	3,54	3,04
PKM	4,75	4,23	4,87	-1,13
PLOD1	6,61	2,47	2,22	0,03
POP4	-2,31	1,76	0,30	1,54
PPP2R5B	3,48	-0,04	-1,89	2,15
QRSL1	-2,25	-3,01	-1,04	-2,35
RAB20	4,26	2,34	2,39	0,16
RBPJ	3,85	2,69	1,41	1,23
RIOK3	3,73	2,58	1,94	0,06
RLF	7,07	2,26	0,73	2,21
RNF24	3,85	-1,10	-2,37	1,56
RORA	4,51	2,86	4,36	-1,04
SAP30	2,91	1,63	2,03	-0,41
SEC61G	4,74	3,89	1,88	1,73
SLC25A36	5,33	-0,44	-0,83	0,54
SLC2A1	8,68	4,61	4,77	0,21
TMX2	-3,70	0,43	0,58	-0,14
VKORC1	5,42	6,89	6,20	0,70
WDR45B	4,50	-1,17	-2,66	1,80
WDR54	5,70	3,88	3,42	-0,28
WSB1	7,02	-0,83	1,03	-2,00
YEATS2	5,35	4,77	5,05	-0,49
ZBTB25	3,42	0,95	3,63	-3,90
ZMPSTE24	-4,39	-0,13	-1,47	1,90
ZNF160	2,71	2,31	3,43	-2,42
ZNF292	6,02	3,08	3,33	-1,01
ZNF395	8,06	2,95	2,12	1,60
ZNF654	4,29	4,22	3,49	0,07

Supplementary Table S7.

patient ID	gene_symbol	Chromosome	Start_Position	Variant_Classification	Reference_Allele	Variant Allele	HGVSc	HGVSp	Transcript_ID	VAF
241	KRAS	12	25227348	missense_variant	G	C	c.176C>G	p.Ala59Gly	ENST00000256078	0,053
241	NOTCH1	9	136496196	frameshift_variant	CAG	C	c.7541_7542del	p.Pro2514ArgfsTer4	ENST00000277541	0,251
536	ATM	11	108295038	missense_variant	G	A	c.4888G>A	p.Asp1630Asn	ENST00000278616	0,975
536	NOTCH1	9	136495700	3_prime_UTR_variant	T	C	c.*371A>G	.	ENST00000277541	0,414
604	BIRC3	11	102331153	frameshift_variant	A	AT	c.1236_1237insT	p.Val413CysfsTer3	ENST00000263464	0,185
636	RPS15	19	1440424	missense_variant	G	A	c.421G>A	p.Gly141Arg	ENST00000593052	0,073
912	ATM	11	108256267	missense_variant	T	G	c.2177T>G	p.Leu726Arg	ENST00000278616	0,389
912	ATM	11	108329211	missense_variant	T	G	c.7280T>G	p.Leu2427Arg	ENST00000278616	0,486
1003	KRAS	12	25245347	missense_variant	C	T	c.38G>A	p.Gly13Asp	ENST00000256078	0,193
1085	BIRC3	11	102331211	frameshift_variant	A	AG	c.1295dup	p.Glu433ArgfsTer5	ENST00000263464	0,107
1085	BIRC3	11	102336925	frameshift_variant	AC	A	c.1639del	p.Gln547AsnfsTer21	ENST00000263464	0,184
1113	NFKBIE	6	44265001	frameshift_variant	TGTAA	T	c.759_762del	p.Tyr254SerfsTer13	ENST00000275015	0,444
1113	NRAS	1	114713909	missense_variant	G	T	c.181C>A	p.Gln61Lys	ENST00000369535	0,39
1120	ATM	11	108317413	missense_variant	A	G	c.6239A>G	p.Tyr2080Cys	ENST00000278616	0,983
1121	ATM	11	108343321	stop_gained	A	T	c.8368A>T	p.Arg2790Ter	ENST00000278616	0,155
1141	NFKBIE	6	44265001	frameshift_variant	TGTAA	T	c.759_762del	p.Tyr254SerfsTer13	ENST00000275015	0,088
1141	NRAS	1	114713909	missense_variant	G	T	c.181C>A	p.Gln61Lys	ENST00000369535	0,102
1191	ATM	11	108330247	missense_variant	G	C	c.7341G>C	p.Leu2447Phe	ENST00000278616	0,349
1191	NOTCH1	9	136496196	frameshift_variant	CAG	C	c.7541_7542del	p.Pro2514ArgfsTer4	ENST00000277541	0,422
1191	SF3B1	2	197402110	missense_variant	T	C	c.2098A>G	p.Lys700Glu	ENST00000335508	0,47
1200	KRAS	12	25225623	missense_variant	C	G	c.441G>C	p.Lys147Asn	ENST00000256078	0,129
1200	NFKBIE	6	44265021	frameshift_variant	TGCTGAGG	T	c.736_742del	p.Pro246SerfsTer20	ENST00000275015	0,414
1200	NOTCH1	9	136496196	frameshift_variant	CAG	C	c.7541_7542del	p.Pro2514ArgfsTer4	ENST00000277541	0,486
1203	ATM	11	108229218	stop_gained	A	T	c.226A>T	p.Arg76Ter	ENST00000278616	0,98
1203	RB1	13	48342598	splice_acceptor_variant	G	A	c.265-1G>A	p.?	ENST00000267163	0,905

1218	BRAF	7	140781602	missense_variant	C	G	c.1406G>C	p.Gly469Ala	ENST00000646891	0,459
1272	SF3B1	2	197400753	missense_variant	C	T	c.2680G>A	p.Asp894Asn	ENST00000335508	0,397
1274	NOTCH1	9	136495693	3_prime_UTR_variant	T	C	c.*378A>G	.	ENST00000277541	0,074
1274	NOTCH1	9	136496196	frameshift_variant	CAG	C	c.7541_7542del	p.Pro2514ArgfsTer4	ENST00000277541	0,452
1344	SF3B1	2	197402110	missense_variant	T	C	c.2098A>G	p.Lys700Glu	ENST00000335508	0,462
1358	ATM	11	108247128	splice_donor_variant	G	C	c.1065+1G>C	p.?	ENST00000278616	0,947
1358	BRAF	7	140753393	missense_variant&splice_region_variant	T	A	c.1742A>T	p.Asn581Ile	ENST00000646891	0,203
1358	RPS15	19	1440459	missense_variant	G	T	c.456G>T	p.Lys152Asn	ENST00000593052	0,459
1377	RB1	13	48362859	stop_gained	C	T	c.763C>T	p.Arg255Ter	ENST00000267163	0,442
1380	NOTCH1	9	136496838	frameshift_variant	A	AC	c.6900dup	p.Ser2301ValfsTer53	ENST00000277541	0,989
1380	SF3B1	2	197402636	missense_variant	T	G	c.1997A>C	p.Lys666Thr	ENST00000335508	0,05
1412	BRAF	7	140753355	missense_variant	C	T	c.1780G>A	p.Asp594Asn	ENST00000646891	0,478
1412	NOTCH1	9	136496196	frameshift_variant	CAG	C	c.7541_7542del	p.Pro2514ArgfsTer4	ENST00000277541	0,5
1485	BIRC3	11	102331193	frameshift_variant	G	GA	c.1279dup	p.Ile427AsnfsTer11	ENST00000263464	0,911
1485	NOTCH1	9	136496196	frameshift_variant	CAG	C	c.7541_7542del	p.Pro2514ArgfsTer4	ENST00000277541	0,532
1524	BRAF	7	140753334	missense_variant	T	C	c.1801A>G	p.Lys601Glu	ENST00000646891	0,318
1531	RB1	13	48476758	stop_gained	A	T	c.2578A>T	p.Lys860Ter	ENST00000267163	0,103
1531	RB1	13	48476761	missense_variant	A	G	c.2581A>G	p.Arg861Gly	ENST00000267163	0,103
1536	SF3B1	2	197401887	missense_variant&splice_region_variant	C	T	c.2225G>A	p.Gly742Asp	ENST00000335508	0,465
1546	NOTCH1	9	136495700	3_prime_UTR_variant	T	C	c.*371A>G	.	ENST00000277541	0,45
1596	SF3B1	2	197402097	missense_variant	A	C	c.2111T>G	p.Ile704Ser	ENST00000335508	0,356
1751	ATM	11	108330303	missense_variant	C	T	c.7397C>T	p.Ala2466Val	ENST00000278616	0,975
1751	NFKBIE	6	44260518	frameshift_variant	G	GC	c.1129dup	p.Ala377GlyfsTer19	ENST00000275015	0,055
1775	ATM	11	108335942	missense_variant	T	C	c.8249T>C	p.Leu2750Ser	ENST00000278616	0,996
1775	BIRC3	11	102331211	frameshift_variant	A	AG	c.1295dup	p.Glu433ArgfsTer5	ENST00000263464	0,29
1775	SF3B1	2	197402098	missense_variant	T	A	c.2110A>T	p.Ile704Phe	ENST00000335508	0,077
1843	ATM	11	108229193	stop_gained	T	G	c.201T>G	p.Tyr67Ter	ENST00000278616	0,485
1843	ATM	11	108365383	missense_variant	A	G	c.9046A>G	p.Lys3016Glu	ENST00000278616	0,493
1865	ATM	11	108316103	missense_variant	G	A	c.6188G>A	p.Gly2063Glu	ENST00000278616	0,995

1869	NFKBIE	6	44265001	frameshift_variant	TGTAA	T	c.759_762del	p.Tyr254SerfsTer13	ENST00000275015	0,419
1869	NFKBIE	6	44265002	stop_gained&frameshift_variant	G	GT	c.761dup	p.Tyr254Ter	ENST00000275015	0,357
1869	NFKBIE	6	44265129	frameshift_variant	TAGGTGGA	T	c.628_634del	p.Ser210MetfsTer33	ENST00000275015	0,173
1873	NOTCH1	9	136496526	stop_gained	G	A	c.7213C>T	p.Gln2405Ter	ENST00000277541	0,508
1931	SF3B1	2	197402110	missense_variant	T	C	c.2098A>G	p.Lys700Glu	ENST00000335508	0,509
1976	ATM	11	108297365	missense_variant	G	T	c.4988G>T	p.Gly1663Val	ENST00000278616	0,99
2733	ATM	11	108249054	missense_variant	T	A	c.1187T>A	p.Ile396Lys	ENST00000278616	0,985

Supplementary table S8.

Patient ID	Gene_symbol	Chromosome	Start_Position	Variant_Classification	Reference_Allele	Variant Allele	HGVSc	HGVSp	Transcript_ID	VAF
1980	AAK1	2	69509361	missense_variant	T	C	c.1876A>G	p.Lys626Glu	ENST00000409085	0,20
1980	AL590132.1	1	210861169	missense_variant	T	G	c.1278A>C	p.Glu426Asp	ENST00000639602	0,35
264	ALK	2	29328461	missense_variant	T	A	c.1303A>T	p.Met435Leu	ENST00000389048	0,33
693	ALKBH2	12	109088350	stop_gained	G	C	c.443C>G	p.Ser148Ter	ENST00000440112	0,21
264	ANKAR	2	189743365	missense_variant	A	G	c.3901A>G	p.Asn1301Asp	ENST00000520309	0,45
264	ARHGEF40	14	21081770	missense_variant	C	T	c.2902C>T	p.Arg968Trp	ENST00000298694	0,58
264	ATP10B	5	160632298	missense_variant	G	A	c.1451C>T	p.Ser484Phe	ENST00000327245	0,44
1980	C1orf56	1	151048582	missense_variant	C	A	c.735C>A	p.His245Gln	ENST00000368926	0,46
636	CABP1	12	120666895	missense_variant	C	T	c.1108C>T	p.Arg370Cys	ENST00000316803	0,65
1980	CHD2	15	92978331	missense_variant	A	T	c.2675A>T	p.Gln892Leu	ENST00000394196	0,16
693	CHKB	22	50582256	missense_variant	A	G	c.326T>C	p.Ile109Thr	ENST00000406938	0,16
1980	CLCA2	1	86438922	missense_variant	T	C	c.1019T>C	p.Met340Thr	ENST00000370565	0,18
264	CNN2	19	1037641	missense_variant	C	T	c.734C>T	p.Pro245Leu	ENST00000562958	0,20
1869	CRP	1	159713596	missense_variant	C	T	c.604G>A	p.Val202Ile	ENST00000255030	0,18
1869	DCAF1	3	51403329	missense_variant	G	A	c.4279C>T	p.Leu1427Phe	ENST00000423656	0,34
1869	DCN	12	91153183	missense_variant	G	T	c.659C>A	p.Pro220His	ENST00000052754	0,16
1980	DICER1	14	95091302	missense_variant	C	T	c.5428G>A	p.Asp1810Asn	ENST00000526495	0,20
264	DNAH2	17	7740452	missense_variant	T	A	c.1409T>A	p.Met470Lys	ENST00000572933	0,35
693	EPYC	12	90978160	missense_variant	G	A	c.268C>T	p.Pro90Ser	ENST00000261172	0,18
264	ETV3	1	157124947	missense_variant	C	T	c.1433G>A	p.Arg478Gln	ENST00000368192	0,32
264	FCHSD1	5	141651041	missense_variant	G	A	c.98C>T	p.Ala33Val	ENST00000435817	0,26
636	FRYL	4	48582502	missense_variant	T	C	c.1981A>G	p.Thr661Ala	ENST00000358350	0,44
264	HGF	7	81756067	splice_region_variant&intron_variant	C	T	c.483-4G>A	p.?	ENST00000354224	0,44
264	HMGN5	X	81114736	missense_variant	T	A	c.762A>T	p.Glu254Asp	ENST00000358130	0,61

264	HMMR	5	163490419	frameshift_variant	T	TA	c.2001dup	p.Gln668ThrfsTer3	ENST00000393915	0,16
1980	HOXA3	7	27110570	missense_variant	A	C	c.71T>G	p.Phe24Cys	ENST00000612286	0,16
1869	IFT140	16	1511060	missense_variant	G	A	c.4273C>T	p.Arg1425Trp	ENST00000426508	0,46
264	IGLL5	22	22888262	splice_region_variant&intron_variant	A	T	c.206+3A>T	p.?	ENST00000526893	0,47
264	IGLL5	22	22888260	splice_donor_variant	G	A	c.206+1G>A	p.?	ENST00000526893	0,54
636	INSYN2A	10	127175301	missense_variant	C	T	c.1095G>A	p.Met365Ile	ENST00000522781	0,56
1980	KANK3	19	8335667	missense_variant	C	G	c.160G>C	p.Glu54Gln	ENST00000330915	0,50
1869	KDM7A	7	140091924	missense_variant	T	C	c.2611A>G	p.Ile871Val	ENST00000397560	0,45
693	KIR3DL3	19	54725248	inframe_deletion&splice_region_variant	GTTC	G	c.42_44del	p.Phe14del	ENST00000291860	0,40
636	KLHL25	15	85768276	missense_variant	T	A	c.1535A>T	p.Asp512Val	ENST00000337975	0,36
1869	KRT18	12	52949184	missense_variant	C	T	c.11C>T	p.Thr4Ile	ENST00000388837	0,25
1869	KRT18	12	52949223	missense_variant	G	A	c.50G>A	p.Gly17Asp	ENST00000388837	0,27
264	LGR6	1	202318599	missense_variant	;	A	c.2296G>A	p.Val766Met	ENST00000367278	0,27
1869	LOC105374299	3	195817428	missense_variant	G	A	c.1714G>A	p.Gly572Ser	XM_011513362.2	0,30
1980	LOC107984590	13	113011787	missense_variant	T	C	c.1325A>G	p.Gln442Arg	XM_017020903.1	0,42
264	LOC107985433	20	30287394	missense_variant	C	A	c.314G>T	p.Cys105Phe	XM_017028192.1	0,33
264	LOXL2	8	23298922	missense_variant	A	T	c.2159T>A	p.Val720Asp	ENST00000389131	0,22
264	LRFN5	14	41891515	missense_variant	C	T	c.1651C>T	p.Arg551Trp	ENST00000298119	0,51
264	LRP1B	2	141247344	missense_variant	T	A	c.474A>T	p.Glu158Asp	ENST00000389484	0,48
1980	LRRC8D	1	89933740	missense_variant	C	G	c.672C>G	p.Asn224Lys	ENST00000337338	0,60
636	MED12	X	71119403	missense_variant	G	A	c.130G>A	p.Gly44Ser	ENST00000374080	0,24
636	MED12	X	71119357	splice_acceptor_variant	CGCTTTCCTGCCTCAGGATGAACTGACGGC	TT	c.100-16_113delinsTT	p.?	ENST00000374080	0,77
1869	MED12	X	71119380	missense_variant	T	G	c.107T>G	p.Leu36Arg	ENST00000374080	0,50
264	MED12L	3	151430325	inframe_deletion	CCAG	C	c.6343_6345del	p.Gln2115del	ENST00000474524	0,15
636	MMS22L	6	97229201	missense_variant	G	A	c.1732C>T	p.Leu578Phe	ENST00000275053	0,49
636	MRPS5	2	95108249	missense_variant	T	C	c.563A>G	p.Lys188Arg	ENST00000272418	0,48
636	MT-CO3	M	9247	missense_variant	G	A	c.41G>A	p.Ser14Asn	ENST00000362079	0,17

1869	MUC5AC	11	1190453	missense_variant	C	T	c.12308C>T	p.Thr4103Ile	ENST00000621226	0,15
1869	MUC5AC	11	1190429	missense_variant	C	G	c.12284C>G	p.Thr4095Ser	ENST00000621226	0,19
264	MYD88	3	38141150	stop_lost	T	C	c.574T>C	p.Ter192ArgextTer8	ENST00000443433	0,50
636	MYH2	17	10525019	missense_variant	T	C	c.4709A>G	p.Glu1570Gly	ENST00000397183	0,19
1869	MYO9B	19	17200777	missense_variant	A	G	c.4511A>G	p.Gln1504Arg	ENST00000595618	0,53
1980	MYT1L	2	1892152	missense_variant	G	A	c.2168C>T	p.Thr723Met	ENST00000647738	0,48
1869	NFKBIE	6	44265002	stop_gained&frameshift_variant	G	GT	c.761dup	p.Tyr254Ter	ENST00000275015	0,24
1869	NFKBIE	6	44265129	frameshift_variant	TAGGTGGA	T	c.628_634del	p.Ser210MetfsTer33	ENST00000275015	0,26
1869	NFKBIE	6	44265001	frameshift_variant	TGTAA	T	c.759_762del	p.Tyr254SerfsTer13	ENST00000275015	0,38
636	NYNRIN	14	24416709	frameshift_variant	T	TAC	c.4969_4970dup	p.Ala1658ArgfsTer19	ENST00000382554	0,50
1869	OR10K2	1	158420226	missense_variant	A	T	c.641T>A	p.Ile214Asn	ENST00000641042	0,21
636	OR2A12	7	144095843	missense_variant	G	A	c.736G>A	p.Val246Met	ENST00000641592	0,45
636	OR8U1	11	56375745	missense_variant	G	T	c.122G>T	p.Gly41Val	ENST00000302270	0,48
693	PAX4	7	127610907	missense_variant	G	A	c.992C>T	p.Ala331Val	ENST00000378740	0,43
264	PCDHA6	5	140828192	missense_variant	C	A	c.101C>A	p.Ser34Tyr	ENST00000529310	0,46
264	PCLO	7	83154752	missense_variant	G	A	c.1889C>T	p.Thr630Met	ENST00000333891	0,33
1869	PDE9A	21	42760889	missense_variant	A	G	c.1067A>G	p.His356Arg	ENST00000291539	0,47
1869	PGM2L1	11	74343347	missense_variant	G	T	c.1288C>A	p.Leu430Ile	ENST00000298198	0,43
693	PID1	2	229262884	missense_variant	C	G	c.93G>C	p.Leu31Phe	XM_017004404.1	0,47
1980	PID1	2	229025699	missense_variant	T	G	c.686A>C	p.Asp229Ala	ENST00000354069	0,50
264	PKHD1L1	8	109490020	missense_variant	C	T	c.9949C>T	p.Pro3317Ser	ENST00000378402	0,32
693	POLR2A	17	7508417	missense_variant	C	T	c.3407C>T	p.Thr1136Ile	NM_000937.5	0,18
1869	PRSS2	7	142772607	splice_region_variant&intron_variant	A	G	c.242+4A>G	p.?	ENST00000633969	0,68
1869	PTPN13	4	86750770	missense_variant	A	G	c.2951A>G	p.Asn984Ser	ENST00000436978	0,16
636	RBM39	20	35732104	inframe_deletion	GACT	G	c.130_132del	p.Ser44del	ENST00000253363	0,34
264	RNF34	12	121430237	missense_variant	A	C	c.1267A>C	p.Lys423Gln	ENST00000392464	0,24
1980	RP1L1	8	10609282	missense_variant	G	A	c.4816C>T	p.Arg1606Cys	ENST00000382483	0,45
1869	SALL1	16	51137521	missense_variant	G	T	c.3566C>A	p.Thr1189Asn	ENST00000251020	0,21

1869	SCARA3	8	27659222	missense_variant	C	T	c.1052C>T	p.Ala351Val	ENST00000301904	0,52
264	SEC24A	5	134649149	missense_variant	G	A	c.73G>A	p.Ala25Thr	ENST00000398844	0,19
1869	SLC23A2	20	4869922	frameshift_variant	T	TG	c.1233dup	p.Ile412HisfsTer18	ENST00000379333	0,61
693	SLC23A3	2	219167981	stop_gained	G	A	c.886C>T	p.Gln296Ter	ENST00000455516	0,19
693	SSTR1	14	38209970	missense_variant	T	A	c.581T>A	p.Val194Asp	ENST00000267377	0,25
1980	TAS2R19	12	11021703	missense_variant	A	G	c.869T>C	p.Phe290Ser	ENST00000390673	0,17
1869	TBX21	17	47744831	frameshift_variant	TC	T	c.1075del	p.Leu359TyrfsTer46	ENST00000177694	0,18
264	TECPR1	7	98229137	missense_variant	C	T	c.2312G>A	p.Arg771Gln	ENST00000447648	0,50
693	TMC6	17	78120677	missense_variant	G	A	c.1691C>T	p.Thr564Met	ENST00000590602	0,17
1869	TNK2	3	195868364	missense_variant	G	A	c.2123C>T	p.Pro708Leu	ENST00000381916	0,50
1980	TRIM64B	11	89870749	missense_variant	C	T	c.1222G>A	p.Val408Met	ENST00000329862	0,30
693	TRIM7	5	181205095	missense_variant	G	A	c.16C>T	p.Pro6Ser	ENST00000274773	0,27
264	TTYH3	7	2656134	missense_variant	G	T	c.1063G>T	p.Val355Leu	ENST00000258796	0,49
264	ULK4	3	41754466	missense_variant	C	T	c.2216G>A	p.Arg739His	ENST00000301831	0,36
264	WASF3	13	26681319	missense_variant&splice_region_variant	G	A	c.982G>A	p.Gly328Arg	ENST00000335327	0,50
1980	WDR27	6	169602218	splice_donor_variant	C	T	c.2424+1G>A	p.?	ENST00000448612	0,37
693	XPO1	2	61492192	missense_variant	T	C	c.1730A>G	p.His577Arg	ENST00000401558	0,29
1869	XPO4	13	20822283	stop_gained	G	A	c.847C>T	p.Arg283Ter	ENST00000255305	0,44
1869	YIPF6	X	68531943	missense_variant	G	A	c.655G>A	p.Val219Ile	ENST00000462683	0,41

Supplementary Figure S1.

A.1 PROFILE I

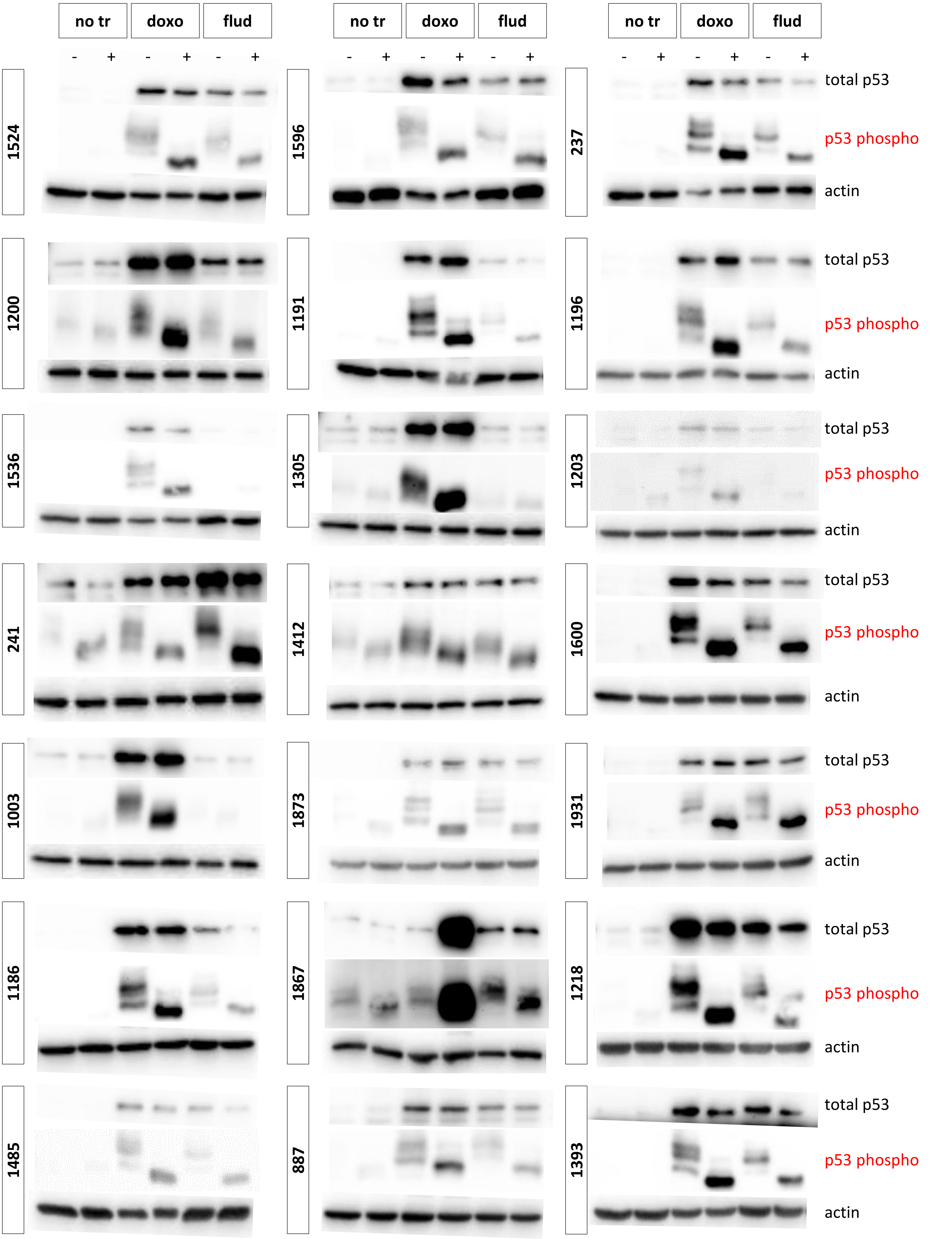

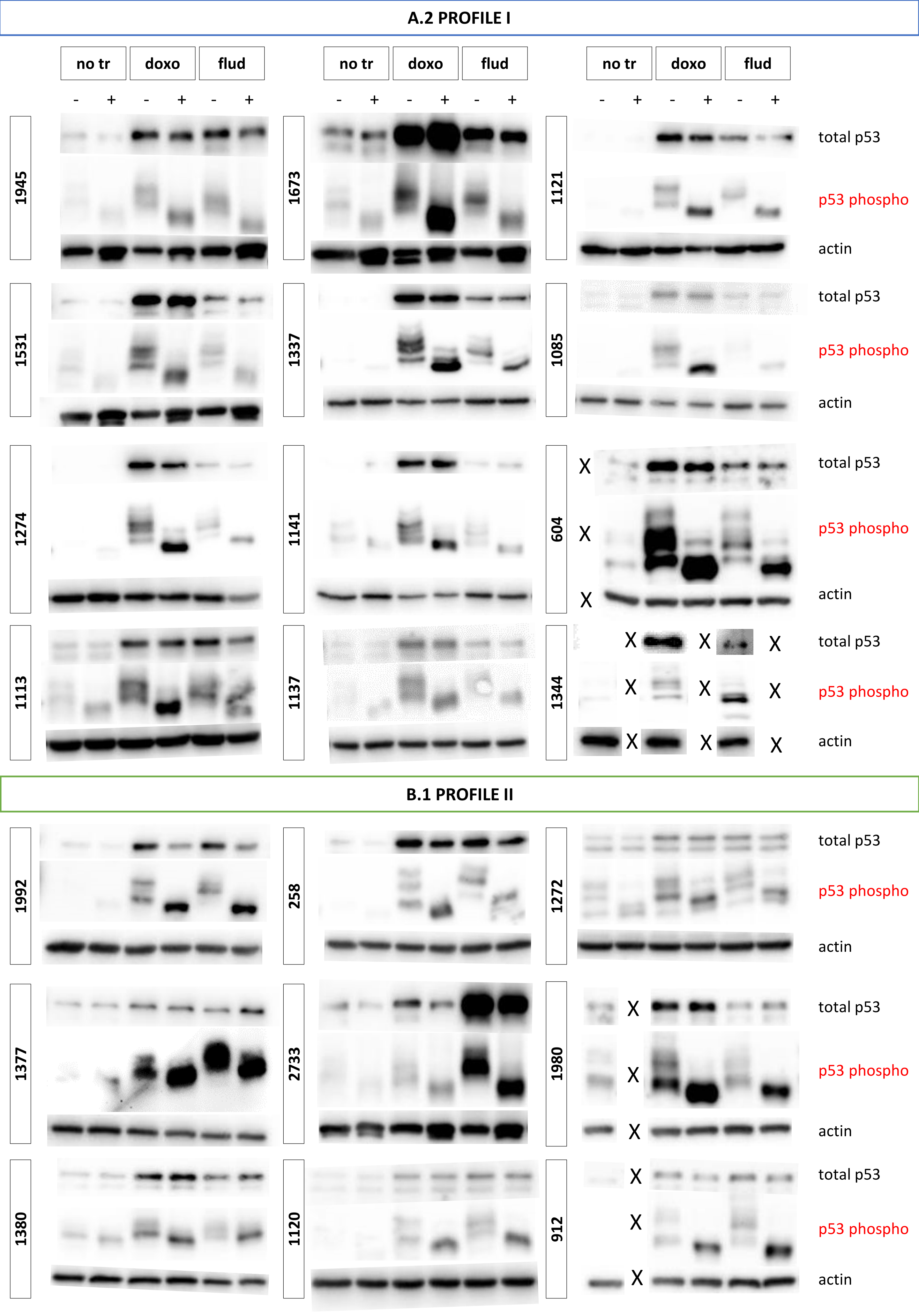
A.2 PROFILE I
no tr
doxo
flud
- + - + - +
1945
1673
1121
1531
1337
1085
1274
1141
604
1113
1137
1344
total p53
p53 phospho
actin
X
B.1 PROFILE II
1992
258
1272
1377
2733
1980
1380
1120
912

B.2 PROFILE II

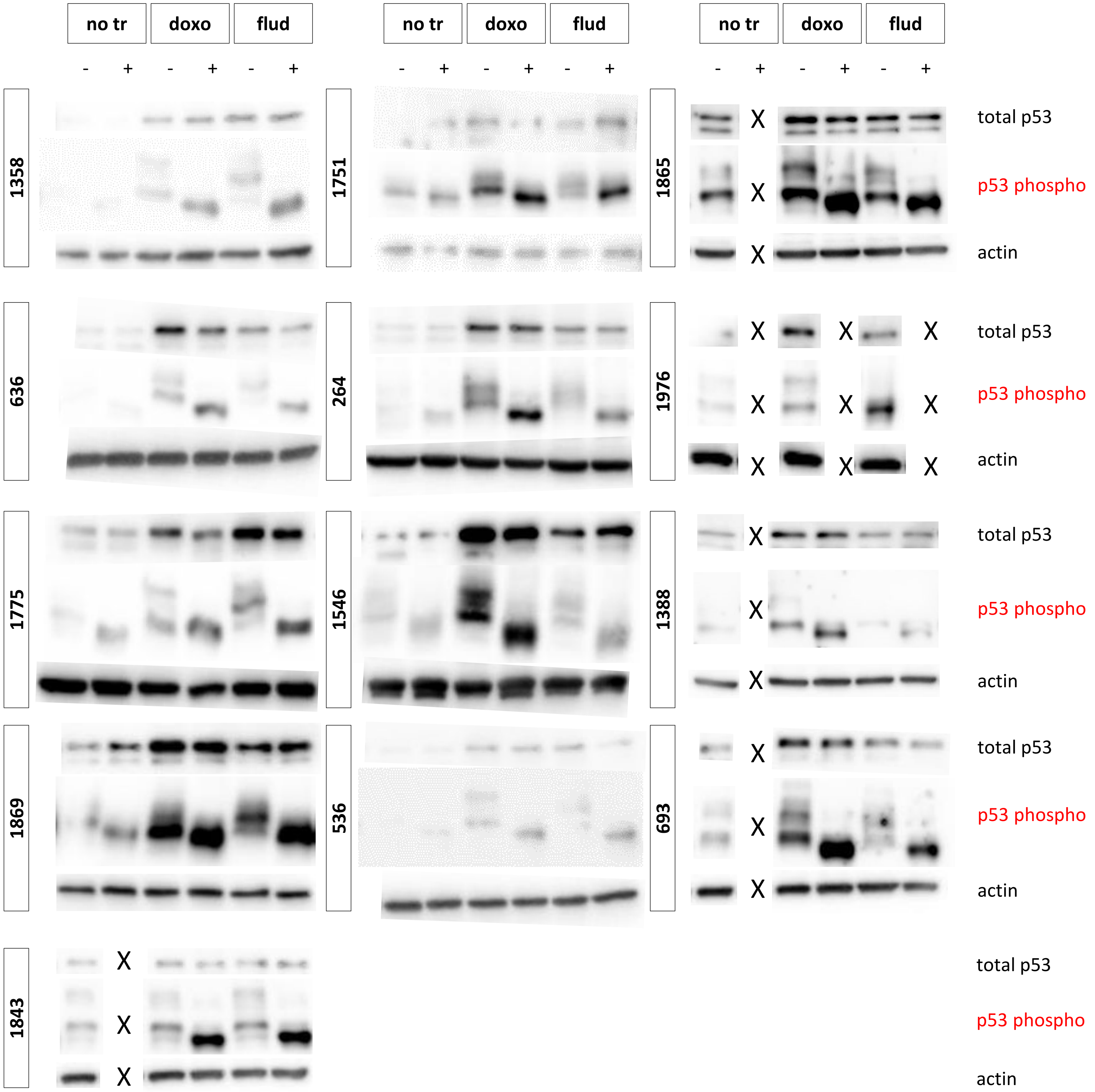

Supplementary Figure S2.

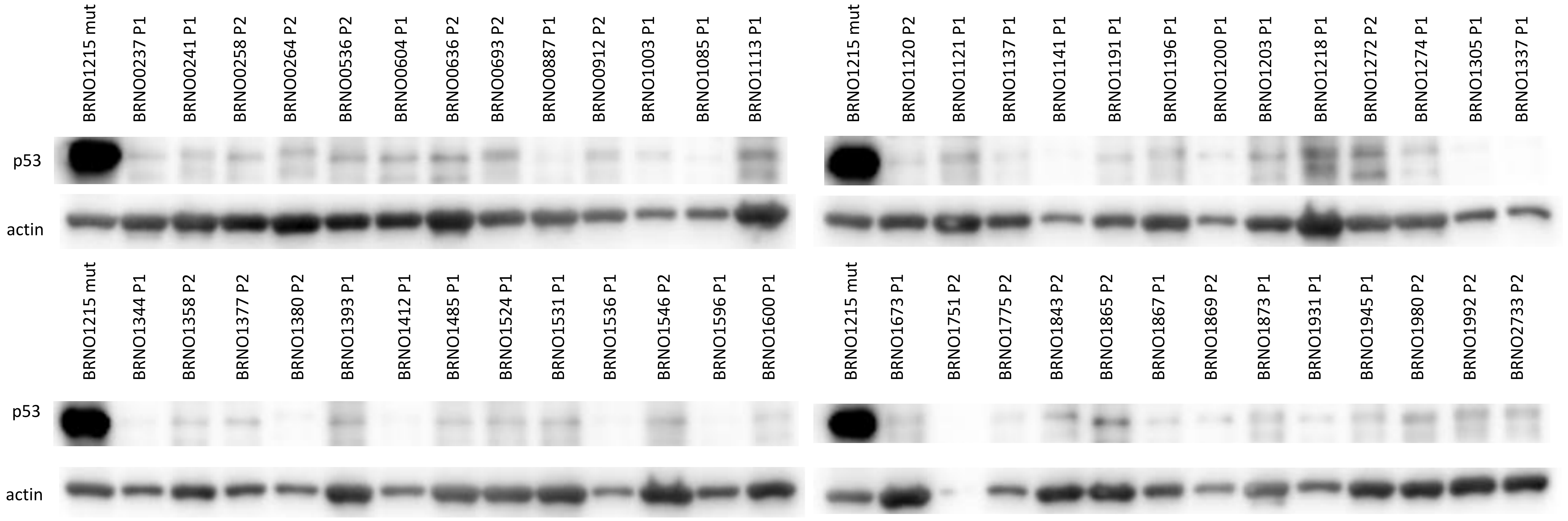

Supplementary Figure S3.

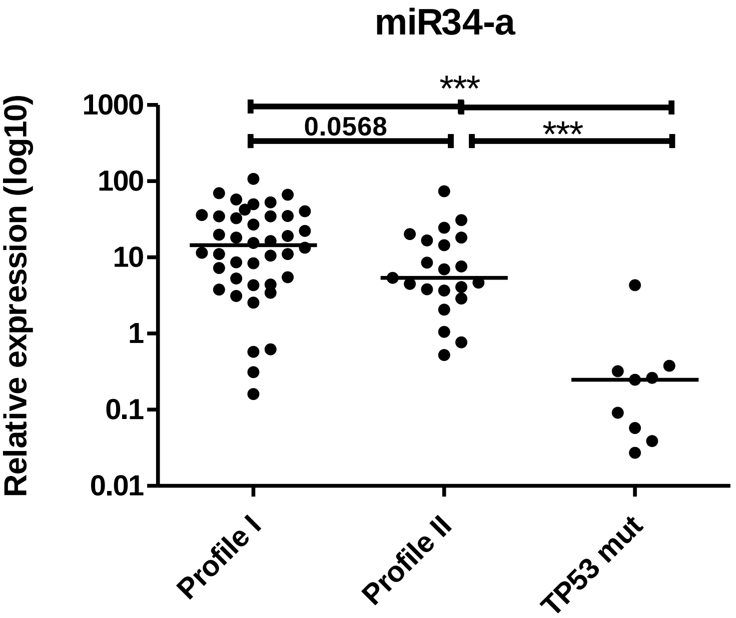

Supplementary Figure S4.

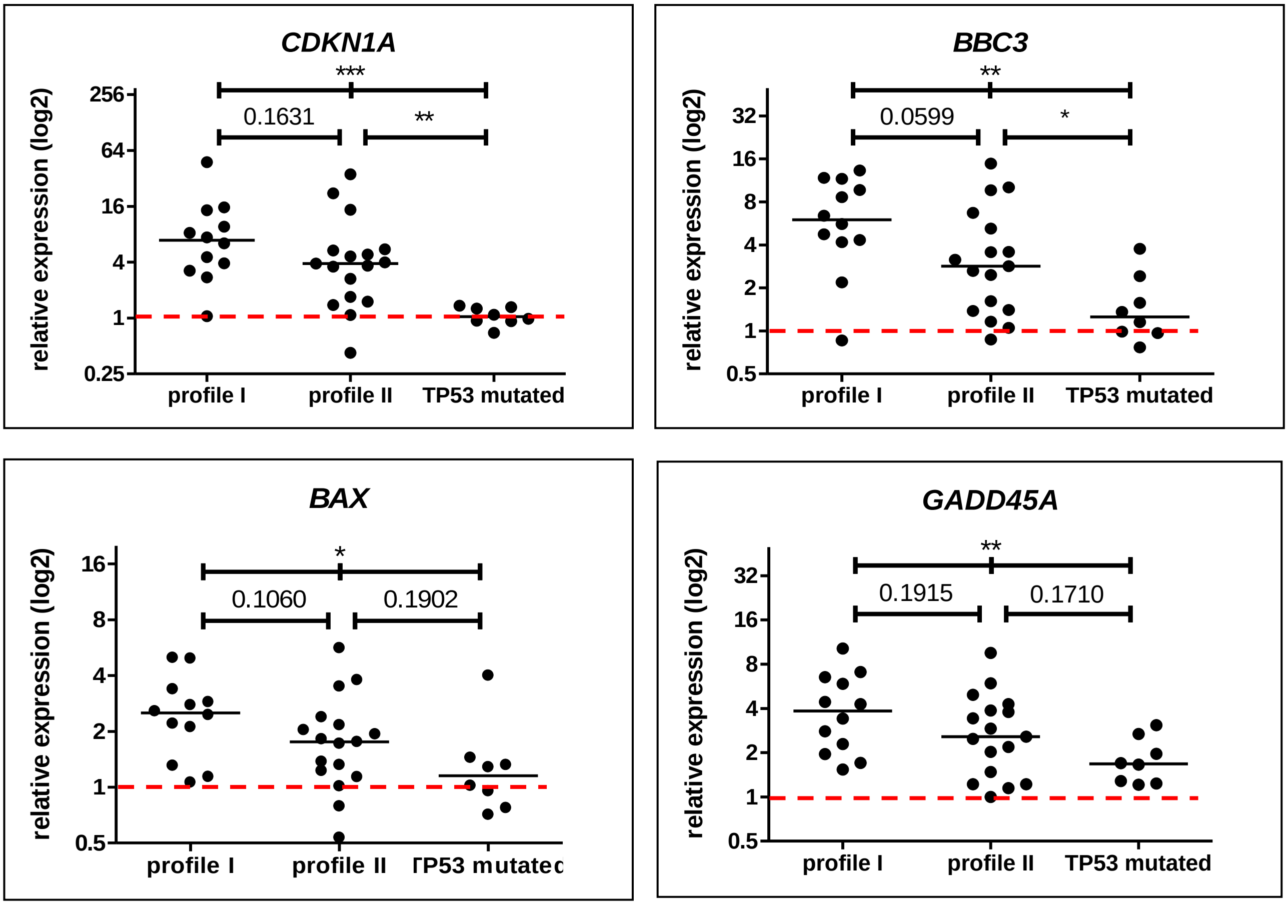

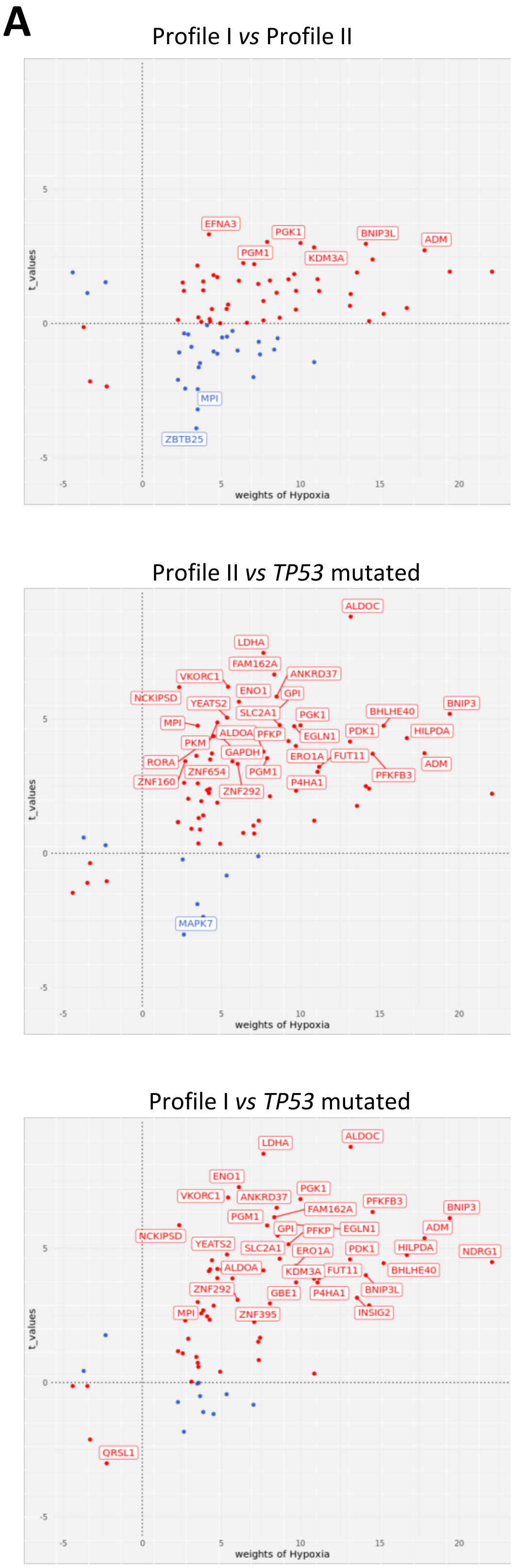

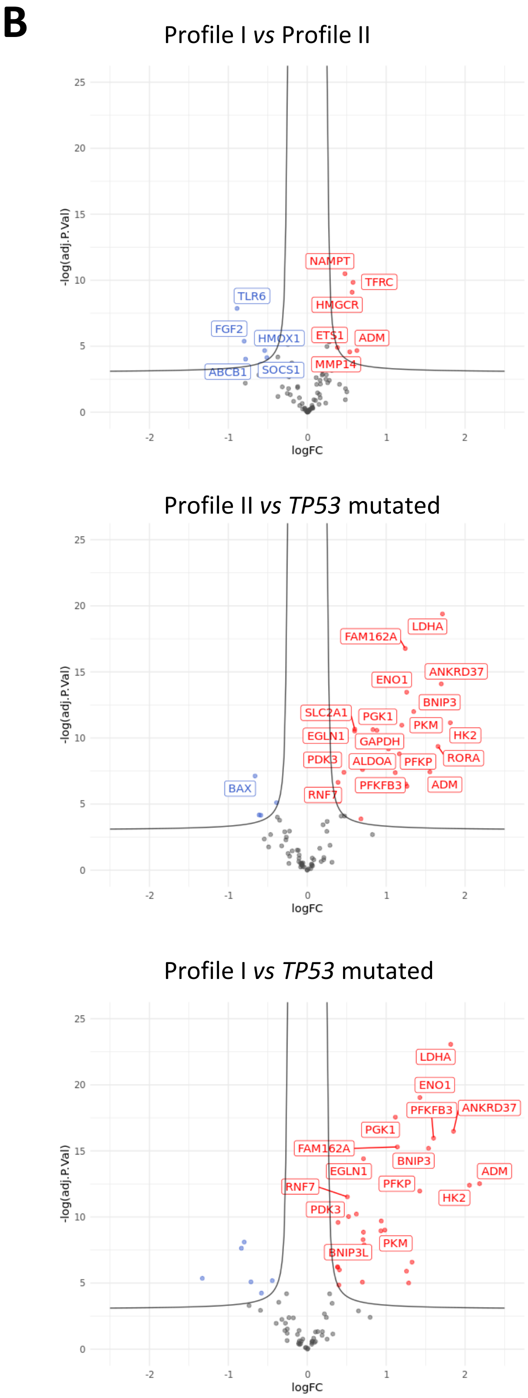

Profile I *vs* Profile II		Profile I *vs* *TP53*mut		Profile II *vs* *TP53*mut	
stat	Pval	stat	Pval	stat	Pval
-2,22	0,039	-5,75	1,91E-05	-3,00	0,008

Supplementary Figure S5.

A

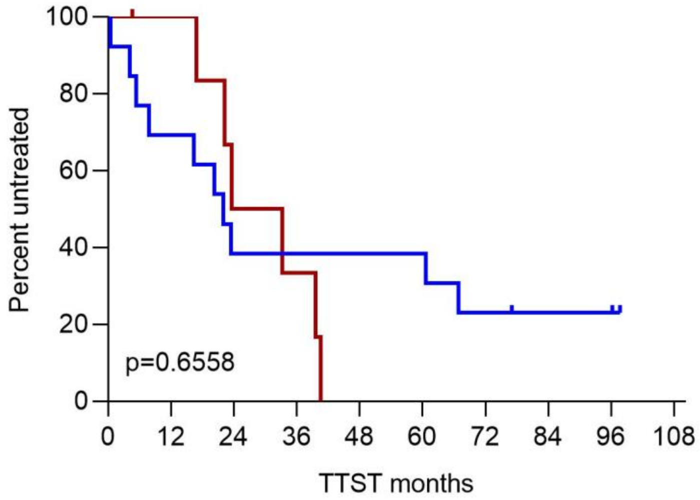

		n	Median TTST (months)
	Profile I	13	22
	Profile II	7	28.4

B

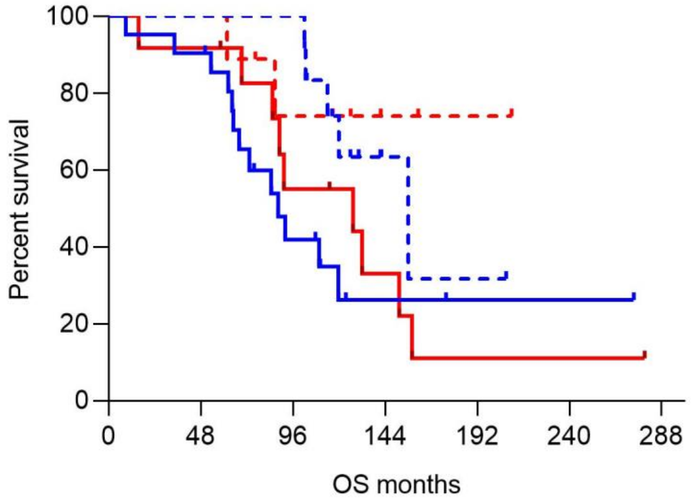

		Inhibitors	n	Median OS (months)
	Profile I	no	21	88.3
	Profile II	no	12	127.3
	Profile I	yes	12	156.1
	Profile II	yes	9	Undefined

Pairwise comparison	p value
vs.	0.5706
vs.	0.6414
vs.	0.0306
vs.	0.1052

Supplementary Figure S6.

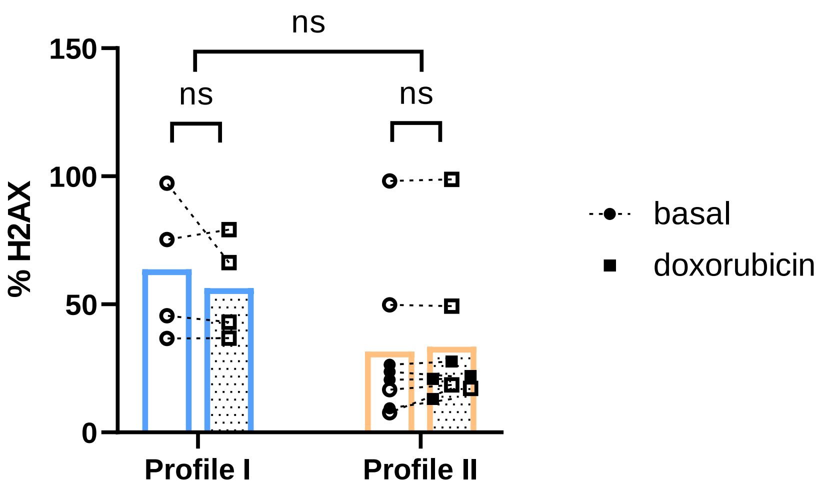

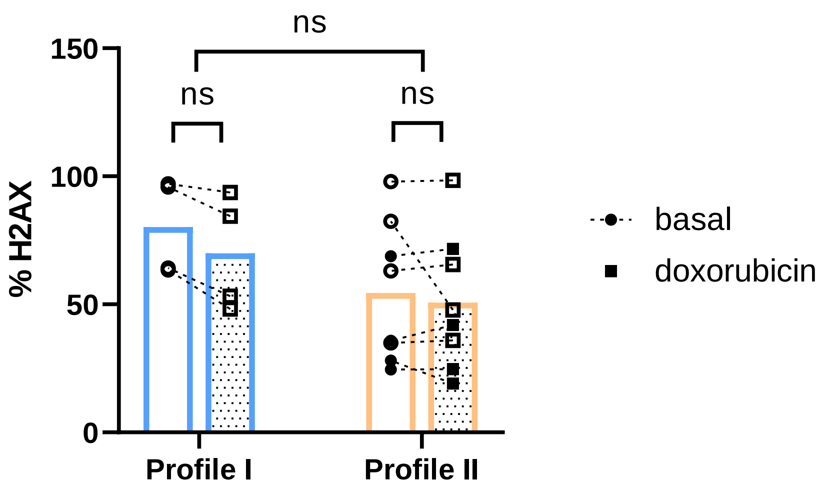

Supplementary Figure S7.
